# Coupled position and attitude control of a servicer spacecraft in rendezvous with an orbiting target

**DOI:** 10.1038/s41598-023-30687-9

**Published:** 2023-03-14

**Authors:** Ali Kasiri, Farhad Fani Saberi

**Affiliations:** 1 Department of Aerospace Engineering, Amirkabir University of Technology, Tehran, Iran; 2 Aerospace Sciences and Technology Institute, AMIRKABIR University of Technology, Tehran, Iran

**Keywords:** Aerospace engineering, Mechanical engineering

## Abstract

Rendezvous is one of the fundamental phases of on-orbit servicing (OOS) missions. Since it requires high accuracy and safety, modeling is an indispensable part. Therefore, this article puts forward an approach for boosting the exactitude of the final proximity phase of a servicer spacecraft using precise modeling. Unlike other similar works that solely use linear models to design controllers, this paper employs a fully nonlinear model and considers most possible uncertainties and disturbances. In this regard, first a complete nonlinear relative pose (i.e., concurrent position attitude) motion dynamic is developed, which includes (1) the role of the reaction wheels and (2) the major environmental force and torque model. Second, taking the thruster's adverse torque into account, two sliding mode-based control techniques with different nonlinear sliding surfaces are designed. Moreover, the Lyapunov stability criterion is used to handle high nonlinearity effects, control input saturation, actuator misalignment, external disturbance torque/force, measurement error, uncertainties of both inertia parameters, and control inputs. Even the PWPF modulator of the thrusters has been considered to make the outcomes more realistic. Finally, three different scenarios are comprehensively simulated to illustrate the feasibility and efficiency of the designed scheme. The results prove that the proposed closed-form controller is more executable to implement than other existing approaches.

## Introduction

Due to the continuous growth in space activities, in-orbit servicing missions (such as rescuing an out-of-control satellite^1^, in-orbit refueling^2^, replacing/repairing some faulty components^3^, active debris removal^4,5^, in space delivery^6^, inspecting a special spacecraft^7^, and on-orbit assembly^8^) will play an important role in the future of the space economy^9^. On the other hand, rendezvous and docking are inevitable and essential operating phases of the mentioned missions. The rendezvous is a series of orbital maneuvers in which two spacecraft, the chaser and the target, arrive in the same orbit and gently approach each other^10^. The rendezvous phase is usually followed by mating procedures in the next, which bring the chaser spacecraft into physical contact with the target. Indeed, at the end of the mating phase, the relative distance and velocity (both angular and translational) of the chaser and target spacecraft should be zero^11^. In general, rendezvous can be divided into three significant phases^12^; first: long-range approach also called “phasing”, second: close-range approach or approximation, and third: terminal phase or mating^11^. It is worth noting that each of the aforementioned stages has its own distinct sub-phase with unique requirements, constraints, and specific initial and final conditions.

**Figure 1:**
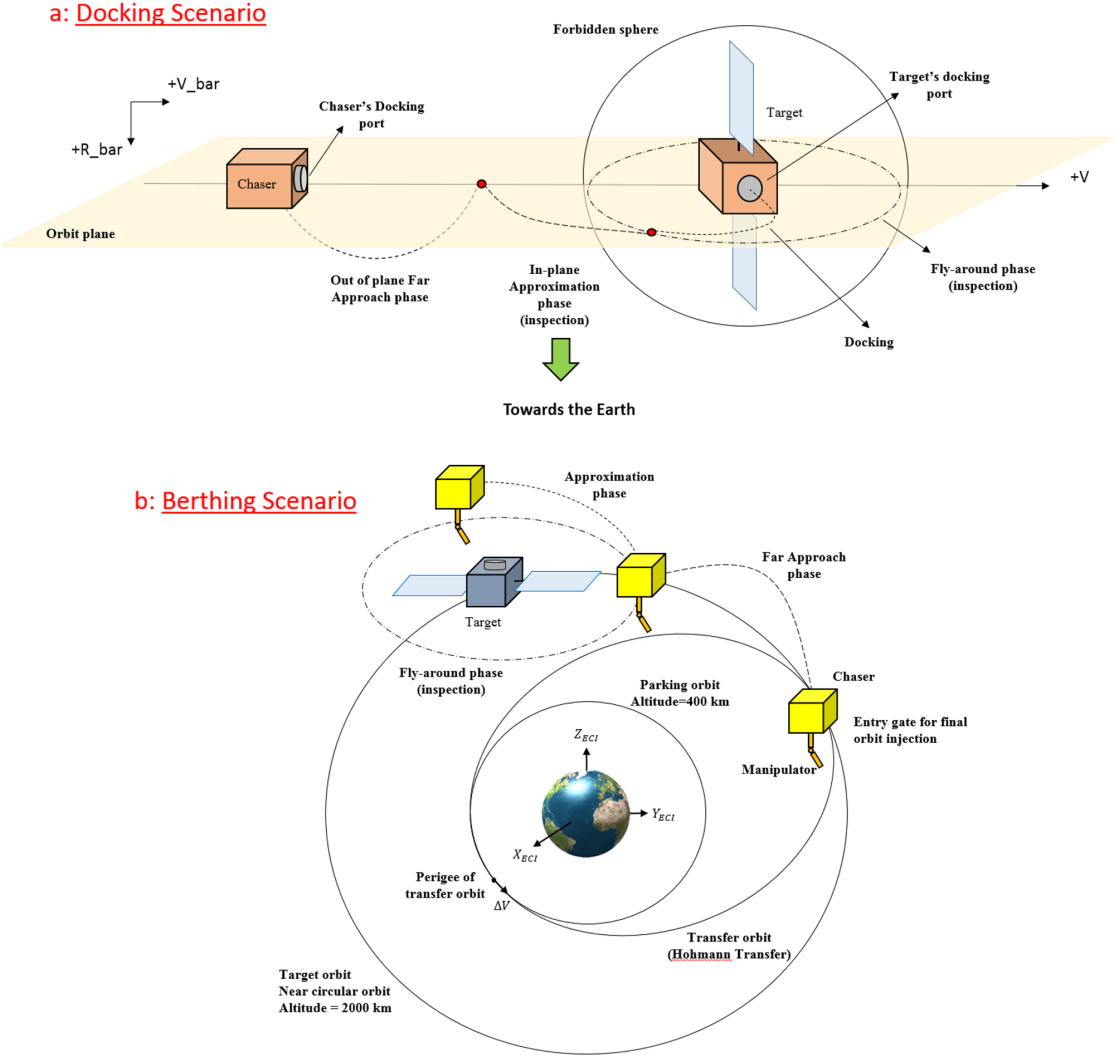
Schematic of docking and birthing concepts.

There are two (1) berthing and (2) docking options for the final physical connection (mating). In the berthing case, the connection is performed by a robotic manipulator/arm that captures the target’s structure and reduces the relative distance (as shown in Fig. 1b)^13^. Canadarm-1 and Canadarm-2 are the experimental successful models in this field. The probability of hard and destructive physical contact between the robotic arm and the target’s structure increases throughout the capturing phase. Although some researchers are focused on impedance control techniques to handle this problem^14,15^, degradation of joints’ lubricant, time-varying friction, and backlash/slack are really hard to deal with. On the contrary, docking is a more cost-effective, simple, and safe option for on-orbit servicing missions, especially in the face of a tumbling target. Space docking has a research history of about 55 years (longer history than berthing)^16^. In the docking operation case, the chaser closes to the target and connects through the docking port (as shown in Fig. 1a)^17^. As a real-world example, the first manual docking operation occurred during the Gemini-VIII project (early 1966)^18^, and the first autonomous docking operation occurred a few years later during the Cosmos spacecraft program (in 1967)^19^. In those days, mastering docking was a vital step for success in both (1) future moon landings and (2) forming space station missions. As a result, the issue of rendezvous and docking has caught the interest of several scholars from all over the world as a challenging and interesting topic.

Despite the long-range approach phase where only relative distance control is considered, both the relative attitude and position (pose) motion should be controlled in the close-range approach^20^, simultaneously. It is quite understandable that accuracy, agility, and robustness in the final approaching phase would be a much bigger concern in comparison with the rendezvous phase.

The conventional linear quadratic regulator (LQR) together with the linear state feedback (PD type) controller is addressed by^11^ to reduce the relative pose motion. Although the proposed approach can be good enough to encounter cooperative and stable targets. Due to the coupling and high nonlinearity of the relative pose equations of motion, it is challenging to guarantee mission safety and collision avoidance with sufficient precision and robustness^21^. Also, it should not be forgotten that in practice, we would face more difficulties in confronting autonomous guidance, navigation, and control (GNC) systems^22^, and some sophisticated hardware or software limitations^23^. Consequently, a compulsory requirement for the chaser spacecraft is the ability to deal with parametric and modeling uncertainties, external disturbances, actuator misalignment, under-actuation, control input uncertainty, delay, and measurement errors^18^. In this case, conventional/classical smooth control laws are no longer useful, especially when agile and large-angle maneuvers are needed. Therefore, various nonlinear control laws were studied by researchers.

Model predictive control (MPC) is one of only a few advanced control methods that has found success in industrial control applications. It meets our expectations for optimal control while responding much better to disturbances and delays caused by the control law's update in each step time. MPC has a powerful tool to control high-order multivariable constrained systems. But designing predictive control for nonlinear systems will come with some sophistication. In this field, an MPC approach is being developed by^24^ for an autonomous servicer spacecraft approaching a tumbling target at an ultra-close distance. The target was considered to behave in a deterministic manner. Cairano^25^ used the MPC method for the in-orbit plane proximity problems, taking into account the collision avoidance condition. Li^26^ applied the MPC method on a servicing spacecraft to maximize control accuracy and smoothness to avoid an unexpected change or overshoot of trajectory. Mammarella^27^ introduced a tube-based MPC to guarantee the required accuracy in the presence of persistent disturbance. A state-dependent MPC has been presented by^28^ for proximity operation near a non-cooperative spacecraft in an elliptical orbit. Malladi^29^ developed a nonlinear model predictive control (NMPC) for controlling a free-floating rigid spacecraft equipped with 8 gimbaled thrusters and a robotic manipulator in rendezvous with a tumbling asteroid. MPC, on the other hand, is not only a time-consuming approach (especially for long prediction horizons), but it also increases the computational cost, which may not be ideal for the real-time agile maneuvers required in the close approach and docking with a tumbling target. Additionally, the MPC is very susceptible to model uncertainties and model correctness (non-modeled dynamics).

Robust control theory presents a set of methods to handle various types of uncertainties and disturbances. Robust design techniques result in fixed structures/compensators that perform acceptable performance over a specified/bounded range of uncertainties or disturbances. Thus, robust control approaches have been studied to handle the model uncertainty, input saturation, and external disturbances in the field of rendezvous problem^30–32^. Gu^33^ designed the robust parametric control for autonomous rendezvous under inertia uncertainty. Based on the C-W equations, a robust ${H}_{\infty}$ state-feedback controller is designed to deal with external disturbances^34^. A genetic algorithm (GA) based robust controller has been investigated by^35^ for the cooperative rendezvous problem. Pirat^36^ improved ${H}_{\infty}$ based robust controller using $\mu -\mathrm{synthesis}$ for 2 nanosatellites rendezvous problem. But, The proposed controller was derived based on linearized 6DOF motion. Andrade^37^ presented a ${H}_{\infty}$ based controller to compensate for the non-modeled space environmental effects. To make the region of attraction as large as possible, Wang^38^ has proposed a robust control for the spacecraft rendezvous by considering the parameter uncertainties and actuator unsymmetrical saturation. It is notable that mentioned references only considered the robustness of the controller against actuator saturation, model uncertainties, and external disturbances. Indeed, high control accuracy, minimum control effort, output smoothness, and good convergence performance were ignored.

In addition to having strong robustness against disturbances, adaptive control techniques also show good flexibility when faced with unexpected/sudden parameter changes and well performance in the transition state. Adaptive control schemes extract knowledge from the system dynamics using identification methods to redesign the control law. Perhaps one of the main advantages of adaptive control over robust control is that it does not need to know the range of uncertainty^39^. This characteristic is advantageous because it is impossible to exactly forecast the servicing spacecraft's operational condition and environment. An adaptive backstepping controller is proposed by Sun^40^ to deal with input saturation in rendezvous with a tumbling target. A command filter-based adaptive backstepping controller is derived by Zhang^41^ for close-range rendezvous problems to overcome “explosion of terms”, but The number of adaptive parameters is immeasurably large. Sun^42^ combined the classical backstepping technique with a simple norm-estimation adaptive method to improve the controller’s performance, but 12 unknown parameters should be estimated online. Shao^43^ addressed a novel adaptive PPC framework for spacecraft proximity operations with a tumbling target considering motion and performance constraints. An immersion and invariance (I&I) adaptive pose control scheme based on artificial potential functions (APFs) is suggested by Shao^44^ for six-degree-of-freedom pose control with docking port line-of-sight consideration. very high control effort, which leads to unreasonable fuel consumption is the main drawback of this method. Excellent work was done by Liang Sun^45^, who developed a saturated adaptive fault-tolerant relative pose controller by considering constrained relative states for space proximity missions. But this approach is somehow difficult to use because there are too many controller parameters to tune. The two general barriers to the reviewed adaptive methods are as follows: first) some adaptive control techniques, such as model reference adaptive control (MRAC) and its extensions, including L1 adaptive control and simple adaptive control are difficult to extend to adapt to state constraints. Second) other solutions may adhere to the classical certainty equivalence (CE) principle and call for a "realizability" requirement, which does not hold in the Lyapunov sense when taking into account dynamic restrictions.

Because of the critical role fuel consumption plays in rendezvous and docking success, potential improvements based on optimal control theory are still under investigation. A feed-forward time/fuel-consumption optimal control has been studied by Ma^46^ for rendezvous with a cooperative and stable target, under small relative velocity, zero external disturbances, and the absence of uncertainty. An optimal rendezvous trajectory both for minimum-time and minimum-energy cases has been designed by Boyarko^47^ based on the linearized equations of motion. The state-dependent Riccati equation (SDRE) is similar to the LQR method with the only difference that it solves the Riccati equation in each time sample to calculate the optimal gain. SDRE is known as a simple controller that can be used efficiently for non-linear systems. The error caused by the linearization of the equations of motion (by the Jacobian method) is not injected into this method. Stansbery^48^ used the suboptimal SDRE (first presented by Krcislclmeisr^49^) to control the coupled ideal pose equation of motion introduced by Terui^50^. Inspired by Stansbery^48^, far-range rendezvous and final proximity phase in an elliptical orbit have been investigated by Navabi^10^. The resulting state-dependent algebraic Riccati equation is solved based on the eigenvectors of a Hamiltonian matrix (a direct method of solving SDRE using the Schur decomposition algorithm that was proposed by Krcislclmeisr^49^) in order to find nonlinear optimal control gains. One of the main issues in practice is how much knowledge about a system is needed in order to design a control law. Based on this consideration, the method proposed in^10^ is useful when comprehensive and complete information about the system is available. Xin^51^ designed the $\theta -D$ nonlinear optimal control for a flexible chaser spacecraft to rendezvous with a cooperative target while minimizing the vibration of the flexible parts. However, the SDRE is an iterative method that requires solving the Riccati equation repetitively at every step. Thus, it may not be the best option for high-order systems, especially from the real-time calculation point of view. A novel and interesting solution for the rendezvous and docking problem has been presented by Subbarao^52^ Based on a novel “virtual target” construction. The designed controller ensures that the relative position vector decreases while is always directed toward the docking port of the target, but the simulation results show the weak accuracy and high fluctuation of this method. Relying on C-W equations, an output tracking scheme is considered by Lee^53^ for achieving successful disturbance rejection. But studying full nonlinear 6DOF motion dynamics including the actuators' role could have yielded further interesting results.

The sliding mode control (SMC) law is known as an efficient tradeoff between robustness, flexibility, accuracy, and simplicity that can handle the coupled nonlinear 6 DOF relative equations of motion, effectively. Perhaps Terui^50^ can be cited as a groundbreaking researcher who proposed SMC for rendezvous problems, but under idealized conditions (zero disturbance and uncertainty). Both chaser and target spacecraft are inevitably subject to unknown disturbances. Along with the external disturbances, there also exist some uncertainties that have much to do with the mission’s reliability and quality level. A sliding-mode-based robust adaptive controller is designed during rendezvous with a non-cooperative target by Sun^54^ to deal with parametric uncertainty (in mass and moment of inertia) and external disturbance. To deal with the formation flying mission in the presence of mass uncertainty and bounded external disturbance, an inverse optimal control law is designed based on (1) the Sontag type formula and (2) the control Lyapunov function by Pukdeboon^55^. Then a robust inverse optimal position and attitude controller is designed using a new second-order integral sliding mode control method that combines a sliding mode control with the derived inverse optimal control. Ye^56^ and Abdollahzadeh^17^ discussed the issue of guidance, during the rendezvous. Ye^56^ proposed a compound controller based on SMC law which is able to guarantee that the docking port of servicing spacecraft is always directed towards the docking port of the tumbling target, in the presence of disturbances. Abdollahzadeh^17^ developed the work done by Ye^56^, and Boyarko^57^. In this work, two conventional sliding mode controllers have been developed to manage the relative pose motion of the chaser and tumbling target spacecraft while taking into account the line-of-sight (LOS) between the two vehicles’ docking ports^17^. All previous studies relied on visual navigation, and the issue of control in the absence of some relative navigation information deserves further investigation. A new output feedback controller for coupled pose motion that doesn’t require translational and angular velocity measurements is presented by Pukdeboon^21^. In this regard, a new sliding-mode-based integral filter (which was first introduced by Li^48^) is developed to estimate the first-time derivatives of attitude and position in a finite time. Then, the estimated derivative values are used in the controller, instead of the measured translational and angular velocity variables. Due to strict constraints on the chaser's fuel mass, minimizing fuel consumption is vital to meeting the rendezvous objectives. In this regard, a combination of the second-order SMC (for the sake of robustness) and minimal time three-level (bang-bang) control (for the sake of fuel consumption) technique has been presented by Tournes^58^. For the deep-space proximity operations, Sun^59^ designed a trajectory to decrease the control efforts (which results in a decrease in fuel usage) of the chaser spacecraft. Then a novel robust adaptive controller based on the SMC technique is designed for trajectory tracking. To solve the chattering problem that is the primary issue with the SMC method, the second-order sliding mode algorithm based on the super twisting method is used by Chen^60^ for rendezvous and docking with tumbling non-cooperative target spacecraft. The SMC approaches developed in this work suffered from an annoying singularity problem. The chattering-free full-order recursive sliding mode control is proposed by Song^61^. The main disadvantage of this approach is that the higher-order sliding surface brings difficulties in the implementation phase. A globally asymptotically stable chattering-free SMC is designed by Xu^62^. Considering agility to be one of the most crucial needs of on-orbit maintenance missions, a terminal sliding mode technique is used by Zhang^63^ to track the integrated position and attitude command, in a pre-determined finite time. In real-world conditions, actuator saturation is a common and important issue that can have a significant impact on stability. Zhang published his second paper in the field of coupled relative pose control in 2012 and supplemented the previous research^64^. In this paper, an adaptive back-stepping controller was designed to cope with control input saturation. A bit later, he published a similar paper, expanding her previous research to include mass uncertainty in the controller design^65^. In light of the benefits of discrete controllers from an implementation perspective, two discrete-time SMCs are developed by Lincoln^66^ to provide robust 6 DOF control of a rigid holonomic spacecraft. One of the proposed controllers is implemented on a 5 DOF hardware in the loop (HIL) testbed. Unfortunately, the discretization procedure resulted in the linearization of the equations of motion. Recently, some researchers have focused on finite-time stability control strategies. an adaptive sliding mode disturbance observer, and a finite-time SMC in terms of a dual quaternion are designed by Zhu^67^. But, the proposed controller was affected by the singularity problem. On this basis, Zheng^68^ proposed a non-singular terminal SMC that can eliminate the singularity problem. But, the controller required prior knowledge of each relative motion parameter. A finite-time non-singular terminal SMC technique is presented by Wu^69^ to achieve 6 DOF maneuvers. Next, a modified controller based on a finite-time observer is designed to deal with parametric uncertainties and disturbances, which also is effective in chattering reduction^69^. An adaptive fixed-time control scheme with a novel sliding manifold is employed to accomplish the 6-DOF rendezvous (close-range) and docking mission for the servicing spacecraft^70^. But, the controller was very susceptible to the initial condition. The topic of automaticity has received special attention from several scholars. The problem of uncertain autonomous spacecraft rendezvous has been investigated using a disturbance observer and nonsingular fast terminal sliding mode control (NFTSMC) by Wang^71^. Safe and reliable control in the case of actuator failure has been an active research topic. Time-varying SMC has been developed by Hu^72^ for capturing a tumbling target, autonomously. The controller is able to accommodate a large class of actuator faults. The relative rotational motion equation does not contain the RWs term (angular momentum or angular rate). Imani^73^ designed a backstepping sliding mode controller that shows robustness against actuator (only thruster) degradation and failure for a short period of time.

To make a long story short, previous studies have had common weaknesses in at least one of the following aspects:

- First of all, coupled full nonlinear relative attitude and translational motion control has not received enough attention as it deserves. For instance, the majority of studies were founded on the C-W or H-C-W equations of motion (such as^17,71,73^). While C-W or H-C-W are simplified and linearized equations that are only applicable for rendezvous with a target orbiting in a near-circular orbit. At the final approach phase of the rendezvous, the C-W model will be invalid due to the kinematic coupling of rotational and transnational motion^74^.
- Secondly, no discussion is made about the chaser spacecraft’s attitude control actuators (Specifically reaction wheels) role in the relative attitude equation of motion, despite being of great theoretical and practical interest(such as^21,54,59,62,72,73^).
- Most importantly, the uncertainty of control input and measurement error has never been considered in any research so far.
- And fourth, the environmental disturbances modeling that especially affects the performance level of the docking/berthing phase has been neglected (such as^50^).
- Some proposed controllers show great performance only in specific initial conditions (such as^54,70^).
- The last aspect is that some of the proposed controllers are not a practical option due to their high computational load and time-consuming. because, there are serious restrictions on both (1) spacecraft’s onboard computer (OBC) power consumption, and (2) onboard computational resources^75^. Thus, control laws based on iterative methods, huge observer/ identification algorithms, heavy prediction models, or complex adaptation laws are not suitable cases from practical point of view.

On the contrary, we tried to compensate for all of the mentioned weaknesses. The main goal of this paper is to develop a controller for a full nonlinear relative pose motion, which performs (1) enough accuracy in the presence of actuators’ misalignment and saturation and (2) shows acceptable robustness against the different disturbances (gravity gradient, solar radiation pressure, atmosphere drag, and magnetic moment) and uncertainties(mass/inertia, control input, measurement,) sources. The designed controller is free from any iteration, estimation or identification, optimization or time-consuming logical loop. The chaser spacecraft benefits 3 orthogonal reaction wheels, and 6 reaction thrusters for attitude and translational maneuvers, respectively. In this regard, the reaction wheel's term and thrusters' adverse torque are incorporated in the relative attitude motion. Finally, the sliding mode control method has been used to handle the derived equations, as a trade-off between accuracy, robustness, and computational cost.

The rest of this work is organized as follows: In the upcoming section, relative pose motion and disturbance torque/force models are given after the definition of the coordinate systems. The controller design is presented in the section “Controller design”. Simulation results are investigated in section “Simulations and results analysis”, and the paper ends with the conclusion discussion in section “Conclusion”.

## Problem formulation

### An introduction to coordinate systems

It is necessary to define some coordinate systems to describe the relative motion of the chaser (C) and the target (T) spacecraft, which are defined as follows:

1. Earth-centered inertial (ECI) coordinate system: The ECI coordinate system is denoted as ${O}_{{x}_{I}{y}_{I}{z}_{I}}$ and its origin is fixed to the center of the Earth. The ${x}_{I}$ axis points toward the vernal equinox ($\Upsilon$), the ${z}_{I}$ axis is normal to the equatorial plane and points toward the North, the ${y}_{I}$ axis completes the right-hand rule.
2. Local-Vertical Local-Horizontal (LVLH) coordinate system: The LVLH coordinate system is denoted as ${O}_{{x}_{L}{y}_{L}{z}_{L}}$ and its origin is fixed to the mass center of the target spacecraft. The ${x}_{L}$ axis is directed along the radius vector of the target’s center of mass from the Earth’s center, the ${z}_{L}$ axis is normal to the orbital plane, and the ${y}_{L}$ axis completes the right-handed coordinate system. in a circular orbit, ${y}_{L}$ is in the direction of the orbital velocity vector.
3. Reference Coordinate system: The chaser reference coordinate system (CR) and target reference coordinate system (TR) are denoted as ${O}_{{x}_{CR}{y}_{CR}{z}_{CR}}$ and ${O}_{{x}_{TR}{y}_{TR}{z}_{TR}}$, respectively. Their origin is fixed to the mass center of the spacecraft. the ${z}_{CR}$ and ${z}_{TR}$ are points toward the center of the Earth, ${x}_{CR}$ and ${x}_{TR}$ are in the orbit plane, in the direction of the chaser velocity vector. ${y}_{CR}$ and ${y}_{TR}$ are normal to the local plane of the orbit and complete a right-hand triad.

Note: LVLH, TR, and CR coordinate systems are moving with the center of mass of the spacecraft in the orbit but don’t rotate.

**Figure 2:**
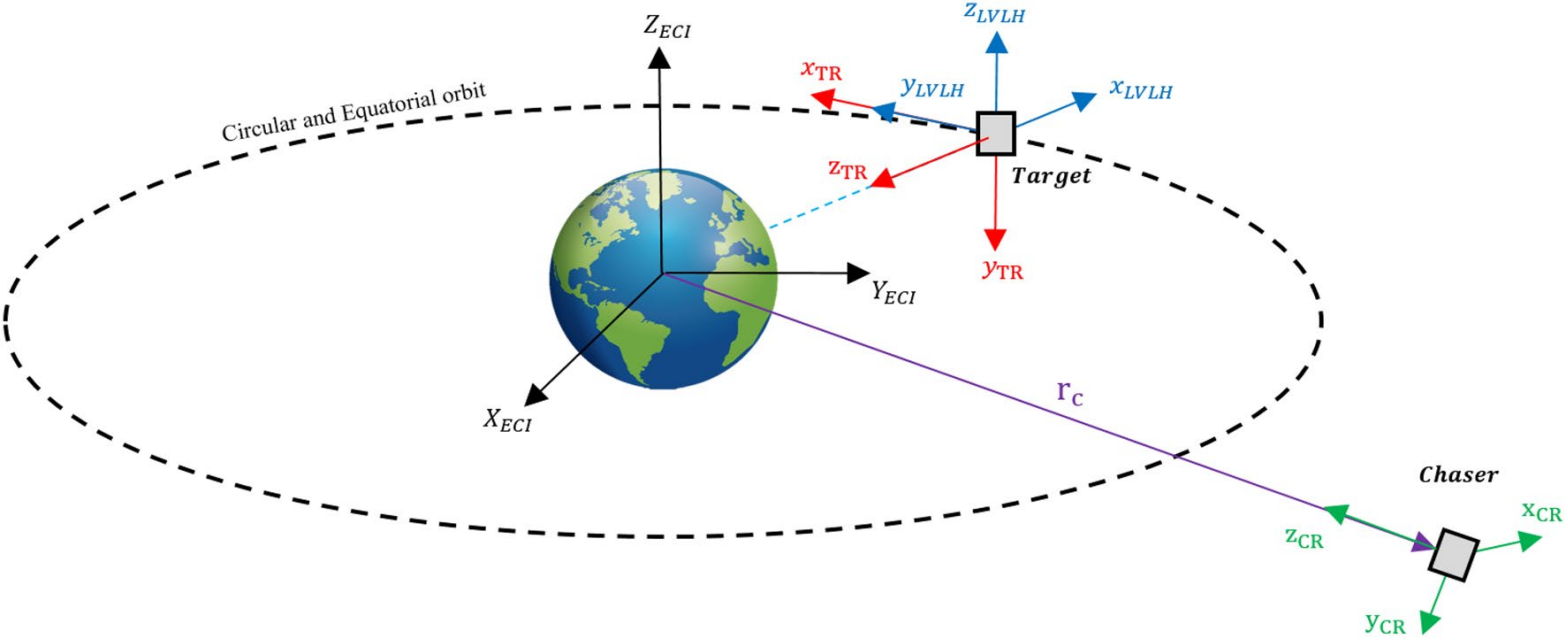
Schematic Definition of LVLH, TR, and CR coordinate systems.

Figure 2 illustrates the LVLH and reference coordinate systems:

4. Body coordinate system: The body coordinate system of the chaser (C) and target spacecraft (T) are denoted as ${O}_{{x}_{c}{y}_{c}{z}_{c}}$ and ${O}_{{x}_{t}{y}_{t}{z}_{t}}$, respectively. The origin of ${O}_{{x}_{c}{y}_{c}{z}_{c}}$ and ${O}_{{x}_{t}{y}_{t}{z}_{t}}$ coordinate systems are attached to the center of mass of the chaser and target spacecraft. ${x}_{c}$ and ${x}_{t}$ are Coincident with the vehicle's longitudinal axis, positive forward. ${y}_{c}$ and ${y}_{t}$ are coincident with the vehicle's lateral axis, positive to the right. ${z}_{c}$ and ${z}_{t}$ are coincident with the vehicle's normal axis, and direction to form the right-hand triad.

${x}_{c}$ axis of the chaser spacecraft and ${-z}_{t}$ axis of target spacecraft are outward normal direction of the docking ports.

**Figure 3:**
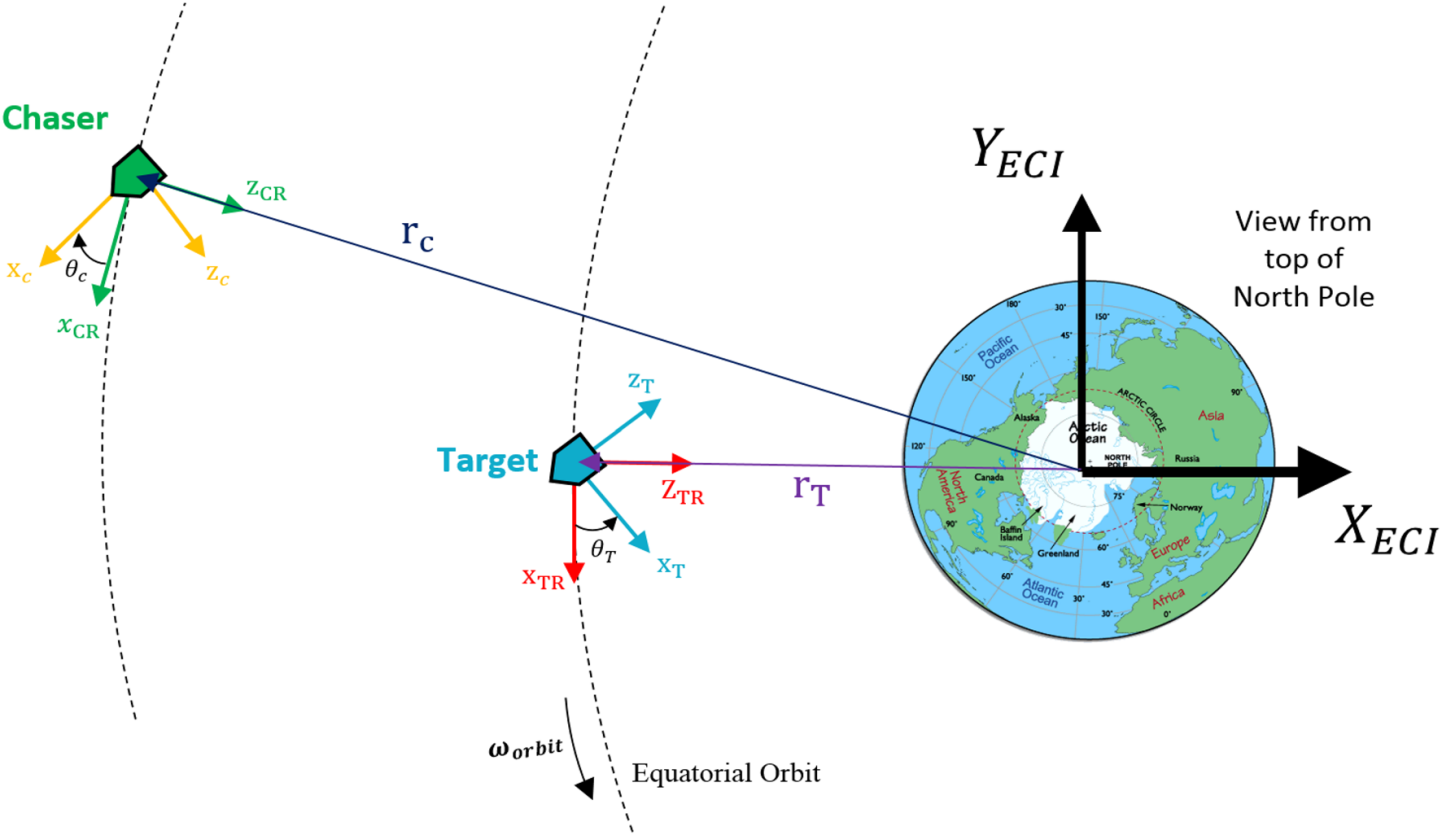
Schematic Definition of coordinate systems.

**Figure 4:**
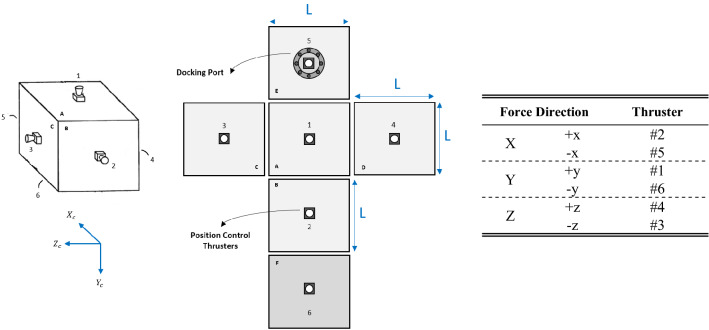
Position control thrusters of the chaser spacecraft.

Figure 3 illustrates the body coordinate systems from the top view:

According to what is schematically shown in Fig. 4, it is assumed that the chaser satellite uses 6 thrusters for position control. Each face is equipped with a thruster and the thrust vector passes through the center of gravity so that does not produce torque. Therefore, the 3-axis translational motion of the chaser spacecraft is controllable, independently. The docking port is located on the E side, along with the ${x}_{c}$.

**Figure 5:**
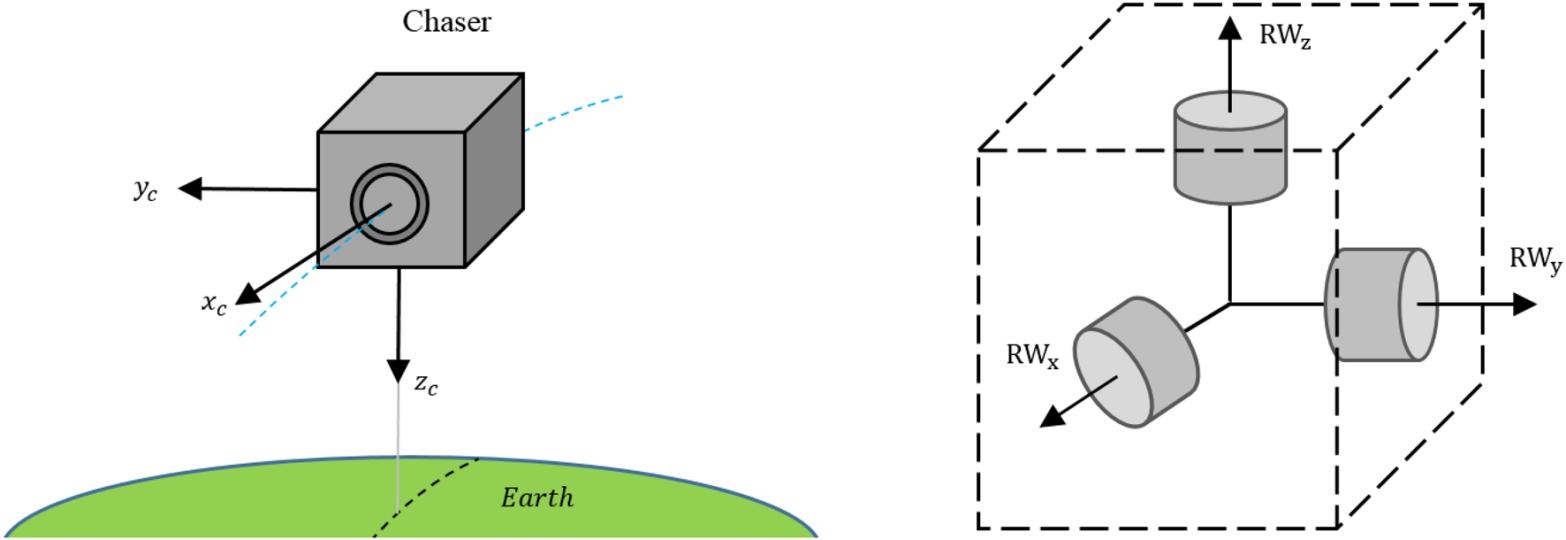
Reaction wheels of the chaser spacecraft.

Reaction wheels are accurate, reliable, and cost-effective actuators that are suitable for precise attitude control of the chaser spacecraft in final approximation. As shown in Fig. 5, the chaser spacecraft uses three orthogonal reaction wheels mounted on the principal body axes for attitude control.

### Rotational dynamics

#### Rigid spacecraft attitude equation of motion

Reaction wheels are accurate, reliable, and cost-effective control actuators that are used greatly in High-Tec space missions due to their ability in providing high pointing accuracy (up to ${0.1}^{^\circ}$) and precision (up to $0.001\;^\circ/s$). The use of reaction wheels ensures a smooth/soft and safe connection during the ducking process^76^. Thus, assuming the chaser spacecraft is a rigid body equipped with 3 orthogonal reaction wheels, its attitude dynamic equation can be defined by the Euler equation^77^:

1 $${I}_{c}{\dot{\omega}}_{c}=-S\left({\omega}_{c}\right){I}_{c}{\omega}_{c}-S\left({\omega}_{c}\right){h}_{RW}+{T}_{c}+{T}_{{d}_{c}}+{T}_{{d}_{TH}}$$

where ${\overrightarrow{\omega}}_{c}={\left[{\omega}_{{c}_{x}},{\omega}_{{c}_{y}},{\omega}_{{c}_{z}}\right]}^{T} \,\,\epsilon\,\, {R}^{3\times 1}$ denotes the angular velocity vector of the chaser spacecraft with respect to the ECI coordinate system, expressed in the chaser body coordinate system. ${\overrightarrow{T}}_{c}={\left[{T}_{{c}_{x}},{T}_{{c}_{y}},{T}_{{c}_{z}}\right]}^{T} \,\,\epsilon\,\, {R}^{3\times 1}$ represents an overall control torque vector produced by control actuators. ${\overrightarrow{T}}_{{d}_{c}}={\left[{T}_{{d}_{cx}},{T}_{{d}_{cy}},{T}_{{d}_{cz}}\right]}^{T} \,\,\epsilon\,\, {R}^{3\times 1}$, ${\overrightarrow{T}}_{{d}_{TH}}={\left[{T}_{{d}_{{Th}_{x}}}, {T}_{{d}_{{Th}_{y}}}, {T}_{{d}_{{Th}_{z}}}\right]}^{T} \,\,\epsilon\,\, {R}^{3\times 1}$ Indicate the external and thrusters disturbance torque (see section “Relative translational motion control”) vector. ${\overrightarrow{h}}_{\mathrm{RW}}={\left[{h}_{{RW}_{x}},{h}_{{RW}_{y}},{h}_{{RW}_{z}}\right]}^{T} \,\,\epsilon\,\, {R}^{3\times 1}$ represents the angular momentum vector of reaction wheels. In the case of using reaction wheels, the control torque is equal to the time derivative of the wheels’ angular momentum $\left({\overrightarrow{T}}_{c}=-{\dot{h}}_{RW}\right)$. $S\left(z\right)$ is the skew-symmetric matrix of the $z$ vector which is defined as follows:

2 $$S\left(z\right)=\left[\begin{array}{ccc}0& -{z}_{z}& {z}_{y}\\ {z}_{z}& 0& -{z}_{x}\\ -{z}_{y}& {z}_{x}& 0\end{array}\right]$$

$S\left(z\right)$ satisfies $\Vert S\left(z\right)\Vert =\Vert Z\Vert$, ${Z}^{T}S\left(z\right)=0$, ${X}^{T}S\left(z\right)X=0$, and $S\left(z\right)X=-S\left(X\right)z$ for any $X\,\,\epsilon\,\, {R}^{3\times 1}$.

${I}_{c} \,\,\epsilon\,\, {R}^{3\times 3}$ denotes the moment of inertia tensor of the chaser spacecraft, which is given as:

3 $${I}_{c}=\left[\begin{array}{ccc}{I}_{{c}_{xx}}& {I}_{{c}_{xy}}& {I}_{{c}_{xz}}\\ {I}_{{c}_{yx}}& {I}_{{c}_{yy}}& {I}_{{c}_{yz}}\\ {I}_{{c}_{zx}}& {I}_{{c}_{zy}}& {I}_{{c}_{zz}}\end{array}\right]$$

Quaternion parameters are an optimal approach for kinematics modeling due to non-singularity and onboard computation simplicity. Thus, the quaternion parameters are used to develop the attitude kinematics model of the chaser spacecraft as follows:

4 $${\dot{q}}_{c}=\frac{1}{2}Q\left({\omega}_{c}\right){q}_{c}$$

where $${\overrightarrow{q}}_{c}={\left[\begin{array}{cc}{q}_{{c}_{v}}^{T}& {q}_{{c}_{4}}\end{array}\right]}^{T} \,\,\epsilon\,\, {\mathfrak{R}}^{4\times 1}$$ is the attitude quaternion. ${q}_{{c}_{4}}$ and ${\overrightarrow{q}}_{{c}_{v}}={\left[{q}_{{c}_{1}}, {q}_{{c}_{2}}, {q}_{{c}_{3}}\right]}^{T} \,\,\epsilon\,\, {\mathfrak{R}}^{3\times 1}$ are the scaler part and vector component of the ${q}_{c}$, respectively. $Q\left({\omega}_{c}\right) \,\,\epsilon\,\, {\mathfrak{R}}^{4\times 4}$ is defined as follows^77^:

5 $$Q\left({\omega}_{c}\right)=\left[\begin{array}{cc}0& -{\omega}_{c}^{T}\\ {\omega}_{c}& -S\left({\omega}_{c}\right)\end{array}\right]$$

Without loss of generality, the attitude dynamics and kinematics equations of the target spacecraft are given by:

6 $${I}_{T}{\dot{\omega}}_{T}=-S\left({\omega}_{T}\right){I}_{T}{\omega}_{T}+{T}_{{d}_{T}}$$

where ${\overrightarrow{\omega}}_{T}={\left[{\omega}_{{T}_{x}},{\omega}_{{T}_{y}},{\omega}_{{T}_{z}}\right]}^{T} \,\,\epsilon\,\, {\mathfrak{R}}^{3\times 1}$ represents the angular velocity vector of the target spacecraft with respect to the ECI coordinate system, expressed in the target body coordinate system. ${\overrightarrow{T}}_{{d}_{T}}={\left[{T}_{{d}_{Tx}},{T}_{{d}_{Ty}},{T}_{{d}_{Tz}}\right]}^{T} \,\,\epsilon\,\, {\mathfrak{R}}^{3\times 1}$ Indicates the space environment disturbances torque vector acting on the target spacecraft. ${I}_{T} \,\,\epsilon\,\, {\mathfrak{R}}^{3\times 3}$ denotes the moment of inertia tensor of the target spacecraft.

As is clear from Eq. (6) we assumed that the target spacecraft lost control and is tumbling with a constant angular rate. The target spacecraft is assumed to be alive and cooperative. In another word, the chaser is aware of the angular velocity and attitude information of the target satellite.

Similarly, the attitude kinematics of the target spacecraft is given below:

7 $${\dot{q}}_{T}=\frac{1}{2}Q\left({\omega}_{T}\right){q}_{T}$$

$${\overrightarrow{q}}_{T}={\left[\begin{array}{cc}{q}_{{T}_{v}}^{T}& {q}_{{T}_{4}}\end{array}\right]}^{T} \,\,\epsilon\,\, {\mathfrak{R}}^{4\times 1}$$ is the attitude quaternion of the target spacecraft. ${q}_{{T}_{4}}$ and ${\overrightarrow{q}}_{{T}_{v}}={\left[{q}_{{T}_{1}}, {q}_{{T}_{2}}, {q}_{{T}_{3}}\right]}^{T} \,\,\epsilon\,\, {\mathfrak{R}}^{3\times 1}$ are the scaler part and vector component of the ${q}_{T}$, respectively. $Q\left({\omega}_{T}\right) \,\,\epsilon\,\, {\mathfrak{R}}^{4\times 4}$ is defined as follows:

8 $$Q\left({\omega}_{T}\right)=\left[\begin{array}{cc}0& -{\omega}_{T}^{T}\\ {\omega}_{T}& -S\left({\omega}_{T}\right)\end{array}\right]$$

#### Relative attitude motion

In relative attitude dynamics, both spacecraft are treated as rigid bodies. It is worth noting that both the relative translational dynamics (Eq. (34)) and the relative attitude dynamics will be expressed in the chaser spacecraft body coordinated system^56^.

To synchronize the two vehicles’ angular velocity and also point the chaser's docking port toward the docking port of the target, ${x}_{c}$ axis is required to point toward the direction of the ${z}_{t}$ (see Fig. 3). To this end, a virtual target can be assumed to track, which has a body frame $\left({O}_{{x}_{d}{y}_{d}{z}_{d}}\right)$ fixed on the actual target and its longitudinal axis $\left({x}_{d}\right)$ is aligned with the direction of the actual target’s ${z}_{T}$ axis. The virtual body frame can be obtained by rotating the actual target body coordinate system by ${-90}^{^\circ}$ about the ${y}_{t}$. Thus, the rotation matrix from the actual target frame (${O}_{{x}_{t}{y}_{t}{z}_{t}}$) to the virtual target frame can be represented by the following direct cosine matrix:

9 $${R}_{T}^{d}=\left[\begin{array}{ccc}\mathrm{cos}\left({-90}^{^\circ}\right)& 0& -\mathrm{sin}\left({-90}^{^\circ}\right)\\ 0& 1& 0\\ \mathrm{sin}\left({-90}^{^\circ}\right)& 0& \mathrm{cos}\left({-90}^{^\circ}\right)\end{array}\right]=\left[\begin{array}{ccc}0& 0& 1\\ 0& 1& 0\\ -1& 0& 0\end{array}\right]$$

Clearly, the quaternion corresponding to this rotation can be written as: ${{\overrightarrow{q}}_{Td}=\left[{{q}_{Td}}_{1}, {{q}_{Td}}_{2}, {{q}_{Td}}_{3}, {{q}_{Td}}_{4}\right]}^{T}={\left[0.7071, 0, -0.7071, 0\right]}^{T}$. Also, the angular velocity of the actual target expressed in the virtual target frame that should be tracked by the chaser becomes:

10 $${\omega}_{d}={R}_{T}^{d}{\omega}_{T}={\left[{\omega}_{{T}_{z}}, {{\omega}_{T}}_{y}, {{-\omega}_{T}}_{x}\right]}^{T}$$

${\overrightarrow{\omega}}_{d}={\left[{\omega}_{{d}_{x}}, {{\omega}_{d}}_{y}, {{-\omega}_{d}}_{z}\right]}^{T} \,\,\epsilon\,\, {\mathfrak{R}}^{3\times 1}$ denotes the desired (virtual target’s) angular velocity that should be tracked.

It should be noted that the ${R}_{T}^{d}$ is a time constant matrix and ${\dot{\omega}}_{d}={R}_{T}^{d}{\dot{\omega}}_{T}$.

Similarly, the desired quaternion for the chaser to track becomes^78^:

11 $${q}_{d}={q}_{T}\otimes{q}_{Td}$$

$${q}_{d}=\left[\begin{array}{c}{q}_{{T}_{4}}\\ {q}_{{T}_{1}}\\ {q}_{{T}_{2}}\\ {q}_{{T}_{3}}\end{array} \begin{array}{c}-{q}_{{T}_{1}}\\ {q}_{{T}_{4}}\\ {q}_{{T}_{3}}\\ -{q}_{{T}_{2}}\end{array} \begin{array}{c}-{q}_{{T}_{2}}\\ -{q}_{{T}_{3}}\\ {q}_{{T}_{4}}\\ {q}_{{T}_{1}}\end{array} \begin{array}{c}-{q}_{{T}_{3}}\\ {q}_{{T}_{2}}\\ -{q}_{{T}_{1}}\\ {q}_{{T}_{4}}\end{array}\right]\left[\begin{array}{c}0.7071\\ 0\\ -0.7071\\ 0\end{array}\right]=\left[\begin{array}{c}0.7071{q}_{{T}_{4}}+0.7071{q}_{{T}_{2}}\\ 0.7071{q}_{{T}_{1}}+0.7071{q}_{{T}_{3}}\\ 0.7071{q}_{{T}_{2}}-0.7071{q}_{{T}_{4}}\\ 0.7071{q}_{{T}_{3}}-0.7071{q}_{{T}_{1}}\end{array}\right]$$

Now, attitude synchronization reduces to the problem of tracking the virtual target’s attitude (${q}_{d}$) and its angular velocity (${\omega}_{d}$).

The relative/error angular velocity vector expressed in the chaser coordinate system is:

12 $${\omega}_{rel}={\omega}_{c}-{R}_{d}^{c}{\omega}_{d} \Rightarrow {\omega}_{rel}={\omega}_{c}-{R}_{T}^{c}{\omega}_{T}$$

where ${\overrightarrow{\omega}}_{rel}={\left[{\omega}_{{rel}_{x}}, {\omega}_{{rel}_{y}}, {\omega}_{{rel}_{z}}\right]}^{T} \,\,\epsilon\,\, {\mathfrak{R}}^{3\times 1}$ represents the relative angular velocity vector of the chaser body coordinate system $\left({O}_{{x}_{c}{y}_{c}{z}_{c}}\right)$ with respect to the virtual target body coordinate system $\left({O}_{{x}_{d}{y}_{d}{z}_{d}}\right)$, or target body $\left({O}_{{x}_{T}{y}_{T}{z}_{T}}\right)$. ${R}_{d}^{c} \; \,\,\epsilon\,\, \; {\mathfrak{R}}^{3\times 3}$ is the rotation matrix from the virtual body coordinate system to the chaser body coordinate system. ${R}_{T}^{c}$ is the rotation matrix from the target body coordinate system to the chaser body coordinate system that is defined as:

13 $${R}_{T}^{c}={R}_{d}^{c}{R}_{T}^{d}$$

by calculating the time derivative of Eq. (12), the relative attitude dynamics $\left({\dot{\omega}}_{rel}\right)$ is obtained as follows^56^:

14 $${\dot{\omega}}_{rel}={\dot{\omega}}_{c}+{\omega}_{rel}\times {R}_{d}^{c}{\omega}_{d}-{R}_{d}^{c}{\dot{\omega}}_{d}$$

Substituting ${\dot{\omega}}_{c}$ and ${\dot{\omega}}_{T}$ from Eqs. (1) and (6), also ${\omega}_{c}$ from Eq. (12) into Eq. (14), the relative attitude dynamics is defined as:

15 $${\dot{\omega}}_{rel}={f}_{{\omega}_{rel}}+{I}_{c}^{-1}\left({T}_{c}+{T}_{{d}_{c}}+{T}_{{d}_{TH}}\right)-{I}_{T}^{-1}{R}_{T}^{c}{T}_{{d}_{T}}$$

where ${f}_{{\omega}_{rel}}$ is defined as follows:

16 $${f}_{{\omega}_{rel}}=-{I}_{c}^{-1}\left[\left({\omega}_{rel}+{R}_{T}^{c}{\omega}_{T}\right)\times {I}_{c}\left({\omega}_{rel}+{R}_{T}^{c}{\omega}_{T}\right)+\left({\omega}_{rel}+{R}_{T}^{c}{\omega}_{T}\right)\times {h}_{RW}\right]+\left({\omega}_{r}\times {R}_{T}^{c}{\omega}_{T}\right)+{I}_{T}^{-1}{R}_{T}^{c}\left({\omega}_{T}\times {I}_{T}{\omega}_{T}\right)$$

The relative attitude kinematic is a function of relative angular velocity $\left({\omega}_{rel}\right)$ and relative quaternion parameters $\left({q}_{rel}\right)$ as follows^56^:

18 $${\dot{q}}_{rel}=\frac{1}{2}Q\left({\omega}_{rel}\right){q}_{rel}$$

where $${\overrightarrow{q}}_{rel}={\left[\begin{array}{cc}{q}_{{r}_{v}}^{T}& {q}_{{r}_{4}}\end{array}\right]}^{T} \,\,\epsilon\,\, {\mathfrak{R}}^{4\times 1}$$ represents the relative attitude quaternion. ${q}_{{r}_{4}}$ Indicates the scaler, and ${\overrightarrow{q}}_{{r}_{v}}={\left[{q}_{{r}_{1}}, {q}_{{r}_{2}}, {q}_{{r}_{3}}\right]}^{T} \,\,\epsilon\,\, {\mathfrak{R}}^{3\times 1}$ vector components of the ${\overrightarrow{q}}_{rel}$. $Q\left({\omega}_{rel}\right)$ can be written as Eq. (19).

19 $$Q\left({\omega}_{rel}\right)=\left[\begin{array}{cc}0& -{\omega}_{rel}^{T}\\ {\omega}_{rel}& -S\left({\omega}_{rel}\right)\end{array}\right]$$

The relative attitude quaternion is a function of the chaser and virtual target spacecraft attitude, which satisfy the following equation^77^:

20 $${q}_{rel}={q}_{d}^{-1} \otimes {q}_{c}$$

The symbol $\otimes$ denotes quaternion multiplication. Also, ${q}_{rel}$ satisfies the normalized constraint $\left({q}_{rel}^{T}{q}_{rel}=1\right)$.

The rotation matrix ${R}_{d}^{c}$ can be used to transform an arbitrary vector from the target body coordinate system to the chaser coordinate system, which is expressed in terms of relative quaternion parameters as follows^79^:

21 $${R}_{d}^{c}=\left[\begin{array}{ccc}{q}_{{r}_{1}}^{2}-{q}_{{r}_{2}}^{2}-{q}_{{r}_{3}}^{2}+{q}_{{r}_{4}}^{2}& 2\left({q}_{{r}_{1}}{q}_{{r}_{2}}-{q}_{{r}_{3}}{q}_{{r}_{4}}\right)& 2\left({q}_{{r}_{1}}{q}_{{r}_{3}}+{q}_{{r}_{2}}{q}_{{r}_{4}}\right)\\ 2\left({q}_{{r}_{1}}{q}_{{r}_{2}}+{q}_{{r}_{3}}{q}_{{r}_{4}}\right)& -{q}_{{r}_{1}}^{2}+{q}_{{r}_{2}}^{2}-{q}_{{r}_{3}}^{2}+{q}_{{r}_{4}}^{2}& 2\left({q}_{{r}_{2}}{q}_{{r}_{3}}-{q}_{{r}_{1}}{q}_{{r}_{4}}\right)\\ 2\left({q}_{{r}_{1}}{q}_{{r}_{3}}-{q}_{{r}_{2}}{q}_{{r}_{4}}\right)& 2\left({q}_{{r}_{2}}{q}_{{r}_{3}}+{q}_{{r}_{1}}{q}_{{r}_{4}}\right)& -{q}_{{r}_{1}}^{2}-{q}_{{r}_{2}}^{2}+{q}_{{r}_{3}}^{2}+{q}_{{r}_{4}}^{2}\end{array}\right]$$

In fact, in the case of relative attitude control, it is enough to control the vector component of relative attitude quaternion parameters and relative angular velocity. Thus, only the following equations will be used in the next.

22 $${\left[\begin{array}{c}{\dot{\omega}}_{rel}\\ {\dot{q}}_{{r}_{v}}\end{array}\right]}_{6\times 1}={\left[\begin{array}{c}{f}_{{\omega}_{rel}}\\ \frac{1}{2}\left({q}_{{r}_{4}}{\omega}_{rel}-S\left({\omega}_{rel}\right){q}_{{r}_{v}}\right)\end{array}\right]}_{6\times 1}+{\left[\begin{array}{c}{I}_{c}^{-1}\\ 0\cdot {I}_{3}\end{array}\right]}_{6\times 3}{\left[{T}_{c}+{T}_{{d}_{TH}}+{T}_{{d}_{c}}\right]}_{3\times 1}-{\left[\begin{array}{c}{R}_{T}^{c}{I}_{T}^{-1}\\ 0\cdot {I}_{3}\end{array}\right]}_{6\times 3}{\left[{T}_{{d}_{T}}\right]}_{3\times 1}$$

#### Relative attitude dynamics including uncertainty

Changes in the mass distribution (due to fuel consumption and active mechanism operation) cause uncertainty in the moment of inertia tensor. In addition, unknown precise parameters such as attitude control actuators' misalignment or miscalibration cause uncertainty. So, we can rewrite the more realistic relative attitude dynamics in the form as:

23 $${\dot{\omega}}_{rel}={f}_{{\omega}_{rel}}+{\left(1+{\delta}_{{I}_{c}}\right)}^{-1}{I}_{c}^{-1}\left({\overline{T}}_{c}+{T}_{{d}_{TH}}+{T}_{{d}_{c}}\right)-{\left(1+{\delta}_{{I}_{T}}\right)}^{-1}{I}_{T}^{-1}{R}_{T}^{c}{T}_{{d}_{T}}$$

where ${\delta}_{{I}_{c}}$ and ${\delta}_{{I}_{T}}$ are the uncertainty of the chaser and target spacecraft’s moment of inertia tensor, respectively. ${\overline{T}}_{c}={\left[{\overline{T}}_{{c}_{x}},{\overline{T}}_{{c}_{y}},{\overline{T}}_{{c}_{z}}\right]}^{T}\,\,\epsilon\,\, {R}^{3\times 1}$ is the chaser’s actuator torque including uncertainty (see section "Reaction wheels configuration").

### Reaction wheels modeling

**Figure 6:**
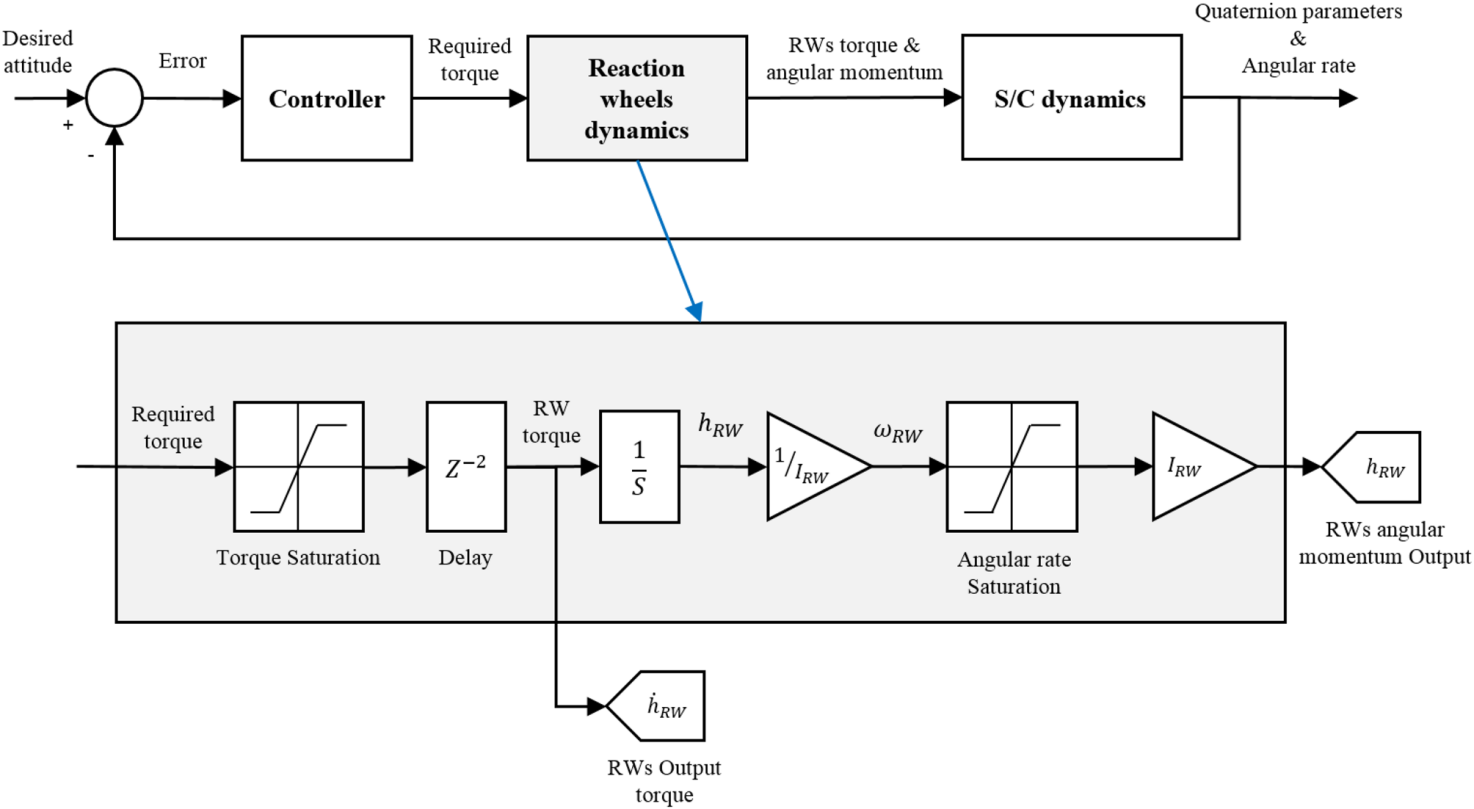
Block diagram of the Reaction wheel.

Reaction wheels are accurate, reliable, and cost-effective actuators that produce smooth and continuous torque. The accuracy of about $0.1^\circ$ and the precision of about $0.001\;^\circ/s$ is achievable with the help of these actuators. The block diagram of the RWs simulation is shown in Fig. 6.

### Rotational disturbances

Gravity-gradient torque, aerodynamic torque, solar radiation torque, and earth magnetic torque are the important external disturbances in LEO.

#### Gravity gradient disturbance torque^77^

Due to the non-homogeneous mass distribution of the satellite, the gravitational force acting over it is not uniform. The gravity gradient torque expressed in the chaser/target spacecraft body coordinate system is given as:

$${T}_{{GG}_{x}}=\frac{3\mu}{2{r}_{0}^{3}}\left({I}_{z}-{I}_{y}\right)\mathrm{sin}\left(2\phi \right){\mathrm{cos}}^{2}\left(\theta \right)$$

$${T}_{{GG}_{y}}=\frac{3\mu}{2{r}_{0}^{3}}\left({I}_{z}-{I}_{x}\right)\mathrm{sin}\left(2\theta \right)\mathrm{cos}\left(\phi \right)$$

24 $${T}_{{GG}_{z}}=\frac{3\mu}{2{r}_{0}^{3}}\left({I}_{x}-{I}_{y}\right)\mathrm{sin}\left(2\theta \right){\mathrm{cos}}^{2}\left(\phi \right)$$

where $\theta$ and $\phi$ are the pitch and roll angles of the chaser/target spacecraft. ${I}_{x}$, ${I}_{y}$ and ${I}_{z}$ are the moment of inertia elements of the chaser/target spacecraft. ${r}_{0}$ denotes the distance between the center of the earth and the chaser/target spacecraft.

#### Atmospheric drag disturbance torque

The aerodynamic torque is produced due to the distance between the chaser/target spacecraft's center of mass and its own aerodynamic center. This disturbance torque is given as:

25 $${T}_{drag}=m{\overrightarrow{a}}_{drag}\left({X}_{CG}-{X}_{ac}\right)$$

where $\left({X}_{CG}-{X}_{ac}\right)$ denotes the offset from the chaser/target spacecraft’s center of gravity (CG) to the aerodynamic center (ac) which is taken to be 0.5 cm for the chaser and 1 cm for the target spacecraft. $m$ represents the mass of the chaser/target spacecraft. ${\overrightarrow{a}}_{drag}$ is the acceleration of drag force that is explained in section "Rigid spacecraft position equation of motion".

#### Solar radiation pressure (SRP) disturbance torque

Solar radiation force is the force produced by the impact of sunlight photons (or the protons, electrons, and ions emitted from the sun) on the surface of the spacecraft. The distance between the center of pressure (CP) and the center of gravity (CG) of the spacecraft is the cause of SRP torque, which is given as^80^:

26 $${T}_{SRP}={F}_{s}\left({x}_{cp}-{x}_{cg}\right)$$

where ${F}_{s}$ is the solar radiation pressure force, and $\left({x}_{cp}-{x}_{cg}\right)$ represents the distance between the center of pressure and the center of gravity of the spacecraft. It is assumed to be $1$ centimeter for the target and $0$ for the chaser spacecraft.

27 $${F}_{s}=\frac{{S}_{ \odot}}{{C}_{l}}{A}_{S}\left(1+q\right)\mathrm{cos}\left({i}_{s}\right)$$

where ${S}_{ \odot}=1367 \frac{w}{{m}^{2}}$ represents the solar constant. ${C}_{l}=300\times {10}^{3}\frac{\mathrm{km}}{\mathrm{s}}$ denotes the speed of light. ${A}_{S}$ Is the absorbing area. ${i}_{s}$ is the incidence angle between the sun vector and the normal vector of the absorbing surface. $0<q<1$ Is a constant which is related to the surface reflectance ($q=1$ for worst-case and $q=0.6$ for typical value).

#### Magnetic disturbance torque

The interaction between the Earth's magnetic field ($B$) and the residual magnetic field of the satellite ($M$) is the main cause of this perturbation. The magnetic disturbance torque expressed in the chaser/target body coordinate system can be estimated as^81^:

28 $${T}_{M}=M\times B$$

where $\overrightarrow{M}={\left[\mathrm{0.25,0.25,0.25}\right]}^{T} {\mathrm{Am}}^{2}$ is assumed as the sum of the individual magnetic moments caused by permanent and induced magnetism and the spacecraft-generated loops. $B$ can be calculated as follows^81^:

29 $$B=\frac{{\mu}_{\otimes}}{{r}_{0}^{3}}{\left(1+3{sin}^{2}\mathsf{\Theta}\right) \,}^\frac{1}{2}$$

where $\mathsf{\Theta}$ is the magnetic latitude measured from the magnetic equator, which can be considered as ${90}^{^\circ}$ in the worst case (at the magnetic pole). ${\mu}_{\otimes}=8.1\times {10}^{15} {\mathrm{Tm}}^{3}$ represents the magnitude of the Earth’s magnetic moment vector along the magnet axial direction^82^. We assumed that the residual magnetic moment only accumulates in the target spacecraft, so the magnetic disturbance torque is only acting on the target spacecraft's motion.

In the end, the total disturbance torque in Eq. (23) can be calculated as follows:

30 $${T}_{{d}_{c}}={T}_{{GG}_{c}}+{T}_{{drag}_{c}} \Rightarrow \Vert {T}_{{d}_{c}}\Vert \le {\gamma}_{1}$$

31 $${T}_{{d}_{T}}={T}_{{GG}_{T}}+{T}_{{drag}_{T}}+{T}_{{SRP}_{T}}+{T}_{{M}_{T}} \Rightarrow \Vert {T}_{{d}_{T}}\Vert \le {\gamma}_{2}$$

### Translational dynamics

#### Rigid spacecraft position equation of motion

We assumed that the target spacecraft is moving in a Keplerian circular orbit, and the chaser spacecraft is able to control its own position. The dynamic modeling of the chaser’s center of mass motion is given by the following equation (which is expressed in ${O}_{{x}_{c}{y}_{c}{z}_{c}}$)^50^:

32 $${m}_{c}{\dot{\mathrm{v}}}_{c}=-{m}_{c}S\left({\omega}_{c}\right){\mathrm{v}}_{c}+{F}_{c}+{F}_{{d}_{c}}$$

where ${m}_{c} \,\,\epsilon\,\, {R}^{1\times 1}$ is the chaser spacecraft mass. ${\overrightarrow{\mathrm{v}}}_{c}={\left[{u}_{\mathrm{c}}, {v}_{\mathrm{c}}, {w}_{\mathrm{c}}\right]}^{T}\,\,\epsilon\,\, {R}^{3\times 1}$ denotes the velocity vector. ${\overrightarrow{F}}_{c}={\left[{F}_{{c}_{x}}, {F}_{{c}_{y}}, {F}_{{c}_{z}}\right]}^{T}\,\,\epsilon\,\, {R}^{3\times 1}$ and ${\overrightarrow{F}}_{{d}_{c}}={\left[{F}_{{{d}_{c}}_{x}}, {F}_{{{d}_{c}}_{y}}, {F}_{{{d}_{c}}_{z}}\right]}^{T}\,\,\epsilon\,\, {R}^{3\times 1}$ are the control and disturbance forces acting on chaser spacecraft, respectively.

Equation 33 presents the chaser’s translational motion kinematics (expressed in the ${O}_{{x}_{c}{y}_{c}{z}_{c}}$)^50^:

33 $${\dot{r}}_{c}={\mathrm{v}}_{c}-S\left({\omega}_{c}\right){r}_{c}$$

where ${\overrightarrow{r}}_{c}={\left[{r}_{{c}_{x}}, {r}_{{c}_{y}}, {r}_{{c}_{z}}\right]}^{T}\,\,\epsilon\,\, {R}^{3\times 1}$ is the position vector of the chaser spacecraft in its own coordinate system.

In a similar way, the dynamics and kinematics equation of target spacecraft motion can be described in ${O}_{{x}_{T}{y}_{T}{z}_{T}}$ as follows^50^:

34 $${m}_{T}{\dot{\mathrm{v}}}_{T}=-{m}_{T}S\left({\omega}_{T}\right){\mathrm{v}}_{T}+{F}_{{d}_{T}}$$

Again, ${m}_{T} \,\,\epsilon\,\, {R}^{1\times 1}$ is the target spacecraft mass, ${\overrightarrow{\mathrm{v}}}_{T}={\left[{u}_{\mathrm{T}}, {v}_{\mathrm{T}}, {\mathrm{w}}_{\mathrm{T}}\right]}^{T}\,\,\epsilon\,\, {R}^{3\times 1}$ is the target velocity vector in the body frame and ${\overrightarrow{F}}_{{d}_{T}}={\left[{F}_{{{d}_{T}}_{x}}, {F}_{{{d}_{T}}_{y}}, {F}_{{{d}_{T}}_{z}}\right]}^{T}\,\,\epsilon\,\, {R}^{3\times 1}$ is the external disturbance force acting on the target spacecraft^50^.

35 $${\dot{r}}_{T}={\mathrm{v}}_{T}-S\left({\omega}_{T}\right){r}_{T}$$

where ${\overrightarrow{r}}_{T}=\left[{r}_{{T}_{x}}, {r}_{{T}_{y}}, {r}_{{T}_{z}}\right]\,\,\epsilon\,\, {R}^{3\times 1}$ denotes the position vector of the target spacecraft.

#### Relative position dynamics

In relative position dynamics, both spacecraft are treated as point masses. The equations will be expressed in the chaser spacecraft’s body coordinate system Due to relative navigation considerations.

The relative position and translational velocity are given as:

36 $${r}_{rel}={r}_{c}-{R}_{T}^{c}{r}_{T}$$

37 $${\mathrm{v}}_{rel}={\mathrm{v}}_{c}-{R}_{T}^{c}{\mathrm{v}}_{T}$$

where ${{\overrightarrow{r}}_{rel}=\left[{r}_{{rel}_{x}},{r}_{{rel}_{y}},{r}_{{rel}_{z}}\right]}^{T} \,\,\epsilon\,\, {{\varvec{R}}}^{3\times 1}$ and ${{\overrightarrow{\mathrm{v}}}_{rel}=\left[{u}_{rel},{v}_{rel},{w}_{rel}\right]}^{T} \,\,\epsilon\,\, {{\varvec{R}}}^{3\times 1}$ are the relative velocity and position vectors of the chaser and the target spacecraft (expressed in the target body frame).

The time derivative of the relative velocity is given as:

38 $${\dot{\mathrm{v}}}_{rel}={\dot{\mathrm{v}}}_{c}+S\left({\omega}_{rel}\right){R}_{T}^{c}{\mathrm{v}}_{T}-{R}_{T}^{c}{\dot{\mathrm{v}}}_{T}$$

Substituting Eqs. (32) and (34) into (38), yields:

39 $${\dot{\mathrm{v}}}_{rel}=\frac{{F}_{c}}{{m}_{c}}+\frac{{F}_{{d}_{c}}}{{m}_{c}}-S\left({\omega}_{c}\right){\mathrm{v}}_{c}+\left(S\left({\omega}_{rel}\right)+S\left({\omega}_{T}\right)\right){R}_{T}^{c}{\mathrm{v}}_{T}-{R}_{T}^{c}\frac{{F}_{{d}_{T}}}{{m}_{T}}$$

Similarly, the relative kinematics can be driven as follows:

40 $${\dot{r}}_{rel}={\mathrm{v}}_{c}-S\left({\omega}_{c}\right){r}_{c}+S\left({\omega}_{rel}\right){R}_{T}^{c}{r}_{T}-{R}_{T}^{c}\left({\mathrm{v}}_{T}-S\left({\omega}_{T}\right){r}_{T}\right)$$

$${\dot{r}}_{rel}={\mathrm{v}}_{rel}-S\left({\omega}_{rel}\right){r}_{rel}+S\left({\omega}_{rel}\right){R}_{T}^{c}{r}_{T}$$

#### Relative position dynamics including uncertainty

Equation (39) is valid only under the ideal condition that there are no disturbances and uncertainties. However, from a practical point of view, the space environment is not free of disturbance forces. Also, spacecraft control is always affected by different uncertainties (such as mass and thrusters force), and relative navigation is also not free of error. Thus, Eq. (41) presents a more realistic equation of relative motion.

41 $${\dot{\mathrm{v}}}_{rel}={\left(1+{\delta}_{{m}_{c}}\right)}^{-1}\left(\frac{{F}_{c}+{F}_{{d}_{c}}}{{m}_{c}}\right)-{\left(1+{\delta}_{{m}_{T}}\right)}^{-1}{R}_{T}^{c}\frac{{F}_{{d}_{T}}}{{m}_{T}}-S\left({\omega}_{c}\right){\mathrm{v}}_{c}+\left(\left(1+{\delta}_{{\omega}_{rel}}\right)S\left({\omega}_{rel}\right)+S\left({\omega}_{T}\right)\right){R}_{T}^{c}{\mathrm{v}}_{T}$$

where, ${\delta}_{{m}_{c}}$ and ${\delta}_{{m}_{T}}$ are the uncertainty of the chaser and target spacecraft mass. ${\delta}_{{\mathrm{v}}_{rel}}$ is the uncertainty and error of the relative navigation measurement sensors.

### Thruster modeling

**Figure 7:**
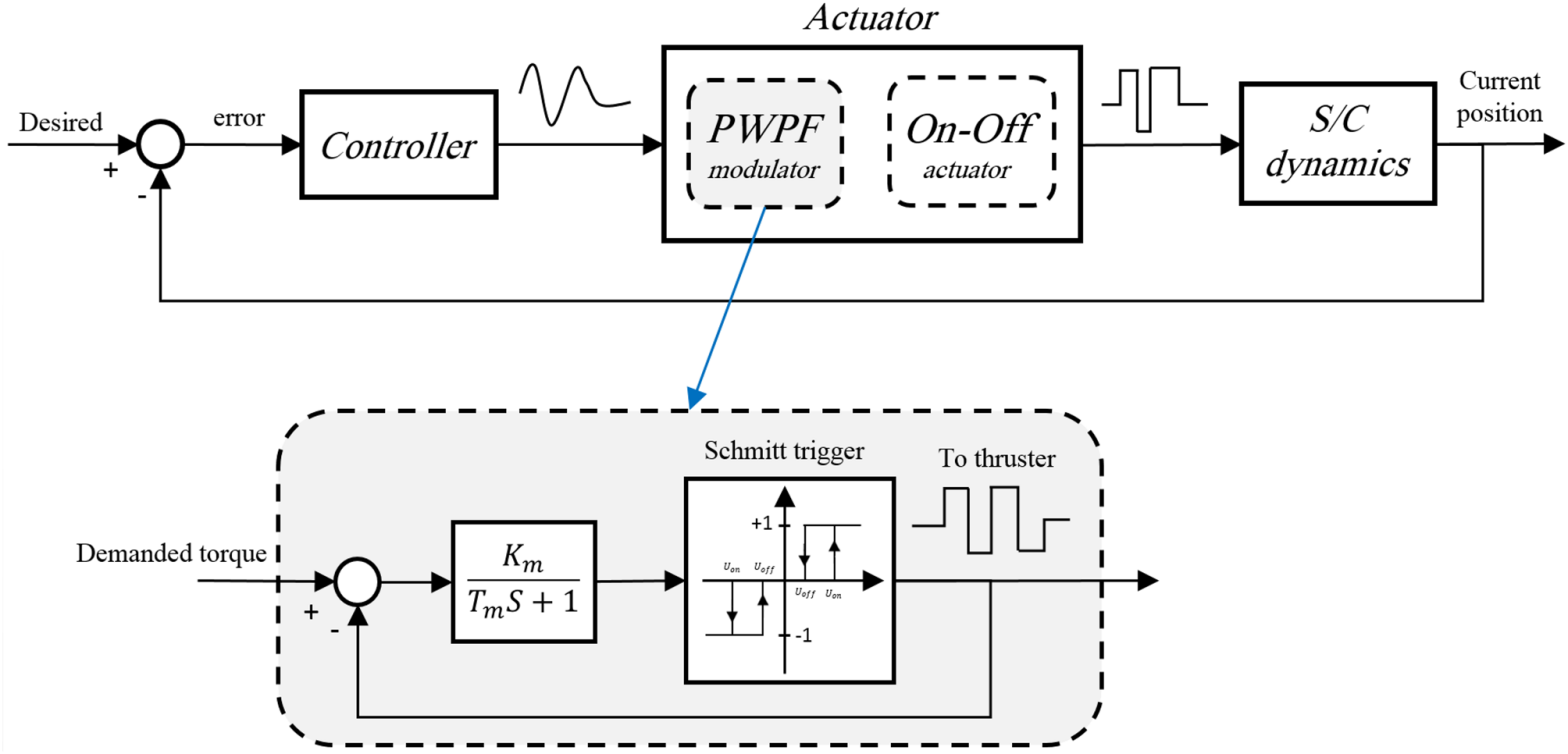
Block diagram of the PWPF modulator.

On–off thrusters (also called reaction thrusters) are widely used in spacecraft position/attitude controllers, however, it can be fairly challenging to generate accurate on–off commands from calculated continuous control signals. The most well-known and suitable technique for achieving this goal is the pulse-width pulse frequency (PWPF) modulator. Low susceptibility to noise and perturbations, high flexibility in adjusting, and reduced fuel consumption are the main advantages of this modulator^83^. As shown in Fig. 7, the PWPF modulator is generally formed by a Schmitt trigger along with a low-pass filter in a loop where ${K}_{m}$ and ${T}_{m}$ in Fig. 7 are the pre-filter gain and time constant, respectively. ${U}_{on}$ and ${U}_{off}$ are the activation and deactivation of the Schmitt trigger (see^84^ for more details about the PWPF modulator and its parameter setting).

### Translational disturbances

Orbital disturbances, including earth oblateness (${J}_{2}$ effect), atmospheric drag, solar radiation pressure, and third body (especially moon and Jupiter) gravity, affect both spacecraft’s translational motion. The impact of each of these disturbances strongly depends on the spacecraft’s shape and orbit height. For example, atmospheric drag and the J2 effect are the most effective disturbances in LEO. Solar radiation pressure and third-body gravity are the most effective disturbances, in GEO. The shape and size of the spacecraft play an important role in the magnitude of the solar radiation pressure and atmospheric drag disturbance.

In this paper, we only considered the atmospheric drag and ${J}_{2}$ disturbances due to the orbit height of the target spacecraft.

#### Atmospheric drag

The atmospheric drag acceleration can be modeled as follows^81^:

42 $${\overrightarrow{a}}_{drag}=\frac{1}{2}\frac{\rho {\left|{\overrightarrow{\mathrm{v}}}_{d}\right|}^{2}S{C}_{D}}{m}\frac{{\overrightarrow{\mathrm{v}}}_{d}}{\left|{\overrightarrow{\mathrm{v}}}_{d}\right|}$$

where $\rho$ is the atmospheric density in the target’s orbit height. ${C}_{D}$ indicates the drag coefficient, $S$ represents the area that is normal to the air flow, $m$ is considered as the spacecraft mass, and ${\overrightarrow{\mathrm{v}}}_{d}$ is the spacecraft velocity vector with respect to the rotating atmosphere. It’s clear that ${\overrightarrow{a}}_{drag}$ can be calculated for both chasers and target spacecraft.

In low altitudes (from 0 to 1000 km), the atmosphere density $\left(\rho \right)$ can be calculated as Eq. (43):

43 $$\rho ={\rho}_{0}EXP\left(-\frac{{h}_{ellp}-{h}_{0}}{H}\right)$$

where ${\rho}_{0}$ and ${h}_{0}$ are reference density and reference altitude, respectively. ${h}_{ellp}$ denotes the actual altitude of the orbit and $H$ is the scale height^81^.

The relative velocity vector can be calculated as follows:

44 $${\overrightarrow{\mathrm{v}}}_{{d}_{T/c}}={\overrightarrow{\mathrm{v}}}_{T/c}-{\overrightarrow{\mathrm{v}}}_{atm}$$

where ${\overrightarrow{\mathrm{v}}}_{T/c}$ can be the velocity vector of the chaser or target spacecraft in their own body frame. ${\overrightarrow{\mathrm{v}}}_{atm}$ is the velocity vector of the atmosphere.

The orbital velocity of the target spacecraft is expressed in the target reference coordinate system $({\left.{\overrightarrow{\mathrm{v}}}_{T}\right|}^{TR})$ can be calculated as follows^81^:

45 $${\left.{\overrightarrow{\mathrm{v}}}_{T}\right|}^{TR}={\left[{v}_{{T}_{x}}, 0, 0\right]}^{T}$$

where ${v}_{{T}_{x}}=\sqrt{\mu/\left|{\overrightarrow{r}}_{T}\right|}$ denotes the target spacecraft’s velocity(mean motion), which is moving in a Keplerian circular orbit. Thus, ${\overrightarrow{\mathrm{v}}}_{{d}_{T}}$ for the target spacecraft (expressed in ${O}_{{x}_{t}{y}_{t}{z}_{t}}$) is:

46 $${\overrightarrow{\mathrm{v}}}_{{d}_{T}}={{R}_{TR}^{T}\left[{v}_{{T}_{y}}-\left|{\overrightarrow{\mathrm{v}}}_{atm}\right|, 0, 0\right]}^{T}$$

where ${R}_{TR}^{T}$ is the rotation matrix between the target reference and target body coordinate system the relative velocity of the chaser spacecraft with respect to the atmosphere, expressed in the ${O}_{{x}_{c}{y}_{c}{z}_{c}}$ can be calculated as follows^81^:

47 $${\overrightarrow{\mathrm{v}}}_{{d}_{c}}={\dot{\overrightarrow{r}}}_{c}-{R}_{ECI}^{C}{\overrightarrow{\mathrm{v}}}_{atm}$$

${R}_{ECI}^{C}$ denotes the transformation matrix from ECI to the chaser spacecraft body coordinate system.

The atmosphere is assumed to rotate with the earth’s angular velocity. Thus, the velocity vector of the atmosphere at each altitude can be calculated as:

48 $${\overrightarrow{\mathrm{v}}}_{atm}={\overrightarrow{\omega}}_{\oplus}\times {\overrightarrow{r}}_{T/c}$$

${\overrightarrow{r}}_{T/c}$ denotes the radial vector of the chaser or target spacecraft expressed in the ECI coordinate system. and ${\overrightarrow{\omega}}_{\oplus}={\left[\mathrm{0,0},7.26\times {10}^{-5}\right]}^{T} \,\,\epsilon\,\, {R}^{3\times 1}$ is the earth’s angular velocity.

**Figure 8:**
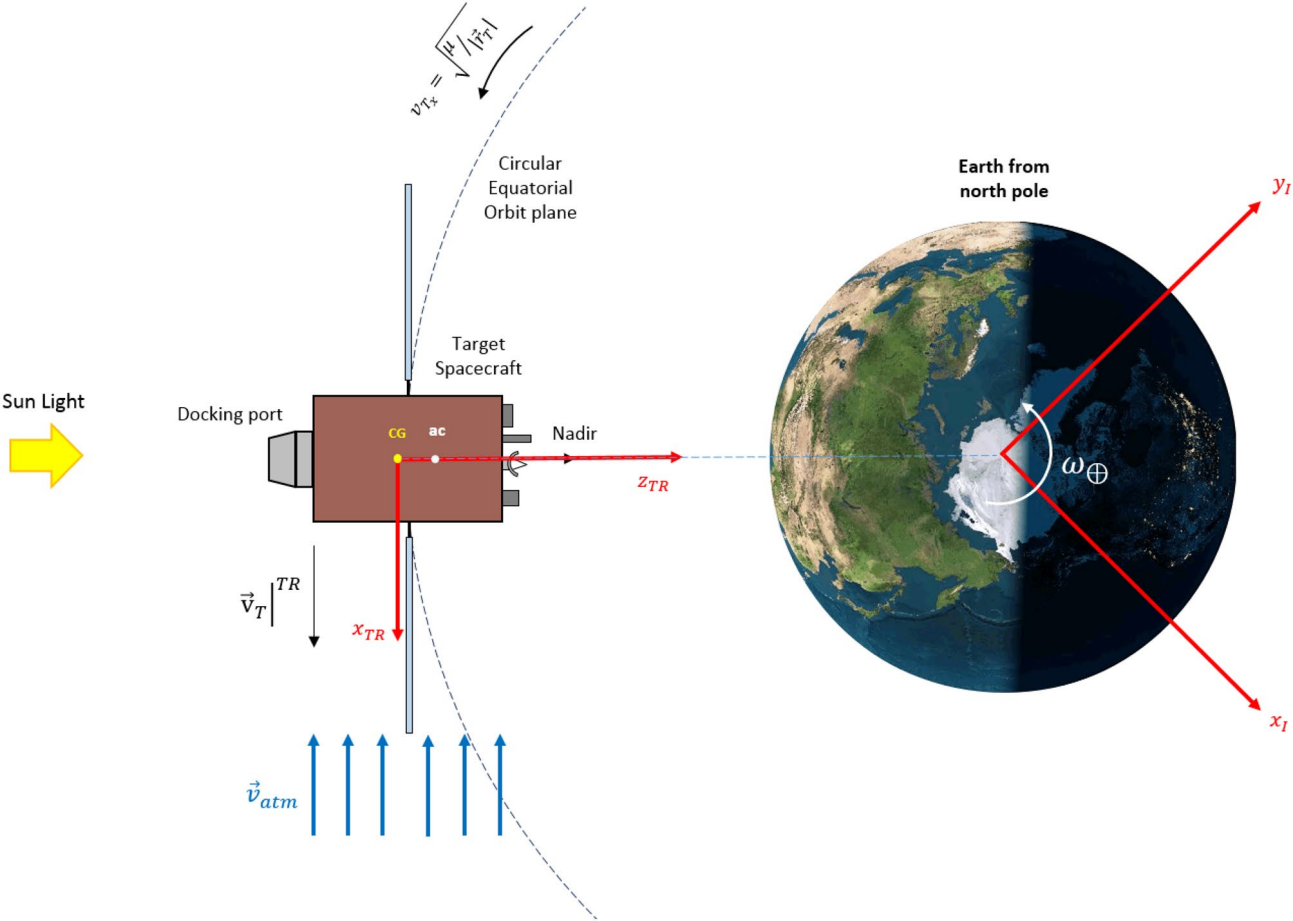
Schematic of target spacecraft motion in the presence of atmosphere flow.

Figure 8 shows the relative velocity of the target spacecraft and atmosphere.

#### Gravitational perturbation

In fact, the earth is an oblate spheroid with a nonhomogeneous mass distribution. Thus, spacecraft orbiting in an Earth-Referenced orbit, sense varying gravitational acceleration in different positions. The perturbing gravitational acceleration vector $\left({\overrightarrow{a}}_{gravity}\right)$ acting on chaser spacecraft (expressed in the chaser coordinate system) can be calculated as follows^81^:

49 $${\overrightarrow{a}}_{gravity}=\left(\frac{3}{2}\frac{\mu {J}_{2}{R}_{E}^{2}}{{\left|{\overrightarrow{r}}_{c}\right|}^{5}}{\left[{r}_{{c}_{x}}\left(5\frac{{r}_{{c}_{x}}^{2}}{{\left|{\overrightarrow{r}}_{c}\right|}^{2}}-1\right),{r}_{{c}_{y}}\left(5\frac{{r}_{{c}_{y}}^{2}}{{\left|{\overrightarrow{r}}_{c}\right|}^{2}}-1\right),{r}_{{c}_{z}}\left(5\frac{{r}_{{c}_{z}}^{2}}{{\left|{\overrightarrow{r}}_{c}\right|}^{2}}-3\right)\right]}^{T}\right)$$

where ${J}_{2}=0.00108263$ is the second zonal harmonic of the earth, and ${R}_{E}=6378 \left[km\right]$ is considered as the radius of the earth.

Note: The target spacecraft is moving in an equatorial orbit ($i=0$), and consequently it doesn’t sense the gravitational perturbation acceleration.

It should be noted that translational disturbances are bounded ($\Vert {F}_{{d}_{c}}\Vert \le {\gamma}_{3}\,\, and \,\,\Rightarrow \Vert {F}_{{d}_{T}}\Vert \le {\gamma}_{4}$).

## Controller design

The sliding mode control (SMC) strategy is known as an efficient tool for achieving a robust controller. Indeed, the SMC method is found as a trade-off between accuracy, robustness, and simplicity in designing a controller for nonlinear dynamic systems under disturbance and uncertain conditions.

### Relative attitude motion control

In relative attitude motion, not only the control input uncertainty but also the misalignment of the actuators is taken into account.

#### Reaction wheels configuration

As mentioned before, we assumed that the chaser spacecraft is equipped with three reaction wheels. In an ideal condition, the wheels are aligned with the body coordinate axes. However, in real-world conditions, the alignment of the actuators will never be perfect (due to manufacturing tolerances, human error in the testing and assembling phase, or warping of the spacecraft structure during the launch phase). Consequentially, the actuators’ misalignment is known as an important source of control error. In this section, the reaction wheels’ misalignment is taken into consideration.

As shown in Fig. 7, the reaction wheel mounted on the $X$ axis $\left({RW}_{x}\right)$ is deviated from the nominal direction by constant angles ${\alpha}_{1}$ and ${\beta}_{1}$. The reaction wheel mounted on the $Y$ axis $\left({RW}_{y}\right)$ is deviated from the nominal direction by constant angles ${\alpha}_{2}$ and ${\beta}_{2}$. The reaction wheel mounted on the $Z$ axis $\left({RW}_{z}\right)$ is deviated from the nominal direction by constant angles ${\alpha}_{3}$ and ${\beta}_{3}$. Considering the alignment errors, the real control torque can be expressed as follows:

50 $${T}_{c}={T}_{{RW}_{x}}\left[\begin{array}{c}\mathrm{cos}{{\alpha}_{RW}}_{1}\\ \mathrm{sin}{{\alpha}_{RW}}_{1}\mathrm{cos}{\beta}_{{RW}_{1}}\\ \mathrm{sin}{{\alpha}_{RW}}_{1}\mathrm{sin}{\beta}_{{RW}_{1}}\end{array}\right]+{T}_{{RW}_{y}}\left[\begin{array}{c}\mathrm{sin}{{\alpha}_{RW}}_{2}\mathrm{cos}{\beta}_{{RW}_{2}}\\ \mathrm{cos}{{\alpha}_{RW}}_{2}\\ \mathrm{sin}{{\alpha}_{RW}}_{2}\mathrm{sin}{\beta}_{{RW}_{2}}\end{array}\right]+{T}_{{RW}_{z}}\left[\begin{array}{c}\mathrm{sin}{{\alpha}_{RW}}_{3}\mathrm{cos}{\beta}_{{RW}_{3}}\\ \mathrm{sin}{{\alpha}_{RW}}_{3}\mathrm{sin}{\beta}_{{RW}_{3}}\\ \mathrm{cos}{{\alpha}_{RW}}_{3}\end{array}\right]$$

where ${T}_{{RW}_{x}}, {T}_{{RW}_{y}},\,\, and\,\, {T}_{{RW}_{z}}$ are the torque generated by reaction wheels.

As mentioned in^85^ the reaction wheels are prone to some faults and uncertainties. Taking actuators' uncertainty and misalignments, Eq. (50) can be rewritten as Eq. (51).

**Figure 9:**
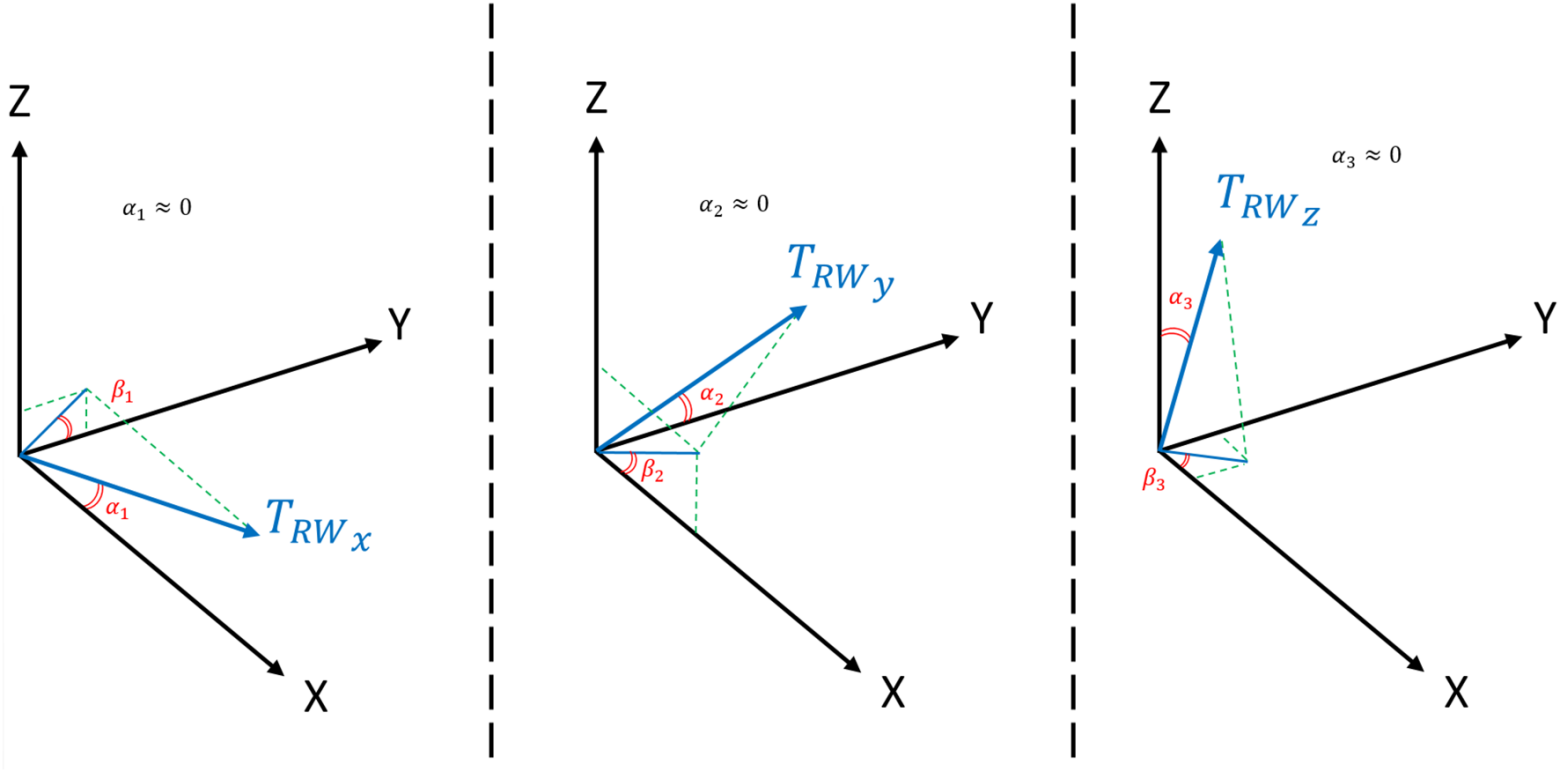
Reaction wheels’ misalignment.

Figure 9 shows the reaction wheels’ tilt angle.

51 $${T}_{c}={D}_{a}\left({T}_{RW}+{\widetilde{T}}_{RW}\right)$$

where ${\widetilde{T}}_{RW}={\left[{\widetilde{T}}_{{RW}_{x}}, {\widetilde{T}}_{{RW}_{y}}, {\widetilde{T}}_{{RW}_{z}}\right]}^{T} \,\,\epsilon\,\, {\mathfrak{R}}^{3\times 1}$ is the reaction wheels torque uncertainty. ${D}_{a}\,\,\epsilon\,\, {\mathfrak{R}}^{3\times 3}$ denotes the allocation matrix which is defined as follows:

52 $${D}_{a}={{D}_{a}}_{0}+{{D}_{a}}_{1}$$

52a $${{D}_{a}}_{0}=\left[\begin{array}{ccc}1& 0& 0\\ 0& 1& 0\\ 0& 0& 1\end{array}\right]$$

52b $${{D}_{a}}_{1}=\left[\begin{array}{ccc}\mathrm{cos}{\alpha}_{1}-1& \mathrm{sin}{\alpha}_{2}\mathrm{cos}{\beta}_{2}& \mathrm{sin}{\alpha}_{3}\mathrm{cos}{\beta}_{3}\\ \mathrm{sin}{\alpha}_{1}\mathrm{cos}{\beta}_{1}& \mathrm{cos}{\alpha}_{2}-1& \mathrm{sin}{\alpha}_{3}\mathrm{sin}{\beta}_{3}\\ \mathrm{sin}{\alpha}_{1}\mathrm{sin}{\beta}_{1}& \mathrm{sin}{\alpha}_{2}\mathrm{sin}{\beta}_{2}& \mathrm{cos}{\alpha}_{3}-1\end{array}\right]$$

The point is both ${\widetilde{T}}_{RW}$ and ${D}_{a}$ are bounded. Indeed, positive constants ${\gamma}_{5}$ and ${\gamma}_{6}$ can be found such that $\Vert {\widetilde{T}}_{RW}\Vert \le {\gamma}_{5}$ and $\Vert {\mathrm{D}}_{a}\Vert \le {\gamma}_{6}$.

#### Attitude motion controller

The controller format of the relative attitude motion is given as follows (actually, ${u}_{A}$ is equal to the ${T}_{c}$):

53 $${u}_{A}={u}_{{0}_{a}}+{u}_{{s}_{a}}$$

${u}_{{0}_{a}}={\left[{u}_{{0}_{1}}, {u}_{{0}_{2}}, {u}_{{0}_{3}}\right]}^{T}\,\,\epsilon\,\, {\mathfrak{R}}^{3\times 1}$ is a nonlinear feedback control input that is effective for the ideal system (in the absence of uncertainty and disturbances) that keeps the states on the sliding surface. ${u}_{{s}_{a}}={\left[{u}_{{s}_{1}}, {u}_{{s}_{2}}, {u}_{{s}_{3}}\right]}^{T}\,\,\epsilon\,\, {\mathfrak{R}}^{3\times 1}$ is the control input to deal with external disturbances and uncertainties.

Note: Traditional sliding mode controllers employ linear sliding surfaces, which may not be compatible with nonlinear plants' overall dynamic properties. When the error is large, the sliding mechanism might not occur as a result of actuator saturation, or instability might even be produced. Additionally, a linear sliding surface could result in significant chattering in digital implementation. This issue can be substantially resolved by employing a non-linear sliding surface. Nonlinear sliding surfaces could be used in place of linear sliding surfaces to address these issues^87^. Moreover, utilizing a linear sliding surface can result in instability when increasing the gain from a narrow bound, whereas non-linear sliding surfaces provide more flexibility (wider range) in selecting the gain. This issue is especially effective in increasing the convergence rate^88^.

The nonlinear sliding surface selected for attitude motion control is defined as follows:

54 $${S}_{A}={\dot{e}}_{A}+{K}_{A}{e}_{A}erf\left({K}_{B}{q}_{{r}_{4}}\right)$$

where ${S}_{A}={\left[{s}_{{a}_{x}},{s}_{{a}_{y}},{s}_{{a}_{z}}\right]}^{T} \,\,\epsilon\,\, {\mathfrak{R}}^{3\times 1}$ is considered as the sliding surface. Also, ${e}_{A}={q}_{{r}_{v}}={\left[{q}_{{r}_{1}},{q}_{{r}_{2}},{q}_{{r}_{3}}\right]}^{T} \,\,\epsilon\,\, {\mathfrak{R}}^{3\times 1}$ and ${\dot{e}}_{A}={\omega}_{rel}={\left[{\omega}_{{r}_{x}},{\omega}_{{r}_{y}},{\omega}_{{r}_{z}}\right]}^{T} \,\,\epsilon\,\, {\mathfrak{R}}^{3\times 1}$ are the attitude error and angular velocity error, respectively.

${K}_{A}={\left[{k}_{{a}_{1}},{k}_{{a}_{2}},{k}_{{a}_{3}}\right]}^{T} \,\,\epsilon\,\, {\mathfrak{R}}^{3\times 1}$ and ${K}_{B}={\left[{k}_{{b}_{1}},{k}_{{b}_{2}},{k}_{{b}_{3}}\right]}^{T} \,\,\epsilon\,\, {\mathfrak{R}}^{3\times 1}$ are constant coefficients.$erf\left(Z\right)$ also called “error function” or “Gauss error function”, is an odd and bounded function that defines as follows:

55 $$erf\left(Z\right)=\frac{2}{\sqrt{\pi}}{\int}_{0}^{z}{e}^{{-t}^{2}}dt$$

where $t$ is the time.

**Figure 10:**
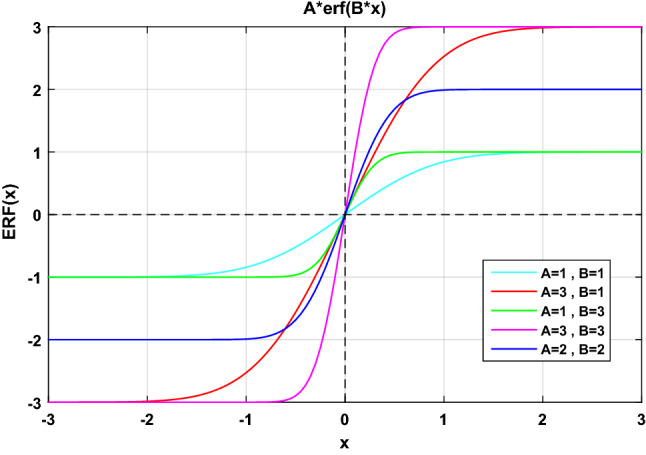
Plot of the error function.

Figure 10 shows the error (erf) function plot.

The time derivative of the sliding surface is:

56 $${\dot{S}}_{A}={\dot{\omega}}_{rel}+{K}_{A}{q}_{{r}_{v}}\frac{d}{dt}\left(erf\left({K}_{B}{q}_{{r}_{4}}\right)\right)$$

Thus, by substituting ${\dot{\omega}}_{rel}$ from Eq. (15) (in the absence of the uncertainties and disturbances) in Eq. (56) the ${u}_{{0}_{a}}$ calculates as Eq. (58).

57 $${\dot{S}}_{A}={f}_{{\omega}_{rel}}+{I}_{c}^{-1}{{D}_{a}}_{0}{u}_{{0}_{a}}+{K}_{A}{q}_{{r}_{v}}\frac{d}{dt}\left(erf\left({K}_{B}{q}_{{r}_{4}}\right)\right)$$

58 $${u}_{{0}_{a}}=-{I}_{c}{D}_{{a}_{0}}^{-1}\left[{f}_{{\omega}_{rel}}+{K}_{A}{q}_{{r}_{v}}\frac{d}{dt}\left(erf\left({K}_{B}{q}_{{r}_{4}}\right)\right)\right]$$

To calculate the term ${u}_{{s}_{a}}=-{d}_{A}sign({S}_{A})$, all uncertainties and disturbances are considered. ${d}_{A}={\left[{d}_{{a}_{1}}, {d}_{{a}_{2}}, {d}_{{a}_{3}}\right]}^{T} \,\,\epsilon\,\, {\mathfrak{R}}^{3\times 1}$ will be calculated in such a way as to ensure the stability of the controller against bounded uncertainties (both parametric and actuator uncertainties) and external disturbances.

We defined the candidate Lyapunov function for relative attitude motion as:

59 $${V}_{A}=\frac{1}{2}{s}_{A}^{2}$$

As is clear, the ${V}_{A}$ is a positive definite. Based on the Lyapunov stability theory we should choose the ${d}_{A}$ in such a way that ${\dot{V}}_{A}$ turns to a negative definite function.

60 $${\dot{V}}_{A}={s}_{A}{\dot{s}}_{A}\le -{\lambda}_{A}\left|{s}_{A}\right|$$

61 $${s}_{A}\left({\dot{\omega}}_{rel}+{K}_{A}{q}_{{r}_{v}}\frac{d}{dt}\left(erf\left({K}_{B}{q}_{{r}_{4}}\right)\right)\right)\le -{\lambda}_{A}\left|{s}_{A}\right|$$

62 $${s}_{A}\left({f}_{{\omega}_{rel}}+{\left(1+{\delta}_{{I}_{c}}\right)}^{-1}{I}_{c}^{-1}{u}_{A}+{\Delta}_{A}+{K}_{A}{q}_{{r}_{v}}\frac{d}{dt}\left(erf\left({K}_{B}{q}_{{r}_{4}}\right)\right)\right)\le -{\lambda}_{A}\left|{s}_{A}\right|$$

where ${\overrightarrow{\lambda}}_{A}={\left[{\lambda}_{{A}_{x}}, {\lambda}_{{A}_{y}}, {\lambda}_{{A}_{z}}\right]}^{T}\,\,\epsilon\,\, {\mathfrak{R}}^{3\times 1}$ is a positive constant. ${\Delta}_{A}$ is defined as:

63 $${\Delta}_{A}={\left(1+{\delta}_{{I}_{c}}\right)}^{-1}{I}_{c}^{-1}\left({T}_{{d}_{c}}+{T}_{{d}_{TH}}\right)-{R}_{T}^{c}{\left(1+{\delta}_{{I}_{T}}\right)}^{-1}{I}_{T}^{-1}{T}_{{d}_{T}}$$

Substituting Eq. (53) in (62) gives:

64 $${s}_{A}\left({f}_{{\omega}_{rel}}+{\left(1+{\delta}_{{I}_{c}}\right)}^{-1}{I}_{c}^{-1}\left[{{D}_{a}}_{0}{u}_{{0}_{a}}+{D}_{a}{\widetilde{T}}_{RW}+{{D}_{a}}_{1}{T}_{RW}+{u}_{{s}_{a}}\right]+{\Delta}_{A}+{K}_{A}{q}_{{r}_{v}}\frac{d}{dt}\left(erf\left({K}_{B}{q}_{{r}_{4}}\right)\right)\right)\le -{\lambda}_{A}\left|{s}_{A}\right|$$

The above equation is arranged as follows:

65 $${s}_{A}\left(\left(1-\frac{1}{{{D}_{a}}_{0}\left(1+{\delta}_{{I}_{c}}\right)}\right)\left({f}_{{\omega}_{rel}}+{K}_{A}{q}_{{r}_{v}}\frac{d}{dt}\left(erf\left({K}_{B}{q}_{{r}_{4}}\right)\right)\right)+{\Delta}_{B}+{\Delta}_{A}+{\left(1+{\delta}_{{I}_{c}}\right)}^{-1}{I}_{c}^{-1}{u}_{{s}_{a}}\right)\le -{\lambda}_{A}\left|{s}_{A}\right|$$

where ${\Delta}_{B}$ is given as follows:

66 $${\Delta}_{B}={\left(1+{\delta}_{{I}_{c}}\right)}^{-1}{I}_{c}^{-1}\left({D}_{a}{\widetilde{T}}_{RW}+{{D}_{a}}_{1}{T}_{RW}\right)$$

Finally, the ${d}_{A}$ which grantee the stability of the controller against disturbance and uncertainties is given as:

67 $${d}_{A}\ge \left(1+{\delta}_{{I}_{c}}\right){I}_{c}\left({\lambda}_{A}+\left|\left(\frac{{\delta}_{{I}_{c}}}{1+{\delta}_{{I}_{c}}}\right)\left({f}_{{\omega}_{rel}}+{K}_{A}{q}_{{r}_{v}}\frac{d}{dt}\left(erf\left({K}_{B}{q}_{{r}_{4}}\right)\right)\right)\right|+\left|{\Delta}_{A}\right|\right)+\left|\left({D}_{a}{\widetilde{T}}_{RW}+{{D}_{a}}_{1}{T}_{RW}\right)\right|$$

Indeed, ${d}_{A}$ guarantees the controller stability against actuator misalignment, control input, and parametric uncertainties.

### Relative translational motion control

**Figure 11:**
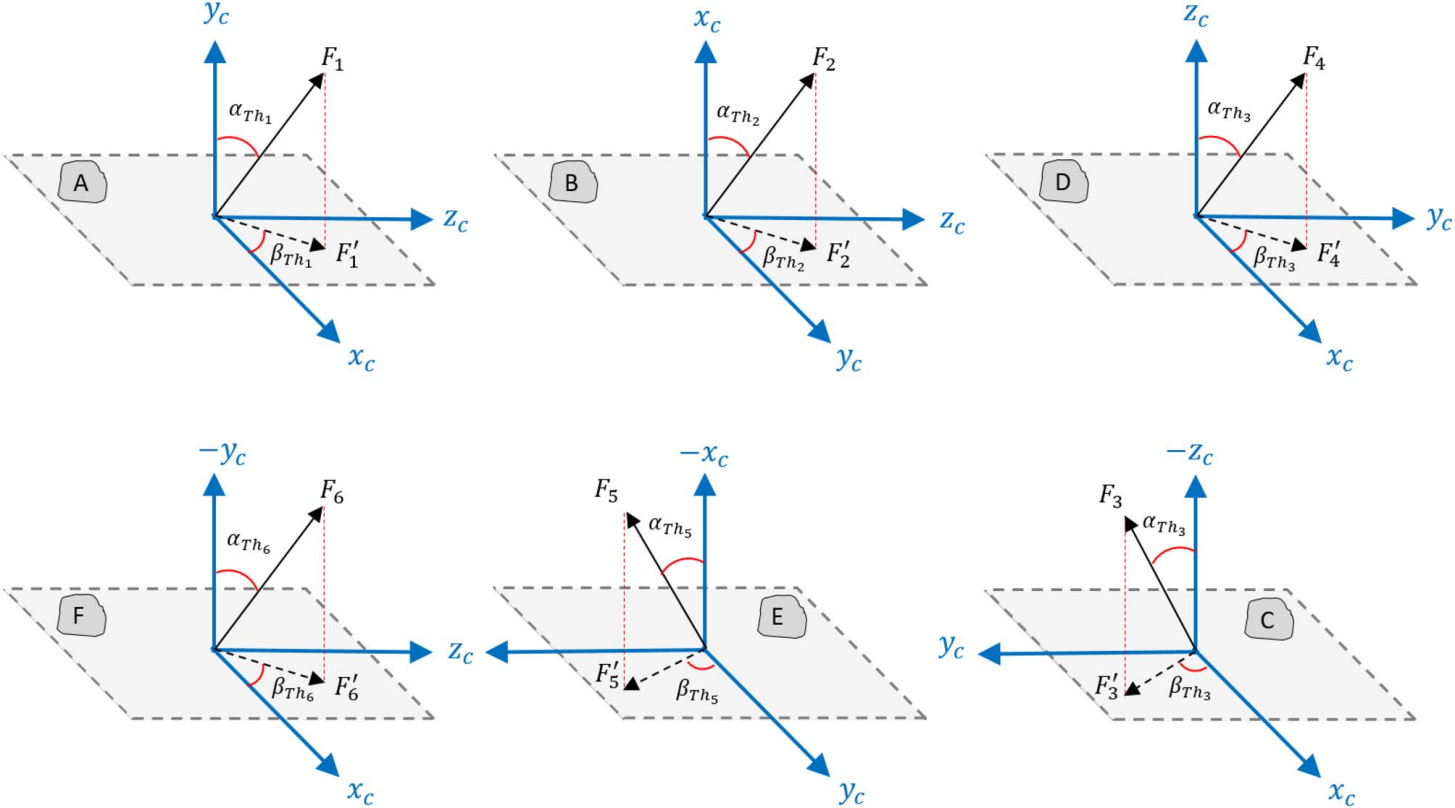
Thrust vector misalignment.

**Figure 12:**
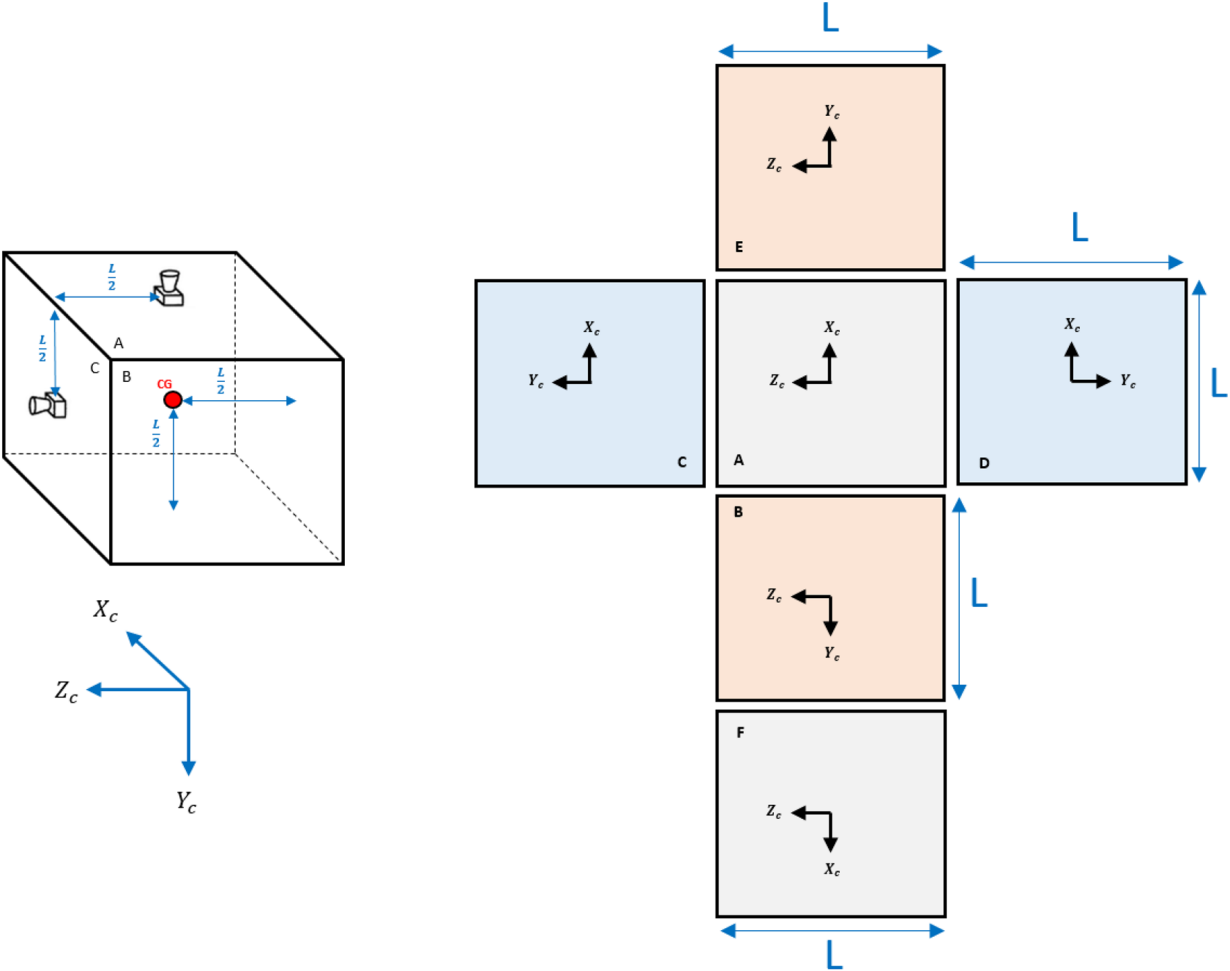
Thrusters arm from CG.

Considering the deviation of the thrusters (caused by installation error) the thrust vector expressed in the chaser body coordinate system can be written as:

68 $${F}_{c}={F}_{1}\left[\begin{array}{c}\mathrm{sin}{\alpha}_{{Th}_{1}}\mathrm{cos}{\beta}_{{Th}_{1}}\\ \mathrm{cos}{\alpha}_{{Th}_{1}}\\ \mathrm{sin}{\alpha}_{{Th}_{1}}\mathrm{sin}{\beta}_{{Th}_{1}}\end{array}\right]+{F}_{2}\left[\begin{array}{c}\mathrm{cos}{\alpha}_{{Th}_{2}}\\ \mathrm{sin}{\alpha}_{{Th}_{2}}\mathrm{cos}{\beta}_{{Th}_{2}}\\ \mathrm{sin}{\alpha}_{{Th}_{2}}\mathrm{sin}{\beta}_{{Th}_{2}}\end{array}\right]+{F}_{4}\left[\begin{array}{c}\mathrm{sin}{\alpha}_{{Th}_{4}}\mathrm{cos}{\beta}_{{Th}_{4}}\\ \mathrm{sin}{\alpha}_{{Th}_{4}}\mathrm{sin}{\beta}_{{Th}_{4}}\\ \mathrm{cos}{\alpha}_{{Th}_{4}}\end{array}\right]+{F}_{6}\left[\begin{array}{c}\mathrm{sin}{\alpha}_{{Th}_{6}}\mathrm{cos}{\beta}_{{Th}_{6}}\\ -\mathrm{cos}{\alpha}_{{Th}_{6}}\\ \mathrm{sin}{\alpha}_{{Th}_{6}}\mathrm{sin}{\beta}_{{Th}_{6}}\end{array}\right]+{F}_{5}\left[\begin{array}{c}-\mathrm{cos}{\alpha}_{{Th}_{5}}\\ \mathrm{sin}{\alpha}_{{Th}_{5}}\mathrm{cos}{\beta}_{{Th}_{5}}\\ \mathrm{sin}{\alpha}_{{Th}_{5}}\mathrm{sin}{\beta}_{{Th}_{5}}\end{array}\right]+{F}_{3}\left[\begin{array}{c}\mathrm{sin}{\alpha}_{{Th}_{3}}\mathrm{cos}{\beta}_{{Th}_{3}}\\ \mathrm{sin}{\alpha}_{{Th}_{3}}\mathrm{sin}{\beta}_{{Th}_{3}}\\ -\mathrm{cos}{\alpha}_{{Th}_{3}}\end{array}\right]$$

where ${F}_{i}, i\in \left\{1,\dots , 6\right\}$ is the control force produced by thrusters. ${\alpha}_{{Th}_{i}}\,\,\epsilon\,\, \left[-\pi ,\pi \right]$ and ${\beta}_{{Th}_{i}}\,\,\epsilon\,\, \left[-\pi ,\pi \right]$ are the thrust misalignment angles shown in the Fig. 11 schematically. According to Fig. 4 the thruster’s misalignment for every 6 plates can be modeled as:

Based on the arm of the thrust vectors from the center of gravity of the chaser (shown in Fig. 12), the torque produced due to the thrusters’ misalignment can be calculated according to the Table 1:

**Table 1:** Thrusters' adverse (disturbing) torque.

Thruster	Incidence from normal of the plane (out of plane) axis	Incidence from in-plane	Adverse torque
1	${\alpha}_{{Th}_{1}}$	${\beta}_{{Th}_{1}}$	$${T}_{{F}_{1}}={\left[\begin{array}{lll}-\frac{L}{2}{F}_{{1}_{z}}& 0& \frac{L}{2}{F}_{{1}_{x}}\end{array}\right]}^{T}$$
2	${\alpha}_{{Th}_{2}}$	${\beta}_{{Th}_{2}}$	$${T}_{{F}_{2}}={\left[\begin{array}{lll}0& -\frac{L}{2}{F}_{{2}_{z}}& \frac{L}{2}{F}_{{2}_{y}}\end{array}\right]}^{T}$$
3	${\alpha}_{{Th}_{3}}$	${\beta}_{{Th}_{3}}$	$${T}_{{F}_{3}}={\left[\begin{array}{lll}\frac{L}{2}{F}_{{3}_{y}}& -\frac{L}{2}{F}_{{3}_{x}}& 0\end{array}\right]}^{T}$$
4	${\alpha}_{{Th}_{4}}$	${\beta}_{{Th}_{4}}$	$${T}_{{F}_{4}}={\left[\begin{array}{lll}-\frac{L}{2}{F}_{{4}_{y}}& \frac{L}{2}{F}_{{4}_{x}}& 0\end{array}\right]}^{T}$$
5	${\alpha}_{{Th}_{5}}$	${\beta}_{{Th}_{5}}$	$${T}_{{F}_{5}}={\left[\begin{array}{lll}0& \frac{L}{2}{F}_{{5}_{z}}& -\frac{L}{2}{F}_{{5}_{y}}\end{array}\right]}^{T}$$
6	${\alpha}_{{Th}_{6}}$	${\beta}_{{Th}_{6}}$	$${T}_{{F}_{6}}={\left[\begin{array}{lll}-\frac{L}{2}{F}_{{6}_{z}}& 0& \frac{L}{2}{F}_{{6}_{x}}\end{array}\right]}^{T}$$

Tilt angles of the thrust vectors are bounded ($\left|{\alpha}_{{Th}_{i}}\right|\le {\gamma}_{9}$ and $\left|{\beta}_{{Th}_{i}}\right|\le {\gamma}_{10}$).

Considering the thrust input uncertainty, Eq. (68) can be rewritten as:

69 $${F}_{c}={D}_{p}\left({F}_{Th}+{\widetilde{F}}_{Th}\right)$$

where, ${F}_{Th}={\left[{F}_{1}, {F}_{6}, {F}_{2}, {F}_{5}, {F}_{4}, {F}_{3}\right]}^{T}\,\,\epsilon\,\, {\mathfrak{R}}^{6\times 1}$ and ${\widetilde{F}}_{Th}={\left[{\widetilde{F}}_{1}, {\widetilde{F}}_{6}, {\widetilde{F}}_{2}, {\widetilde{F}}_{5}, {\widetilde{F}}_{4}, {\widetilde{F}}_{3}\right]}^{T}\,\,\epsilon\,\, {\mathfrak{R}}^{6\times 1}$ are the nominal/exact required force and uncertain input force vector, respectively. ${D}_{p}$ denotes the force distribution matrix which is defined as:

70 $${D}_{p}={{D}_{p}}_{0}+{{D}_{p}}_{1}$$

70a $${{D}_{p}}_{0}=\left[\begin{array}{ccc}0& 0& 1\\ 1& -1& 0\\ 0& 0& 0\end{array} \begin{array}{c}-1\\ 0\\ 0\end{array} \begin{array}{c}0\\ 0\\ 1\end{array} \begin{array}{c}0\\ 0\\ -1\end{array}\right]$$

70b $${{D}_{p}}_{1}=\left[\begin{array}{c}S{\alpha}_{{Th}_{1}} C{\beta}_{{Th}_{1}}\\ C{\alpha}_{{Th}_{1}}-1\\ S{\alpha}_{{Th}_{1}} S{\beta}_{{Th}_{1}}\end{array} \begin{array}{c}S{\alpha}_{{Th}_{6}} C{\beta}_{{Th}_{6}}\\ 1-C{\alpha}_{{Th}_{6}}\\ S{\alpha}_{{Th}_{6}} S{\beta}_{{Th}_{6}}\end{array} \begin{array}{c}C{\alpha}_{{Th}_{2}}-1\\ S{\alpha}_{{Th}_{2}} C{\beta}_{{Th}_{6}}\\ S{\alpha}_{{Th}_{2}} S{\beta}_{{Th}_{6}}\end{array} \begin{array}{c}1-C{\alpha}_{{Th}_{5}}\\ S{\alpha}_{{Th}_{5}} C{\beta}_{{Th}_{5}}\\ S{\alpha}_{{Th}_{5}} S{\beta}_{{Th}_{5}}\end{array} \begin{array}{c}S{\alpha}_{{Th}_{4}} C{\beta}_{{Th}_{4}}\\ S{\alpha}_{{Th}_{4}} S{\beta}_{{Th}_{4}}\\ C{\alpha}_{{Th}_{4}}-1\end{array} \begin{array}{c}S{\alpha}_{{Th}_{3}} C{\beta}_{{Th}_{3}}\\ S{\alpha}_{{Th}_{3}} S{\beta}_{{Th}_{3}}\\ 1-C{\alpha}_{{Th}_{3}}\end{array}\right]$$

${D}_{p}$, ${F}_{Th}$ and ${\widetilde{F}}_{Th}$ are bounded and ${\widetilde{F}}_{Th}\ll {F}_{Th}$.

The maximum (upper limit) adverse torque produced by thrusters which must be compensated by the attitude control subsystem is:

71 $${T}_{{d}_{Th}}=\frac{L}{2}\left[\begin{array}{c}{F}_{{1}_{z}}+{F}_{{6}_{z}}+{F}_{{4}_{y}}+{F}_{{3}_{y}}\\ {F}_{{2}_{z}}+{F}_{{5}_{z}}+{F}_{{3}_{x}}+{F}_{{4}_{x}}\\ {F}_{{1}_{x}}+{F}_{{6}_{x}}+{F}_{{2}_{y}}+{F}_{{5}_{y}}\end{array}\right]$$

The sliding mode control includes two parts as follows:

72 $${u}_{P}={u}_{{0}_{p}}+{u}_{{s}_{p}}$$

where ${\overrightarrow{u}}_{{0}_{P}}={\left[{u}_{{0}_{px}}, {u}_{{0}_{py}}, {u}_{{0}_{pz}}\right]}^{T}\,\,\epsilon\,\, {\mathfrak{R}}^{3\times 1}$ is a nonlinear feedback control input that is effective for the ideal system (in the absence of uncertainty and disturbances) that keeps the states on the sliding surface. ${\overrightarrow{u}}_{{s}_{P}}={\left[{u}_{{s}_{px}}, {u}_{{s}_{py}}, {u}_{{s}_{pz}}\right]}^{T}\,\,\epsilon\,\, {\mathfrak{R}}^{3\times 1}$ is the control input to deal with external disturbances and uncertainties.

The time-varying sliding surface is designed as follows^86^:

73 $${S}_{P}={C}_{P}{e}_{P}+Q\left(t\right)$$

where ${\overrightarrow{S}}_{P}={\left[{s}_{{p}_{x}},{s}_{{p}_{y}},{s}_{{p}_{z}}\right]}^{T}\,\,\epsilon\,\, {\mathfrak{R}}^{3\times 1}$ is the sliding surface, ${C}_{P}\,\,\epsilon\,\, {\mathfrak{R}}^{3\times 6}$ is a constant matrix that represents the slop of the sliding surface as follows:

74 $${C}_{P}=\left[{C}_{{p}_{1}},{C}_{{p}_{2}}\right]$$

where ${C}_{{p}_{1}}\,\,\epsilon\,\, {\mathfrak{R}}^{3\times 3}$ and ${C}_{{p}_{2}}\,\,\epsilon\,\, {\mathfrak{R}}^{3\times 3}$ are defined as:

75 $${C}_{{p}_{1}}=\left[\begin{array}{ccc}1& 0& 0\\ 0& 1& 0\\ 0& 0& 1\end{array}\right] , {C}_{{p}_{2}}=\left[\begin{array}{ccc}{c}_{{p}_{x}}& 0& 0\\ 0& {c}_{{p}_{y}}& 0\\ 0& 0& {c}_{{p}_{z}}\end{array}\right]$$

${e}_{P}\,\,\epsilon\,\, {\mathfrak{R}}^{6\times 1}$ Denotes the position and velocity error vector:

76 $${e}_{P}={\left[{e}_{{p}_{1}},{e}_{{p}_{2}}\right]}^{T}$$

77 $${e}_{{p}_{1}}=\left[{u}_{rel},{v}_{rel},{w}_{rel}\right] , {e}_{{p}_{2}}=\left[ {r}_{{rel}_{x}},{r}_{{rel}_{y}},{r}_{{rel}_{z}}\right]$$

$Q\left(t\right) \; \,\,\epsilon\,\, \;{\mathfrak{R}}^{3\times 1}$ is a quadratic polynomial that matches the states of the system on the sliding surface from the very beginning by eliminating the reaching phase. Eliminating the reaching phase will increase time response speed and start-up robustness. $Q\left(t\right)$ is given as follows:

78 $$Q\left(t\right)=\left\{\begin{array}{cc}{C}_{T}T& t\le {T}_{s}\\ 0& t>{T}_{s}\end{array}\right.$$

where ${C}_{T}=\left[{c}_{{T}_{1}},{c}_{{T}_{2}},{c}_{{T}_{3}}\right]\; \,\,\epsilon\,\, \;{\mathfrak{R}}^{3\times 3}$ is a constant matrix. ${c}_{{T}_{1}}$, ${c}_{{T}_{2}}$ and ${c}_{{T}_{3}}$ are defined as:

79 $${c}_{{T}_{1}}={\left[{c}_{{1}_{x}},{c}_{{1}_{y}},{c}_{{1}_{z}}\right]}^{T} \;\,\,\epsilon\,\,\; {\mathfrak{R}}^{3\times 1}$$

80 $${c}_{{T}_{2}}={\left[{c}_{{2}_{x}},{c}_{{2}_{y}},{c}_{{2}_{z}}\right]}^{T} \; \,\,\epsilon\,\,\; {\mathfrak{R}}^{3\times 1}$$

81 $${c}_{{T}_{3}}={\left[{c}_{{3}_{x}},{c}_{{3}_{y}},{c}_{{3}_{z}}\right]}^{T} \; \,\,\epsilon\,\, \;{\mathfrak{R}}^{3\times 1}$$

$T={\left[{t}^{2}, t, 1\right]}^{T} \; \,\,\epsilon\,\, \; {\mathfrak{R}}^{3\times 1}$ is the time vector.

Now we are going to find the ${c}_{{T}_{i}}, i=1, 2, 3$ vectors step by step. The following assumptions should be satisfied:

#### Assumption 1

The initial value of the system belongs to the sliding surface (presented in Eq. (73)), namely ${S}_{P}\left(0\right)=0$.

82 $${C}_{e}{e}_{P}+{c}_{{T}_{3}}=0$$

83 $${c}_{{T}_{3}}=\left[\begin{array}{c}{c}_{{3}_{x}}\\ {c}_{{3}_{y}}\\ {c}_{{3}_{z}}\end{array}\right]=-\left[\begin{array}{c}{c}_{x}{r}_{{rel}_{x}}(0)+{u}_{rel}(0)\\ {c}_{y}{r}_{{rel}_{y}}(0)+{v}_{rel}(0)\\ {c}_{z}{r}_{{rel}_{z}}(0)+{w}_{rel}(0)\end{array}\right]$$

where $\left[{r}_{{rel}_{x}}\left(0\right), {r}_{{rel}_{y}}\left(0\right), {r}_{{rel}_{z}}(0)\right]$ and $\left[{u}_{rel}(0),{v}_{rel}(0),{w}_{rel}(0)\right]$ are the position and velocity errors at the initial condition.

#### Assumption 2

The $Q\left(t\right)$ should be continuous and also its time derivative should be continuous at $t={T}_{s}$. Because there is no instantaneous oscillation in sliding surface or rapid change in error function at $t={T}_{s}$.

$Q\left(t\right)$ continuity condition is:

84 $${c}_{{t}_{1}}{T}_{s}^{2}+{c}_{{t}_{2}}{T}_{s}+{c}_{{t}_{3}}=0$$

$\frac{d}{dt}Q\left(t\right)$ Continuity condition is:

85 $$2{c}_{{t}_{1}}{T}_{s}+{c}_{{t}_{2}}=0$$

Combining Eqs. (83), (84), and Eq. (85) yields:

86 $${c}_{{T}_{1}}=\left[\begin{array}{c}{c}_{{1}_{x}}\\ {c}_{{1}_{y}}\\ {c}_{{1}_{z}}\end{array}\right]=\frac{-1}{{T}_{s}^{2}}\left[\begin{array}{c}{c}_{x}{r}_{{rel}_{x}}(0)+{u}_{rel}(0)\\ {c}_{y}{r}_{{rel}_{y}}(0)+{v}_{rel}(0)\\ {c}_{z}{r}_{{rel}_{z}}(0)+{w}_{rel}(0)\end{array}\right]\Rightarrow {c}_{{T}_{1}}=\frac{1}{{T}_{s}^{2}}{c}_{{T}_{3}}$$

87 $${c}_{{T}_{2}}=\left[\begin{array}{c}{c}_{{2}_{x}}\\ {c}_{{2}_{y}}\\ {c}_{{2}_{z}}\end{array}\right]=\frac{-2}{{T}_{s}}\left[\begin{array}{c}{c}_{x}{r}_{{rel}_{x}}(0)+{u}_{rel}(0)\\ {c}_{y}{r}_{{rel}_{y}}(0)+{v}_{rel}(0)\\ {c}_{z}{r}_{{rel}_{z}}(0)+{w}_{rel}(0)\end{array}\right]\Rightarrow {c}_{{T}_{2}}=\frac{-2}{{T}_{s}}{c}_{{T}_{3}}$$

There are currently no unknowns on the sliding surface (Eq. (73)). Thus, the controller can be derived now:

88 $${\dot{S}}_{P}={C}_{P}{\dot{e}}_{P}+\frac{d}{dt}Q\left(t\right)=0 \to {u}_{{0}_{c}}$$

89 $${\dot{S}}_{P}={C}_{{p}_{1}}{\dot{e}}_{{p}_{1}}+{C}_{{p}_{2}}{\dot{e}}_{{p}_{2}}+2{C}_{{T}_{1}}t+{C}_{{T}_{2}}=0$$

90 $${\dot{S}}_{P}={C}_{{p}_{1}}\left(\frac{{D}_{{p}_{0}}}{{m}_{c}}{u}_{{0}_{c}}-S\left({\omega}_{c}\right){\mathrm{v}}_{c}+S\left({\omega}_{rel}\right){R}_{T}^{c}{\mathrm{v}}_{T}+{R}_{T}^{c}S\left({\omega}_{T}\right){\mathrm{v}}_{T}\right)+{C}_{{p}_{2}}{\dot{\mathrm{r}}}_{rel}+2{{C}_{T}}_{1}t+{{C}_{T}}_{2}=0$$

Regardless of the disturbance forces $\left({F}_{{d}_{c}}, {F}_{{d}_{T}}\right)$, ${u}_{{0}_{c}}$ will be obtained as:

91 $${u}_{{0}_{c}}={D}_{{p}_{0}}^{ \dag}{m}_{c}\left({g}_{{p}_{1}}+{g}_{{p}_{2}}\right)$$

where ${D}_{{p}_{0}}^{ \dag}={D}_{{p}_{0}}^{T}{\left({D}_{{p}_{0}}{D}_{{p}_{0}}^{T}\right)}^{-1}$ denotes the pseudoinverse of ${D}_{{p}_{0}}$ matrix. ${g}_{{p}_{1}}$ and ${g}_{{p}_{2}}$ are defined as follows:

92 $${g}_{{p}_{1}}=S\left({\omega}_{c}\right){\mathrm{v}}_{c}$$

93 $${g}_{{p}_{2}}=-{R}_{T}^{c}\left(S\left({\omega}_{rel}\right)+S\left({\omega}_{T}\right)\right){\mathrm{v}}_{T}-{C}_{{p}_{1}}^{-1}\left({C}_{{p}_{2}}{\dot{\mathrm{r}}}_{rel}+2{{C}_{T}}_{1}t+{{C}_{T}}_{2}\right)$$

Know it’s time to calculate the ${u}_{{s}_{p}}$ to compensate for the thrusters’ misalignment, translational model disturbances, and uncertainties.

${u}_{{s}_{p}}$ is given as: $-{d}_{p}sign\left({S}_{P}\right)$ but ${\overrightarrow{d}}_{p}={\left[{d}_{{p}_{x}}, {d}_{{p}_{y}}, {d}_{{p}_{z}}\right]}^{T} \,\,\epsilon\,\, {\mathfrak{R}}^{3\times 1}$ should be calculated based on the Lyapunov stability theory. Thus, the candidate Lyapunov function is considered as:

94 $${V}_{p}=\frac{1}{2}{s}_{p}^{2}$$

Clearly, ${V}_{p}$ is positive definite, and its time derivative should be negative definite:

95 $${\dot{V}}_{p}={s}_{p}{\dot{s}}_{p}\le {\lambda}_{p}\left|{s}_{p}\right|$$

96 $${s}_{p}\left({\left(1+{\delta}_{{m}_{c}}\right)}^{-1}\left(\frac{{{D}_{p}}_{0}{u}_{{0}_{c}}+{{D}_{p}}_{1}{F}_{Th}+{D}_{p}{\widetilde{F}}_{Th}+{u}_{{s}_{p}}+{F}_{{d}_{c}}}{{m}_{c}}\right)-{\left(1+{\delta}_{{m}_{T}}\right)}^{-1}{R}_{T}^{c}\frac{{F}_{{d}_{T}}}{{m}_{T}}-S\left({\omega}_{c}\right){\mathrm{v}}_{c}+\left(\left(1+{\delta}_{{\omega}_{rel}}\right)S\left({\omega}_{rel}\right)+S\left({\omega}_{T}\right)\right){R}_{T}^{c}{\mathrm{v}}_{T}+\left(1+{\delta}_{{v}_{rel}}\right){C}_{{p}_{2}}{\dot{\mathrm{r}}}_{rel}+2{{C}_{T}}_{1}+{{C}_{T}}_{2}\le {\lambda}_{p}\left|{s}_{p}\right|\right)\le {\lambda}_{p}\left|{s}_{p}\right|$$

97 $${s}_{p}\left({{D}_{p}}_{0}{\left(1+{\delta}_{{m}_{c}}\right)}^{-1}\frac{{u}_{{0}_{c}}}{{m}_{c}}+{\left(1+{\delta}_{{m}_{c}}\right)}^{-1}\frac{\left({F}_{{d}_{c}}+{{D}_{p}}_{1}{F}_{Th}+{D}_{p}{\widetilde{F}}_{Th}\right)}{{m}_{c}}-{\left(1+{\delta}_{{m}_{c}}\right)}^{-1}\frac{{d}_{p}sign\left({S}_{P}\right)}{{m}_{c}}-{\left(1+{\delta}_{{m}_{T}}\right)}^{-1}{R}_{T}^{c}\frac{{F}_{{d}_{T}}}{{m}_{T}}-S\left({\omega}_{c}\right){\mathrm{v}}_{c}+\left(\left(1+{\delta}_{{\omega}_{rel}}\right)S\left({\omega}_{rel}\right)+S\left({\omega}_{T}\right)\right){R}_{T}^{c}{\mathrm{v}}_{T}+\left(1+{\delta}_{{v}_{rel}}\right){C}_{{p}_{2}}{\dot{\mathrm{r}}}_{rel}+2{{C}_{T}}_{1}+{{C}_{T}}_{2}\right)\le {\lambda}_{p}\left|{s}_{p}\right|$$

98 $${\left(1+{\delta}_{{m}_{c}}\right)}^{-1}\left(S\left({\omega}_{c}\right){\mathrm{v}}_{c}-{R}_{T}^{c}\left(S\left({\omega}_{rel}\right)+S\left({\omega}_{T}\right)\right){\mathrm{v}}_{T}-{C}_{{p}_{1}}^{-1}\left({C}_{{p}_{2}}{\dot{\mathrm{r}}}_{rel}+2{{C}_{T}}_{1}t+{{C}_{T}}_{2}\right)\right)+{\left(1+{\delta}_{{m}_{c}}\right)}^{-1}\frac{\left({F}_{{d}_{c}}+{{D}_{p}}_{1}{F}_{Th}+{D}_{p}{\widetilde{F}}_{Th}\right)}{{m}_{c}}-{\left(1+{\delta}_{{m}_{c}}\right)}^{-1}\frac{{d}_{p}sign\left({S}_{P}\right)}{{m}_{c}}-{\left(1+{\delta}_{{m}_{T}}\right)}^{-1}{R}_{T}^{c}\frac{{F}_{{d}_{T}}}{{m}_{T}}-S\left({\omega}_{c}\right){\mathrm{v}}_{c}+\left(\left(1+{\delta}_{{\omega}_{rel}}\right)S\left({\omega}_{rel}\right)+S\left({\omega}_{T}\right)\right){R}_{T}^{c}{\mathrm{v}}_{T}+\left(1+{\delta}_{{v}_{rel}}\right){C}_{{p}_{2}}{\dot{\mathrm{r}}}_{rel}+2{{C}_{T}}_{1}+{{C}_{T}}_{2}\le {\lambda}_{p}\left|{s}_{p}\right|$$

where ${\delta}_{{v}_{rel}}$ is the uncertainty of the linear relative velocity measured by relative navigation instruments.

By Substituting Eq. (91) in Eq. (98) the ${d}_{p}$ achieves as:

99 $${d}_{p}\ge \left(1+{\delta}_{{m}_{c}}\right){m}_{c}\left({\lambda}_{p}+\left|\left(\frac{-{\delta}_{{m}_{c}}}{1+{\delta}_{{m}_{c}}}\right)S\left({\omega}_{c}\right){\mathrm{v}}_{c}+\left(\frac{{\delta}_{{m}_{c}}+{\delta}_{{\omega}_{rel}}+{\delta}_{{m}_{c}}{\delta}_{{\omega}_{rel}}}{1+{\delta}_{{m}_{c}}}\right){R}_{T}^{c}S\left({\omega}_{rel}\right){\mathrm{v}}_{T}+\left(\frac{{\delta}_{{m}_{c}}}{1+{\delta}_{{m}_{c}}}\right){R}_{T}^{c}S\left({\omega}_{T}\right){\mathrm{v}}_{T}+\left(\left(1+{\delta}_{{v}_{rel}}\right)-{\left(1+{\delta}_{{m}_{c}}\right)}^{-1}{C}_{{p}_{1}}^{-1}\right)\left({C}_{{p}_{2}}{\dot{\mathrm{r}}}_{rel}\right)+\left(1-{\left(1+{\delta}_{{m}_{c}}\right)}^{-1}{C}_{{p}_{1}}^{-1}\right)\left(2{{C}_{T}}_{1}t+{{C}_{T}}_{2}\right)-{\left(1+{\delta}_{{m}_{T}}\right)}^{-1}{R}_{T}^{c}\frac{{F}_{{d}_{T}}}{{m}_{T}}+{\left(1+{\delta}_{{m}_{c}}\right)}^{-1}\frac{\left({F}_{{d}_{c}}+{{D}_{p}}_{1}{F}_{Th}+{D}_{p}{\widetilde{F}}_{Th}\right)}{{m}_{c}}\right|\right)$$

Notice that the signum function, which is utilized in ${{u}_{s}}_{a}$ and ${{u}_{s}}_{p}$ can cause chattering. Instead, the following saturation function can be used to resolve the issue:

100 $$sat\left( {\frac{s}{\varepsilon}} \right) = \left\{ {\begin{array}{*{20}l} 1 \hfill & {~if} \hfill & {~~s > \varepsilon} \hfill \\ {\frac{s}{\varepsilon}} \hfill & {~if} \hfill & { - \varepsilon \le s \le \varepsilon} \hfill \\ { - 1} \hfill & {~if} \hfill & {~s < - \varepsilon} \hfill \\ \end{array}} \right.$$

where $\varepsilon$ is the parameter of the saturation function that denotes the thickness of the boundary layer.

## Simulations and results analysis

In this section, a coupled six-degree-of-freedom motion simulation of a chaser spacecraft and a cooperative tumbling target is created to verify the effectiveness and robustness of the designed controller. The differential equations associated with the mathematical models of the chaser and target motion, controller, and disturbances discussed in previous sections are simulated (using a fixed-time-step Runge–Kutta integration method) with integration steps of 0.001 s.

Specifications of the target spacecraft and its orbital elements used for the simulation are given in Table 2. Specifications of the chaser spacecraft used for the simulation are given in Table 3.

**Table 2:** Target spacecraft specifications.

Target spacecraft specification	
Physical specifications	
Mass $\left({M}_{T}\right)$	$950 \left[\mathrm{kg}\right]$
Size	$1.65\times 1.75\times 1.85 \left[\mathrm{m}\right]$
Inertia $\left({I}_{T}\right)$	$diag\left(600, 500, 550\right) \left[{\mathrm{kgm}}^{2}\right]$
Orbit specifications	
Orbit type	Geocentric circular
Orbit altitude $\left({h}_{T}\right)$	$500 \left[\mathrm{km}\right]$
Orbit inclination $\left(i\right)$	$0^\circ$
Orbital velocity $\left({v}_{t}\right)$	$7.6127 \; [\text{km}/\text{s}]$

**Table 3:** Chaser spacecraft specifications.

Chaser spacecraft specification		
Physical specifications		
Mass $\left({M}_{c}\right)$		$100 \left[\mathrm{kg}\right]$
Size		$0.90\times 0.85\times 0.72 \left[\mathrm{m}\right]$
Inertia $\left({I}_{c}\right)$		$diag\left(11.5, 11.5, 11.5\right) \left[\mathrm{kg}.{\mathrm{m}}^{2}\right]$
Actuators specification		
Position control actuators:		6 thrusters
Each thruster mass $\left({m}_{thr}\right)$		0.38 [kg]
Fuel mass $\left({m}_{f}\right)$		15.7 [kg] (Hydrazine)
Specific impulse (${I}_{sp}$)		227 [s]
Thrusters force $\left({f}_{thr}\right)$		1 [N]
Thrusters misalignment	$\left[{\alpha}_{{Th}_{1}}, {\alpha}_{{Th}_{2}}, {\alpha}_{{Th}_{3}}\right]$	$\left[0.1, 0.1, 0.1\right] [\mathrm{deg}]$
	$\left[{\beta}_{{Th}_{1}}, {\beta}_{{Th}_{2}}, {\beta}_{{Th}_{3}}\right]$	$\left[0.2, 0.2, 0.2\right][\mathrm{deg}]$
Attitude control actuators:		3 orthogonal RWs
RWs mass		0.8 [kg]
Moment of inertia $\left({I}_{Rw}\right)$		$6.52\times {10}^{-4}\mathrm{ kg}{\mathrm{m}}^{2}$
Max Torque $\left({T}_{Rw}\right)$		0.02 [Nm]
Max Angular momentum $\left({h}_{Rw}\right)$		0.41 [Nms]
Max Angular velocity $\left({\omega}_{Rw}\right)$		6000 [RPM]
Delay		2 [ms]
Wheels misalignment	$\left[{\alpha}_{{RW}_{1}}, {\alpha}_{{RW}_{2}}, {\alpha}_{{RW}_{3}}\right]$	$\left[0.1, 0.1, 0.1\right] [\mathrm{deg}]$
	$\left[{\beta}_{{RW}_{1}}, {\beta}_{{RW}_{2}}, {\beta}_{{RW}_{3}}\right]$	$\left[0.2, 0.2, 0.2\right][\mathrm{deg}]$

Table 4 shows the values related to the effective parameters in calculating disturbances.

**Table 4:** Parameters of disturbances.

Disturbance torque	
Atmospheric drag	
Atmosphere density $(\rho @ 500 km)$	$6.967\times {10}^{-3}$ [kg/m^3^]
Drag area (S)	Target:$2 \left[{\mathrm{m}}^{2}\right]$
	Chaser: 0.77 $\left[{\mathrm{m}}^{2}\right]$
Drag coefficient $({C}_{D})$	2.2
ac position $({X}_{ac})$	Target:$0.01 [\mathrm{m}]$
	Chaser: $0.005 [\mathrm{m}]$
Atmosphere velocity $({v}_{atm})$	$0.5002$ [kg/s]
Solar radiation pressure	
Surface reflectance $(\mathrm{q})$	$0.6$
Solar constant $\left({S}_{ \odot}\right)$	1367 (w/m^2^)
Sun vector incidence angle $({i}_{s})$	$60^\circ$
cp position $({X}_{cp})$	$0.01 [\mathrm{m}]$
Solar Pressure area $\left({A}_{S}\right)$	$2 \left[{\mathrm{m}}^{2}\right]$
Magnetic disturbance	
Residual magnetic moment $\left(M\right)$	0.25 $\left[{\mathrm{Am}}^{2}\right]$
Magnetic moment (${\mu}_{\otimes}$)	$8.1\times {10}^{15} \left[{\mathrm{Tm}}^{3}\right]$
Magnetic latitude $\left(\mathsf{\Theta}\right)$	${12}^{^\circ}$
Geomagnetic flux density ($B$)	$2.5427\times {10}^{-5} [\mathrm{T}]$
Disturbance force	
J2 effect	
Earth radios (${R}_{E}$)	$6378 [\mathrm{km}]$
Standard gravitational parameter ($\mu$)	$398601.2 \; [{\mathrm{km}}^{2}/{\mathrm{s}}^{2}]$
Second zonal spherical harmonic $(J2)$	$0.00108263$

The gravity gradient disturbance torque is a function of the target spacecraft attitude and has a sinusoidal pattern (see Eq. 24), thus doesn’t calculate in the above table, but is considered in the simulation.

Table 5 represents the controller parameters used in the simulation.

**Table 5:** Controller parameters.

Controller parameters	
$\left[{c}_{{p}_{x}}, {c}_{{p}_{y}}, {c}_{{p}_{z}}\right]$	$\left[\mathrm{5,5},5\right]$
$\left[{\lambda}_{{p}_{x}}, {\lambda}_{{p}_{y}}, {\lambda}_{{p}_{z}}\right]$	$\left[\mathrm{0.01,0.01,0.01}\right]$
$\left[{k}_{{a}_{1}},{k}_{{a}_{2}},{k}_{{a}_{3}}\right]$	$\left[\mathrm{2,2},2\right]$
$\left[{k}_{{b}_{1}},{k}_{{b}_{2}},{k}_{{b}_{3}}\right]$	$\left[\mathrm{2,2},2\right]$
$\left[{\lambda}_{{A}_{x}}, {\lambda}_{{A}_{y}}, {\lambda}_{{A}_{z}}\right]$	$\left[\mathrm{0.01,0.01,0.01}\right]$
${T}_{s}$ [s]	10

The uncertainties bound considered in the simulations are presented in Table 6.

**Table 6:** Uncertainty amounts.

Control input uncertainty	${\widetilde{F}}_{\mathrm{Th}}=5\% {F}_{Th}$	${\widetilde{T}}_{\mathrm{RW}}=5\% {\mathrm{T}}_{\mathrm{RW}}$
Mass uncertainty	${\delta}_{{m}_{c}}=10\% {m}_{c}$	${\delta}_{{m}_{T}}=10\% {m}_{T}$
Inertia uncertainty	${\delta}_{{I}_{c}}=10\% {I}_{c}$	${\delta}_{{I}_{T}}=10\% {I}_{T}$
Measurement uncertainty:	${\delta}_{{\mathrm{v}}_{rel}}=5\% {\mathrm{v}}_{rel}$	${\delta}_{{\omega}_{rel}}=5\% {\omega}_{rel}$

**Figure 13:**
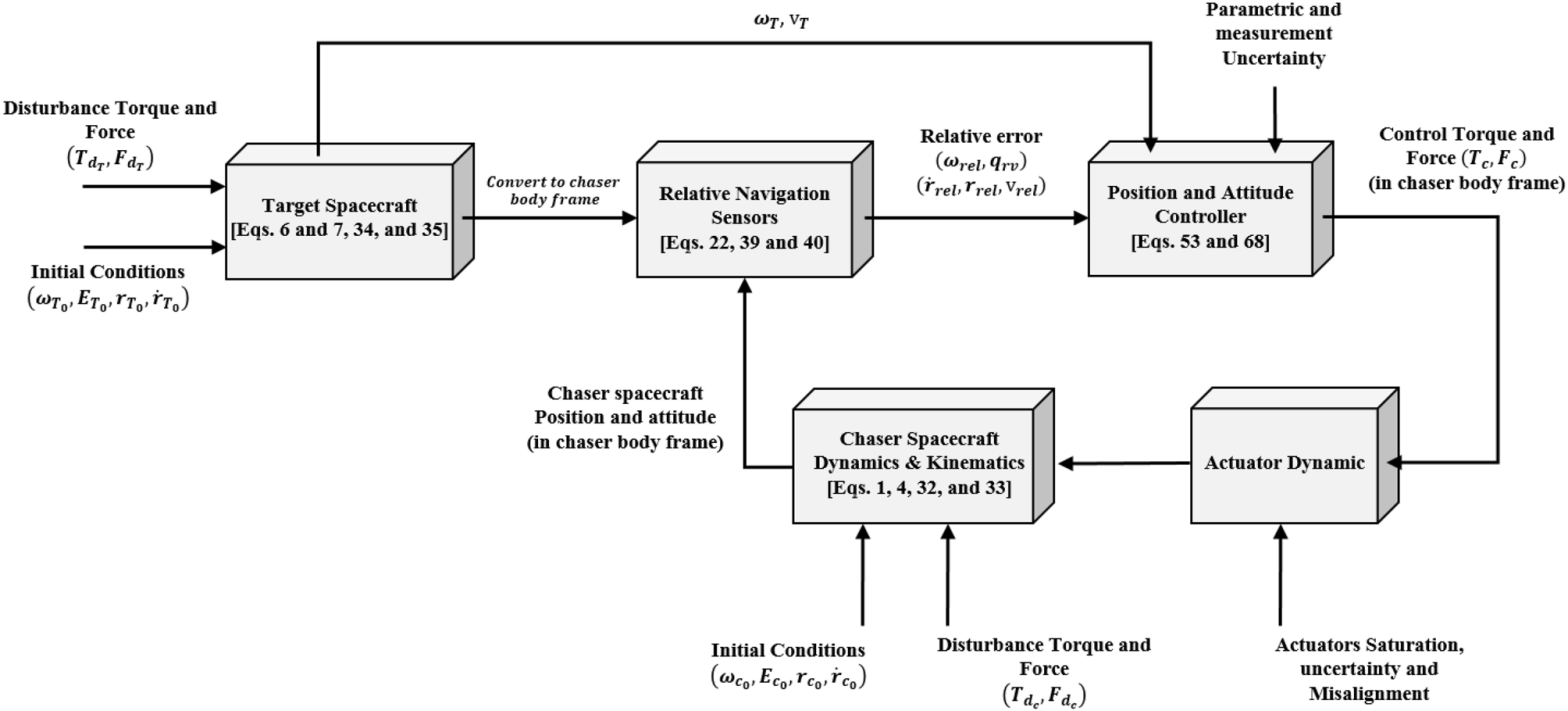
Block diagram architecture of the closed-loop system.

The block diagram architecture of the closed-loop system is shown in Fig. 13.

The simulation was done for three different scenarios, which will be discussed further.

**Scenario 1** In this scenario, the goal is to safe connection with the stable target moving in a circular orbit in nadir pointing condition. The initial values of the states are presented in Table 7.

**Table 7:** Chaser and target spacecraft initial conditions in scenario 1.

Initial relative position	${r}_{{rel}_{0}}={\left[\mathrm{20,50,50} \right]}^{T}$ $\left[\mathrm{m}\right]$
Initial relative velocity	${\dot{r}}_{{rel}_{0}}={\left[\mathrm{0,0},0 \right]}^{T}$ $\left[\mathrm{m}/\mathrm{s}\right]$
Target angular velocity	${\omega}_{T}={\left[\mathrm{0,0},0 \right]}^{T}$ $\left[\mathrm{deg}/\mathrm{s}\right]$
Target Euler angles	${E}_{T}={\left[\mathrm{0,0}, 0 \right]}^{T}$ $\left[\mathrm{deg}\right]$
Target’s initial velocity in body frame	${\mathrm{v}}_{T}=\left[- 0.003057, - 6.656, - 6878\right]$ $\left[\mathrm{km}/\mathrm{s}\right]$
Target’s initial position in body frame	${r}_{T}=\left[7.613, 0, 0\right]$ $\left[\mathrm{km}\right]$
Chaser initial angular velocity	${\omega}_{C}={\left[\mathrm{0,0},0 \right]}^{T}$ $\left[\mathrm{deg}/\mathrm{s}\right]$
Chaser initial Euler angles	${E}_{{c}_{0}}={\left[\mathrm{6,0}, 6 \right]}^{T}$ $\left[\mathrm{deg}\right]$
Chaser’s initial velocity in body frame	${\mathrm{v}}_{C}=\left[7.571, - 0.7914, 0.08318\right]$ $\left[\mathrm{km}/\mathrm{s}\right]$
Chaser’s initial position in body frame	${r}_{C}=\left[- 5.966e-15, - 719, - 6840\right] \left[\mathrm{km}\right]$
Disturbances	Not applied
Uncertainties	Not applied
RWs misalignment	Not applied

**Figure 14:**
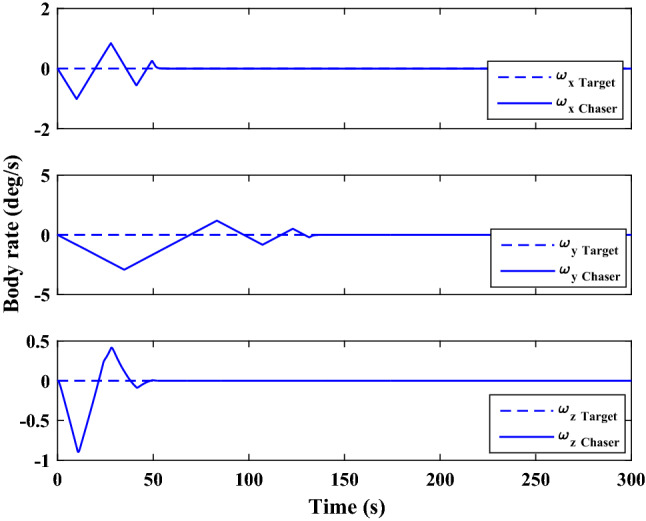
Scenario I: angular velocity tracking.

**Figure 15:**
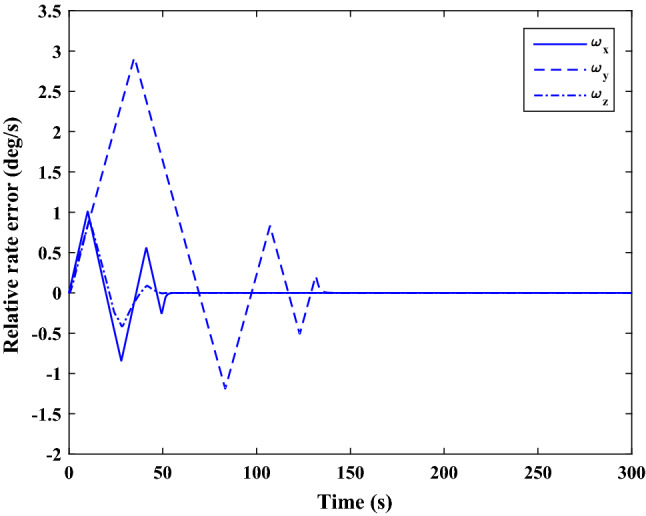
Scenario I: relative angular velocity error.

Figure 14 shows the target and chaser spacecraft angular velocity. The relative angular velocity error is depicted in Fig. 15.

**Figure 16:**
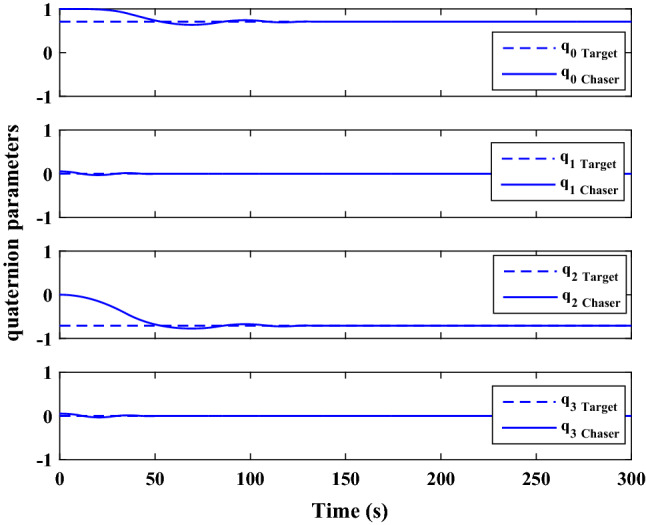
Scenario I: quaternion parameters tracking.

**Figure 17:**
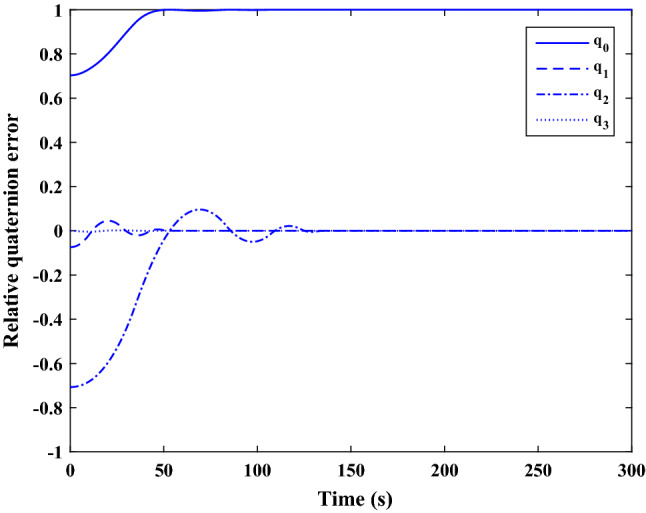
Scenario I: relative quaternion parameters error.

Figure 16 shows the target and chaser spacecraft quaternion parameters. The relative quaternion error is depicted in Fig. 17.

**Figure 18:**
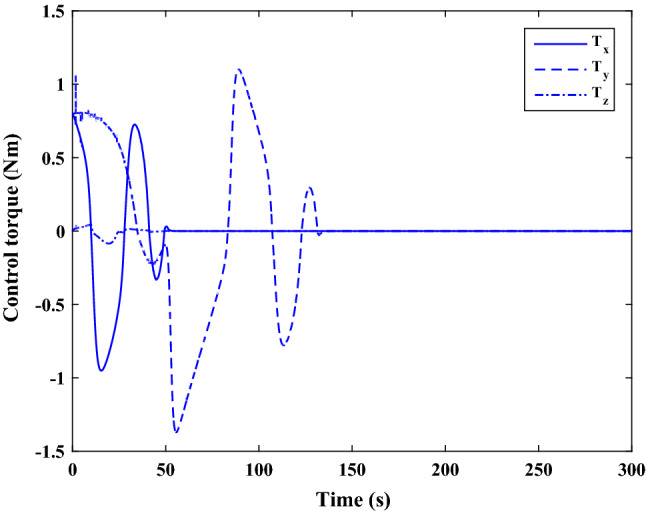
Scenario I: chaser control torque.

**Figure 19:**
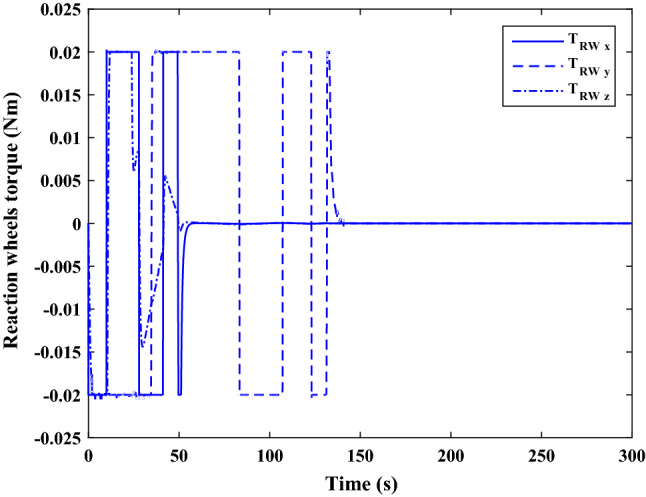
Scenario I: chaser’s RWs torque.

Figure 18 shows the control torque signal. The torque generated by RWs is depicted in Fig. 19.

**Figure 20:**
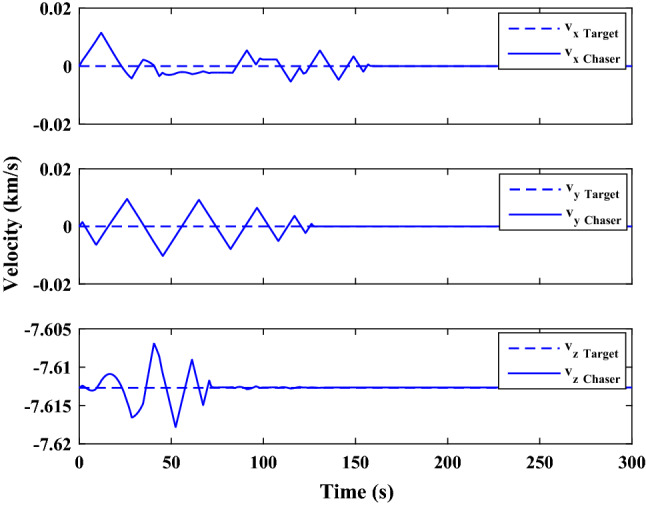
Scenario I: linear velocity tracking (in desired frame).

**Figure 21:**
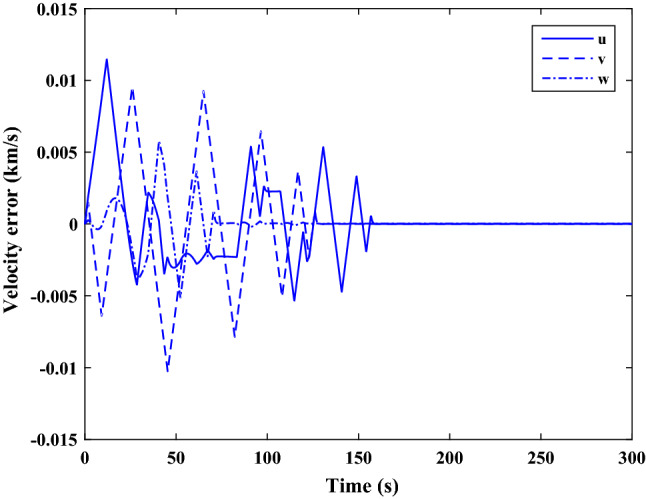
Scenario I: relative linear velocity error (in desired frame).

**Figure 22:**
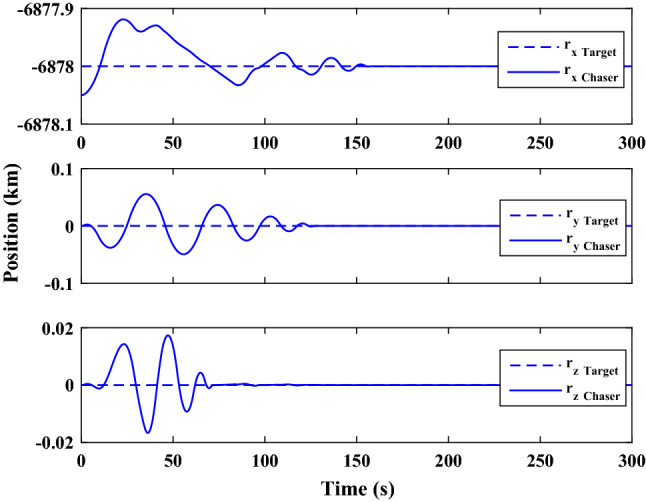
Scenario I: position tracking (in desired frame).

**Figure 23:**
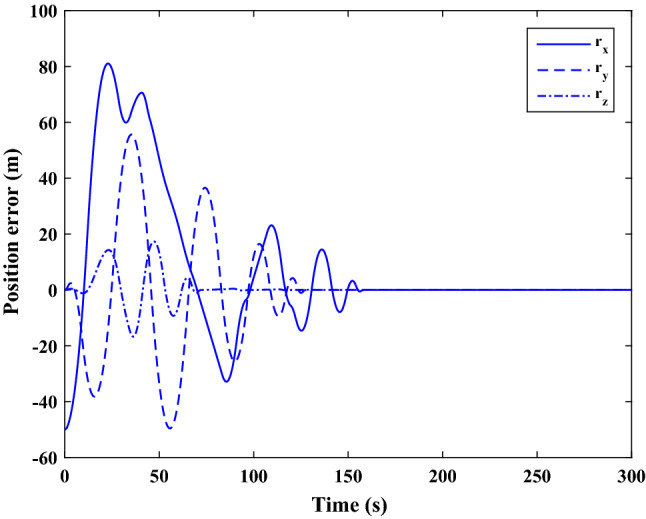
Scenario I: relative position error (in desired frame).

The linear velocity of the target and chaser spacecraft are plotted in Fig. 20. The relative linear velocity error is shown in Fig. 21. It should be noted that Figs. 20, 21, 22 and 23 are plotted in the desired frame.

The position of the target and chaser spacecraft are plotted in Fig. 22. The relative position error is shown in Fig. 23.

**Figure 24:**
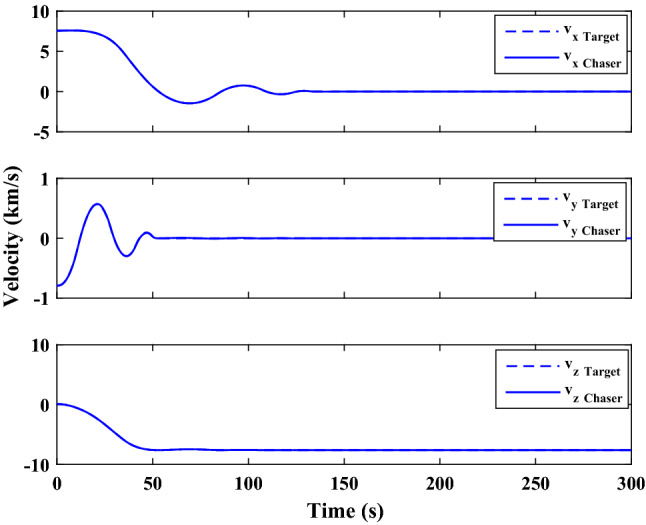
Scenario I: linear velocity tracking (in chaser body frame).

**Figure 25:**
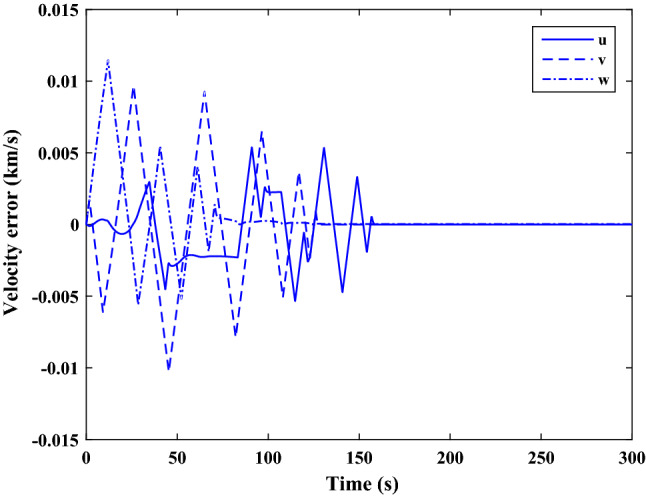
Scenario I: relative linear velocity error (in chaser body frame).

**Figure 26:**
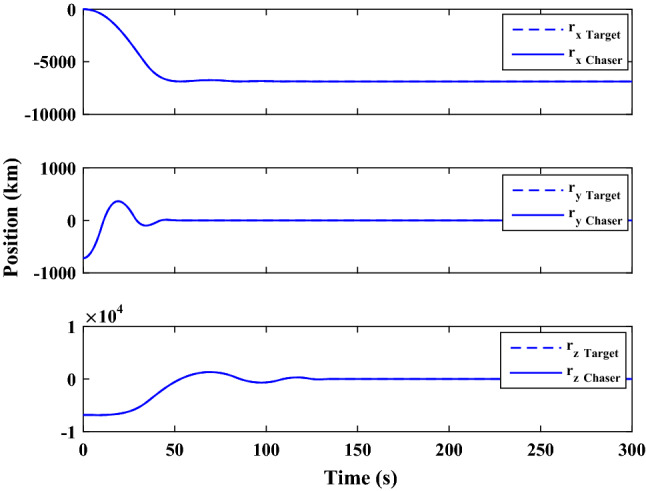
Scenario I: position tracking (in chaser body frame).

**Figure 27:**
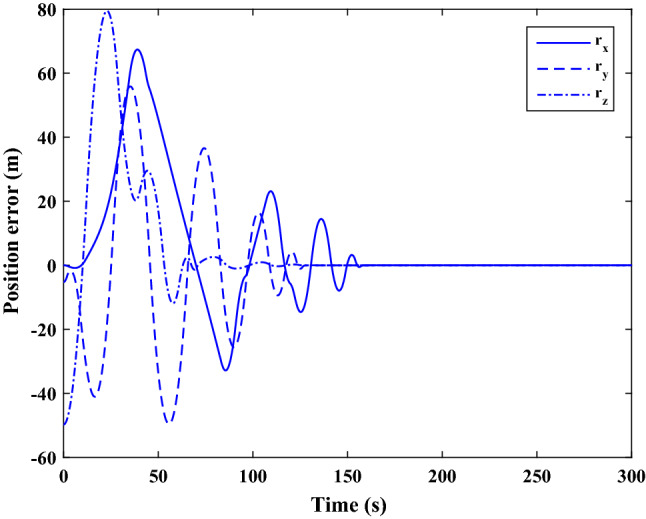
Scenario I: relative position error (in chaser body frame).

The linear velocity of the target and chaser spacecraft are plotted in Fig. 24. The relative linear velocity error is shown in Fig. 25. It should be noted that Figs. 24, 25, 26 and 27 are plotted in the chaser spacecraft body frame.

The position of the target and chaser spacecraft (measured in the chaser body frame) are plotted in Fig. 26, and The relative position error is shown in Fig. 27.

**Figure 28:**
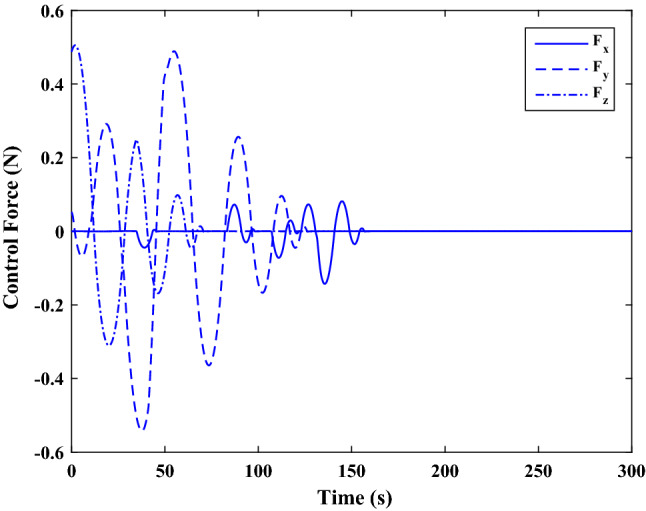
Scenario I: chaser control force.

**Figure 29:**
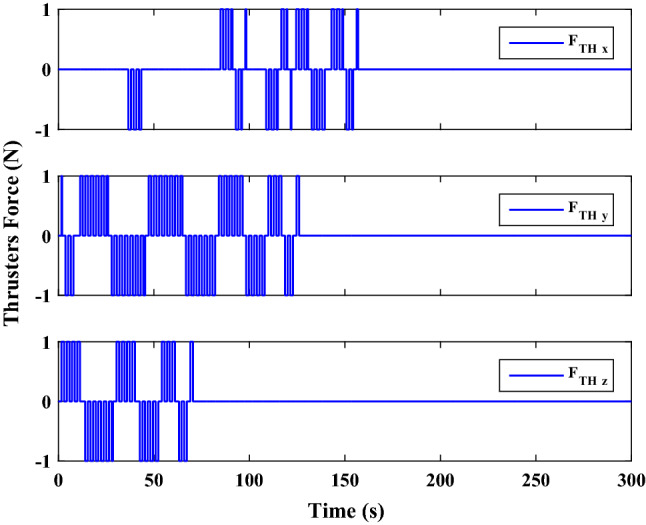
Scenario I: chaser’s thrusters force.

The control force and thruster forces are plotted in Figs. 28 and 29, respectively.

**Figure 30:**
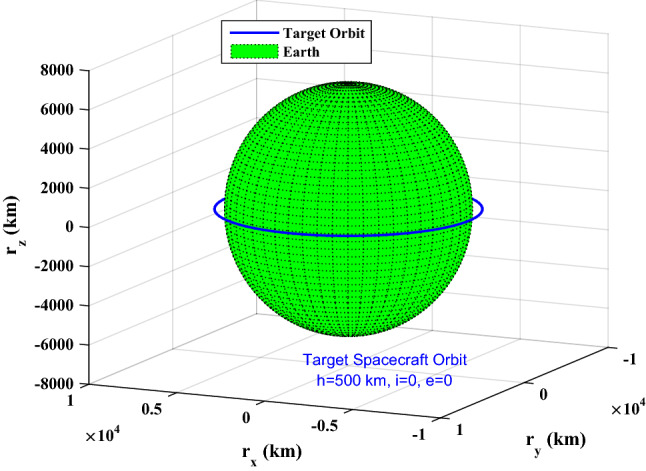
Scenario I: targets ideal orbit in the absence of external force.

**Figure 31:**
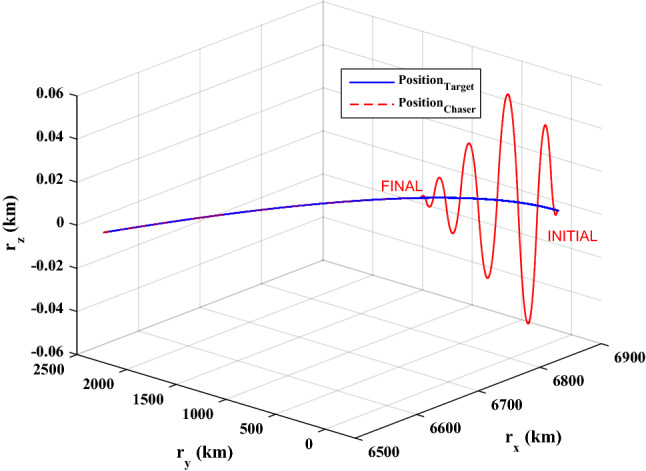
Scenario I: position of target and chaser spacecraft in ECI frame.

Figure 30 shows the target orbit in the presence of the earth in the ideal condition, and Fig. 31 presents the translational motion path of the chaser and target spacecraft in an ECI frame for 300 s.

As is evident in Figs. 14 and 31, the proximity operation is achieved within 160 s, and hereafter the pose motions of the two spacecraft keep synchronous.

**Figure 32:**
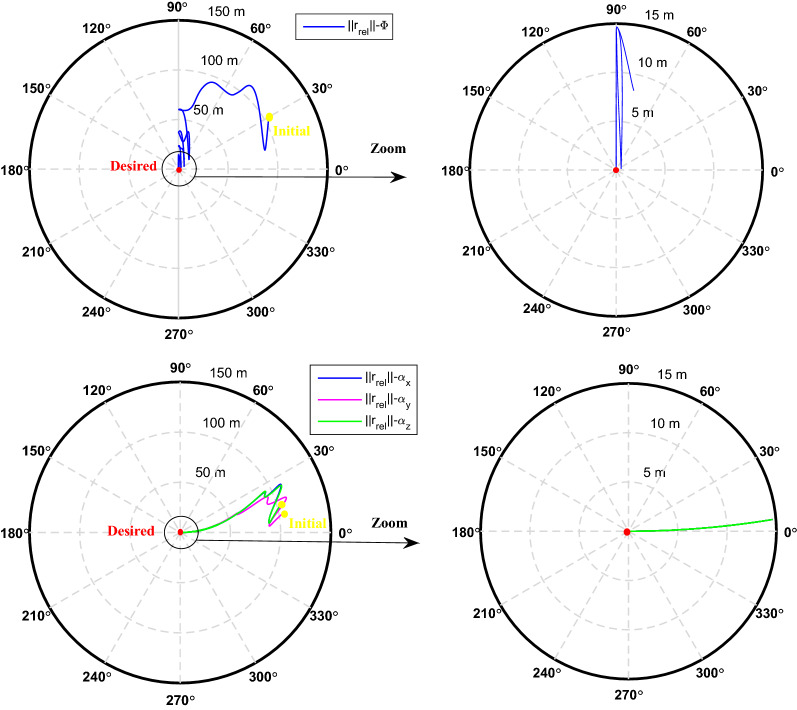
Relative pose trajectory in polar coordinate (Scenario 1).

Polar curves are provided in Fig. 32 to illustrate the trajectories of the relative pose motion. In these figures, $\Vert {r}_{rel}\Vert$ denotes the relative distance of two spacecraft (expressed in the desired frame). ${\alpha}_{x}, {\alpha}_{y}$ and ${\alpha}_{z}$ Are the angles that ${\overrightarrow{r}}_{rel}$ makes with the principal axes of the desired frame, relatively. $\Phi =2\mathrm{arccos}\left({q}_{{r}_{4}}\right)$ represents the rotation angle between the chaser body coordinate systems and the desired frame. the integrated position states in the polar coordinate $\left(\left[\Vert {r}_{rel}\Vert , {\alpha}_{x}, {\alpha}_{y}, {\alpha}_{z}\right]\right)$ describes the trajectory of the relative position vector $\left({\overrightarrow{r}}_{rel}\right)$ , while $\Vert {r}_{rel}\Vert$ and $\Phi$ denote the tracking process of, respectively, The relative translational and attitude motion convergence of the state ($\Vert {r}_{rel}\Vert$, $\Phi$) to the origin, shows that the attitude maneuver is achieved synchronously with the position tracking.

**Scenario 2** In this scenario, the goal is to safe connection with the uncontrolled target moving in a circular orbit. The point is that Tumbling is the result of external disturbance torques applied to the target. The initial values of the states are presented in Table 8.

**Table 8:** Chaser and target spacecraft initial conditions in scenario 2.

Initial relative position	${r}_{{rel}_{0}}={\left[\mathrm{20,50,50} \right]}^{T}$ $\left[\mathrm{m}\right]$
Initial relative velocity	${\dot{r}}_{{rel}_{0}}={\left[\mathrm{0,0},0 \right]}^{T}$ $\left[\mathrm{m}/\mathrm{s}\right]$
Target angular velocity	${\omega}_{T}={\left[\mathrm{0,0},0 \right]}^{T}$ $\left[\mathrm{deg}/\mathrm{s}\right]$
Target Euler angles	${E}_{T}={\left[\mathrm{0,0}, 0 \right]}^{T}$ $\left[\mathrm{deg}\right]$
Target’s initial velocity in body frame	${\mathrm{v}}_{T}=\left[- 0.003057, - 6.656, - 6878\right]$ $\left[\mathrm{km}/\mathrm{s}\right]$
Target’s initial position in body frame	${r}_{T}=\left[7.613, 0, 0\right]$ $\left[\mathrm{km}\right]$
Chaser initial angular velocity	${\omega}_{C}={\left[\mathrm{0,0},0 \right]}^{T}$ $\left[\mathrm{deg}/\mathrm{s}\right]$
Chaser initial Euler angles	${E}_{{c}_{0}}={\left[\mathrm{6,0}, 6 \right]}^{T}$ $\left[\mathrm{deg}\right]$
Chaser’s initial velocity in body frame	${\mathrm{v}}_{C}=\left[7.571, - 0.7914, 0.08318\right]$ $\left[\mathrm{km}/\mathrm{s}\right]$
chaser’s initial position in body frame	${r}_{C}=\left[- 5.966e-15, - 719, - 6840\right] \left[\mathrm{km}\right]$
Disturbances	Applied
Uncertainties	Applied
RWs misalignment	Applied

**Figure 33:**
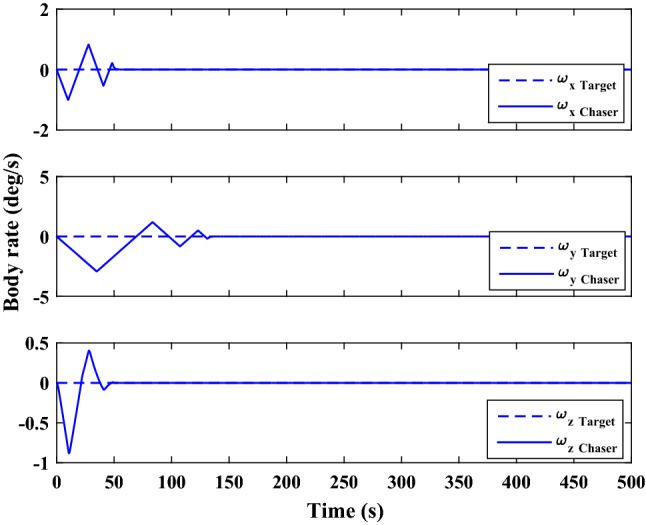
Scenario II: angular velocity tracking.

**Figure 34:**
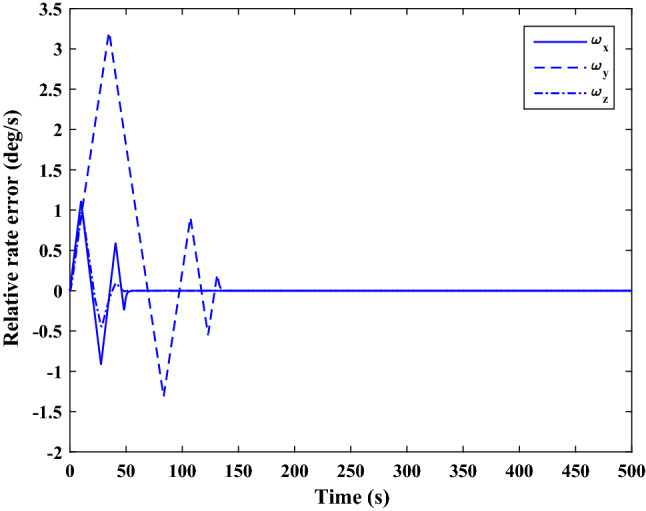
Scenario II: relative angular velocity error.

Figure 33 shows the target and chaser spacecraft angular velocity. The relative angular velocity error is depicted in Fig. 34.

**Figure 35:**
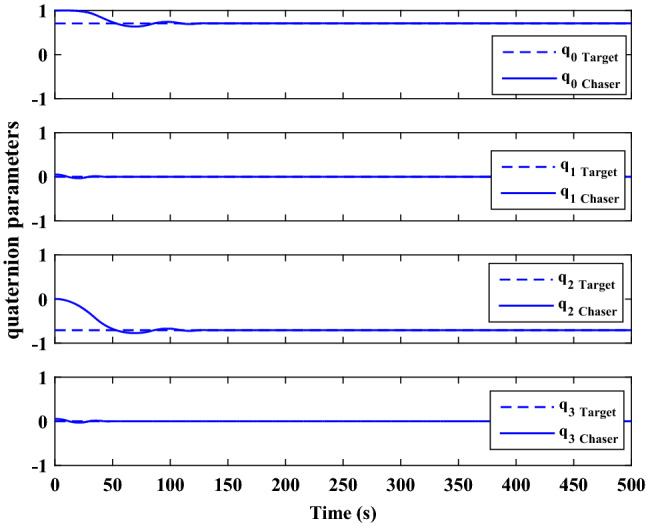
Scenario II: quaternion parameters tracking.

**Figure 36:**
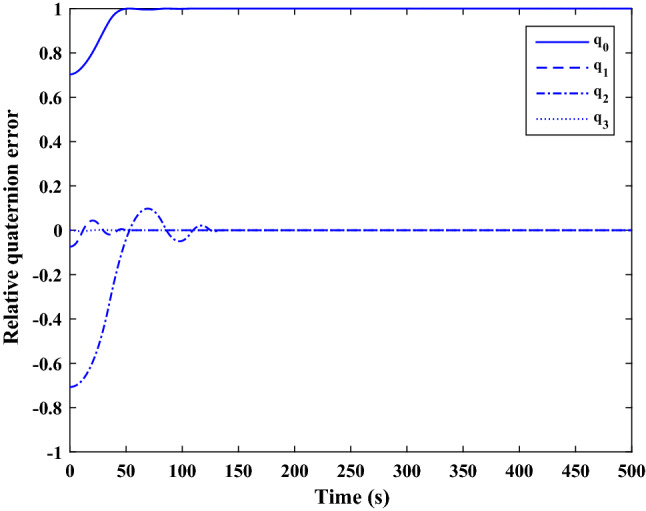
Scenario II: relative quaternion parameters error.

Figure 35 shows the target and chaser spacecraft quaternion parameters. The relative quaternion error is depicted in Fig. 36.

**Figure 37:**
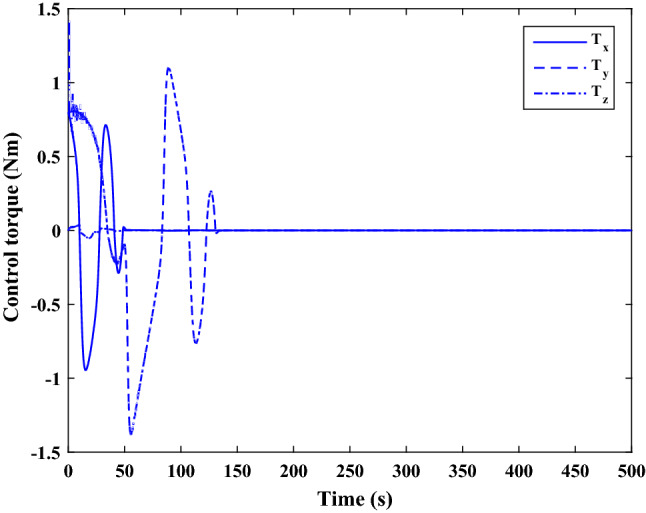
Scenario II: chaser control torque.

**Figure 38:**
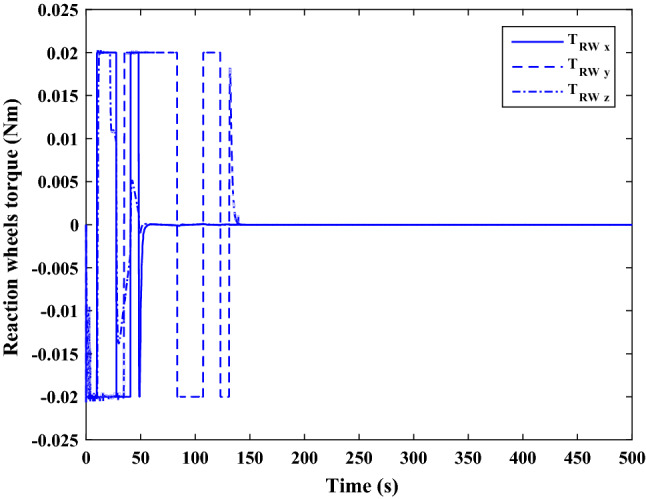
Scenario II: chaser’s RWs torque.

Figure 37 shows the control torque signal. The torque generated by RWs is depicted in Fig. 38.

**Figure 39:**
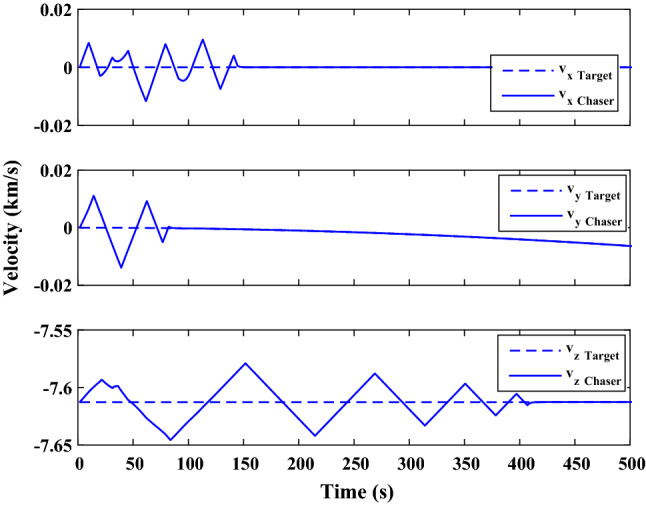
Scenario II: linear velocity tracking (in desired frame).

**Figure 40:**
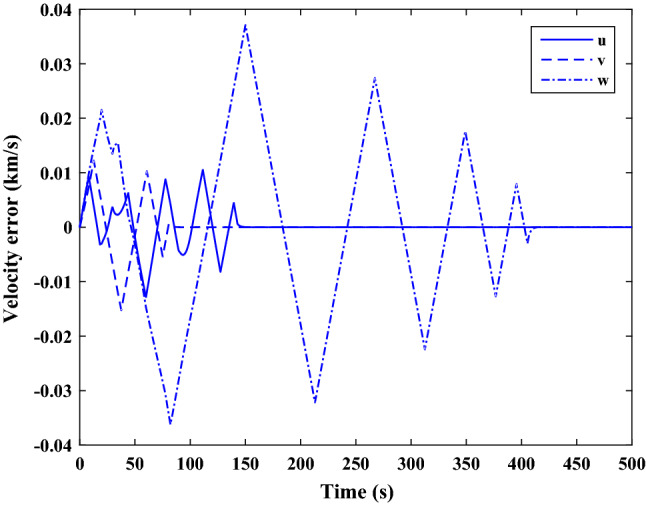
Scenario II: relative linear velocity error (in desired frame).

**Figure 41:**
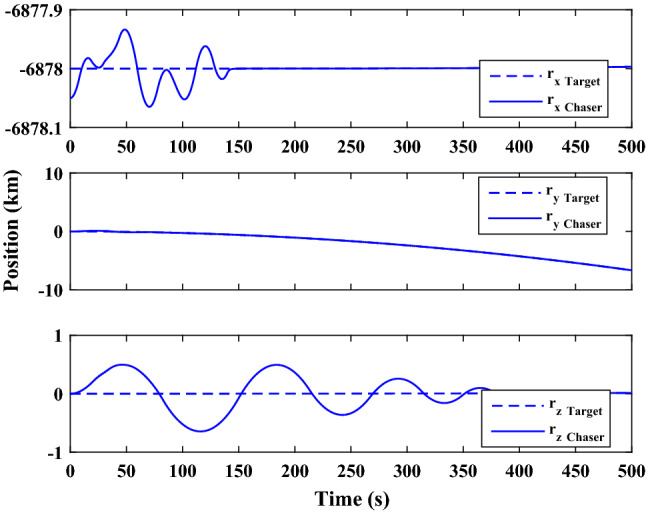
Scenario II: position tracking (in desired frame).

**Figure 42:**
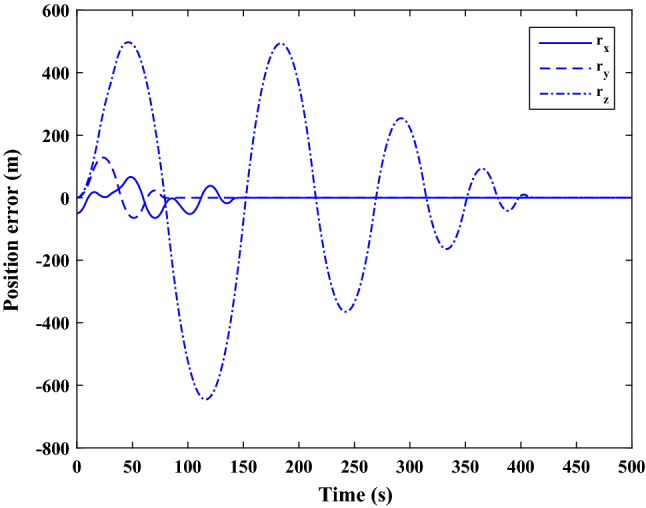
Scenario II: relative position error (in desired frame).

The linear velocity of the target and chaser spacecraft are plotted in Fig. 39. The relative linear velocity error is shown in Fig. 40. It should be noted that Figs. 39, 40, 41 and 42 are plotted in the desired frame.

The position of the target and chaser spacecraft are plotted in Fig. 41. The relative position error is shown in Fig. 42.

**Figure 43:**
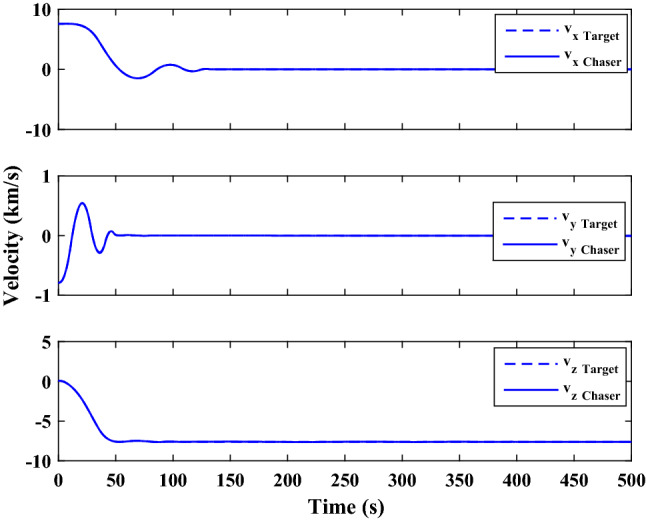
Scenario II: linear velocity tracking (in chaser body frame).

**Figure 44:**
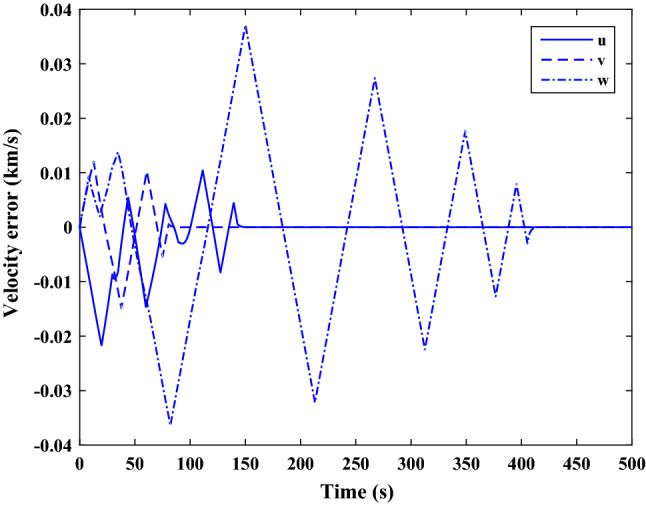
Scenario II: relative linear velocity error (in chaser body frame).

**Figure 45:**
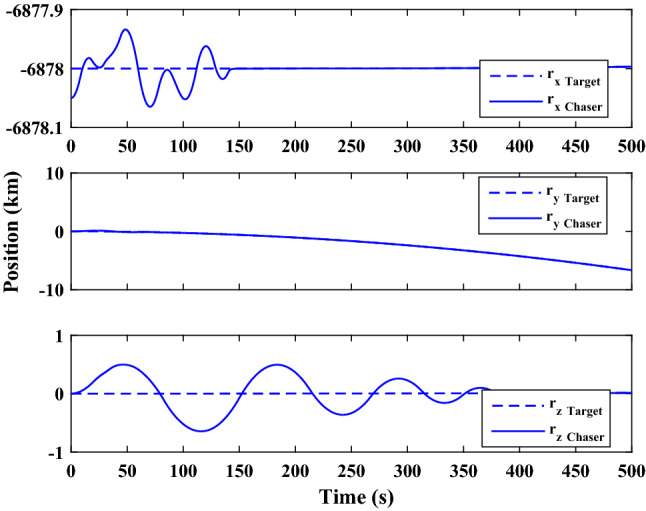
Scenario II: position tracking (in chaser body frame).

**Figure 46:**
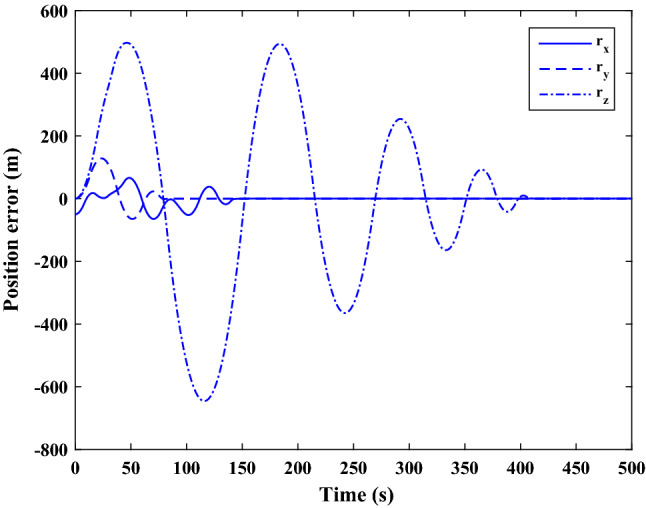
Scenario II: relative position error (in chaser body frame).

The linear velocity of the target and chaser spacecraft are plotted in Fig. 43. The relative linear velocity error is shown in Fig. 44. It should be noted that Figs. 43, 44, 45 and 46 are plotted in the chaser spacecraft body frame.

The position of the target and chaser spacecraft (measured in the chaser body frame) are plotted in Fig. 45, and The relative position error is shown in Fig. 46.

**Figure 47:**
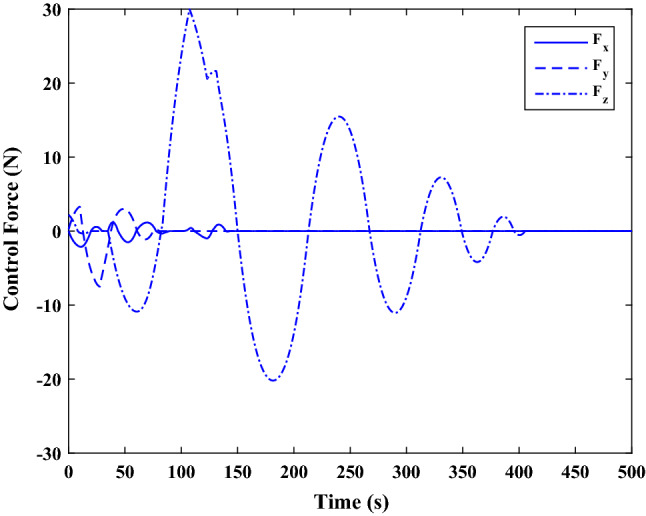
Scenario II: chaser control force.

**Figure 48:**
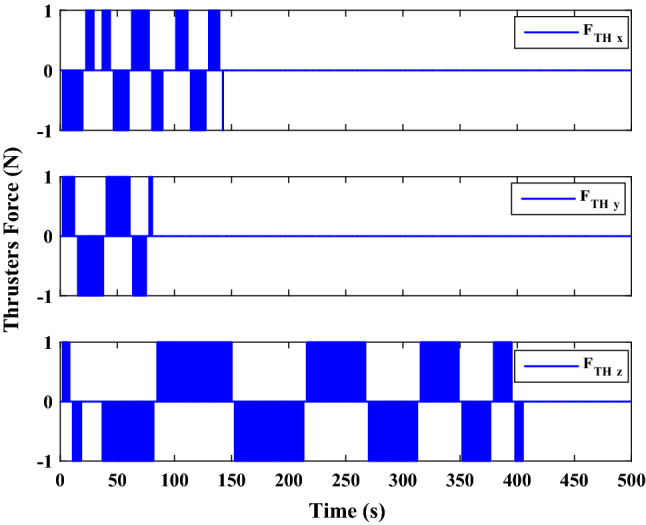
Scenario II: chaser’s thrusters force.

The control force and thruster forces are plotted in the Figs. 47 and 48, respectively.

**Figure 49:**
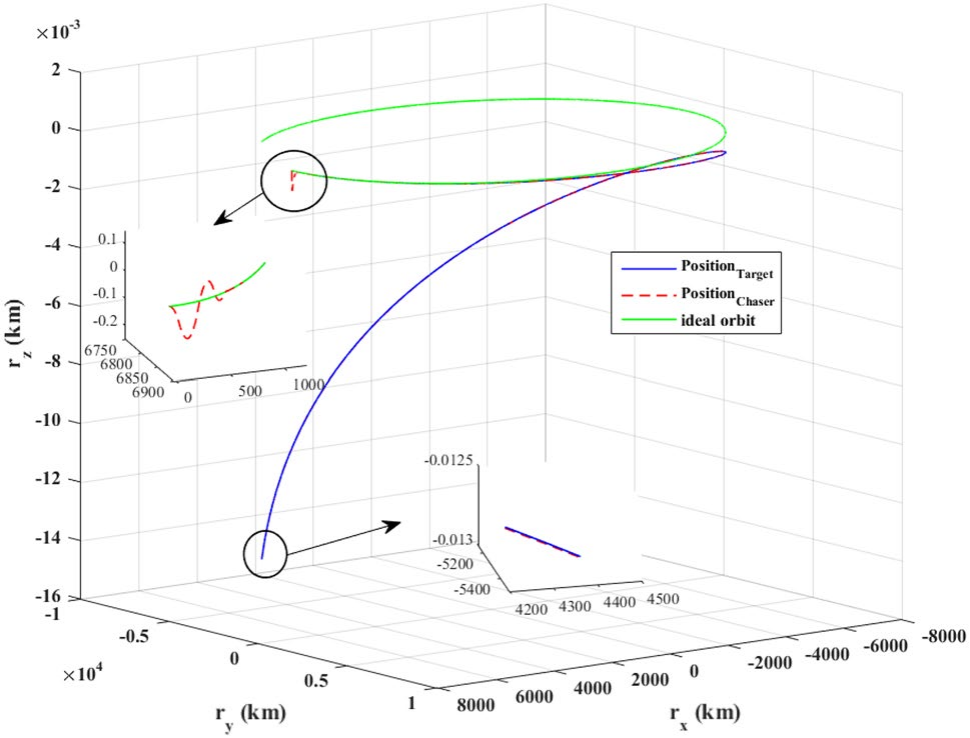
Scenario II: target’s and chaser’s motion path VS target’s ideal orbit (5000 s).

**Figure 50:**
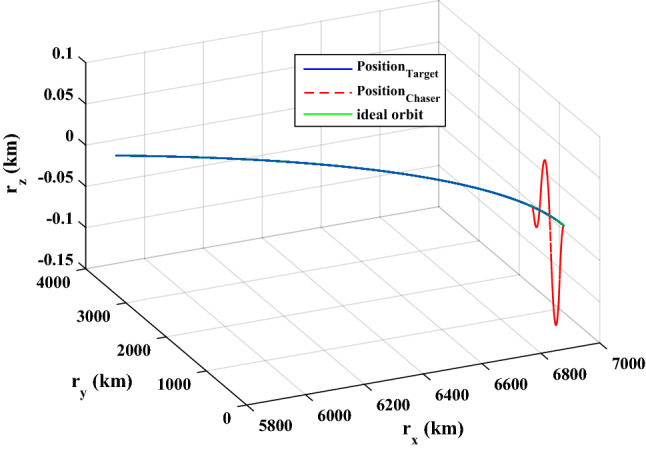
Scenario II: position of target and chaser spacecraft in ECI frame (500 s).

Figure 49 shows the target orbit in the presence of the ideal orbit (for 5000 s), and Fig. 50 presents the translational motion path of the chaser and target spacecraft in an ECI frame for 500 s.

Comparing Figs. 30 and 49, it can be concluded that the presence of disturbance torques and forces caused the target spacecraft's path to deviate from its ideal orbit.

**Figure 51:**
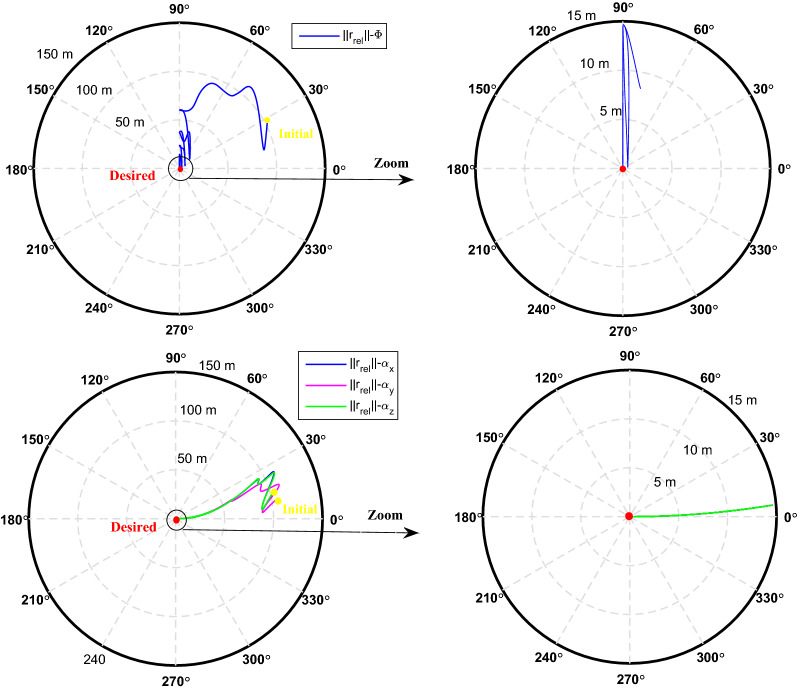
Relative pose trajectory in polar coordinate (Scenario 2).

The polar curve plotted in Fig. 51 corresponds to the integrated relative pose trajectory.

**Scenario 3** In this scenario, the goal is to safe connection with the tumbling target moving in a circular orbit. In this case, Tumbling is the result of both external disturbance toques and initial angular velocity. The initial values of the states are presented in Table 9.

**Table 9:** Chaser and target spacecraft initial conditions in scenario 3.

Initial relative position	${r}_{{rel}_{0}}={\left[\mathrm{20,50,50} \right]}^{T}$ $\left[\mathrm{m}\right]$
Initial relative velocity	${\dot{r}}_{{rel}_{0}}={\left[\mathrm{0,0},0 \right]}^{T}$ $\left[\mathrm{m}/\mathrm{s}\right]$
Target angular velocity	${\omega}_{T}={\left[\mathrm{1,0},0 \right]}^{T}$ $\left[\mathrm{deg}/\mathrm{s}\right]$
Target Euler angles	${E}_{T}={\left[\mathrm{0,0}, 5 \right]}^{T}$ $\left[\mathrm{deg}\right]$
Target’s initial velocity in body frame	${\mathrm{v}}_{T}=\left[7.5837, 0.5088, 0.4261\right]$ $\left[\mathrm{km}/\mathrm{s}\right]$
Target’s initial position in body frame	${r}_{T}=\left[- 1.3294, - 4403.9676, 5283.1763\right]$ $\left[\mathrm{km}\right]$
Chaser initial angular velocity	${\omega}_{C}={\left[\mathrm{0,0},0 \right]}^{T}$ $\left[\mathrm{deg}/\mathrm{s}\right]$
Chaser initial Euler angles	${E}_{{c}_{0}}={\left[\mathrm{0,0}, 0 \right]}^{T}$ $\left[\mathrm{deg}\right]$
Chaser’s initial velocity in body frame	${\mathrm{v}}_{C}=\left[\mathrm{7.6126635588682,0},0\right]$ $\left[\mathrm{km}/\mathrm{s}\right]$
Chaser’s initial position in body frame	${r}_{C}=\left[\mathrm{0,0},- 6878.0500002108\right] \left[\mathrm{km}\right]$
Disturbances	Applied
Uncertainties	Applied
RWs misalignment	Applied

**Figure 52:**
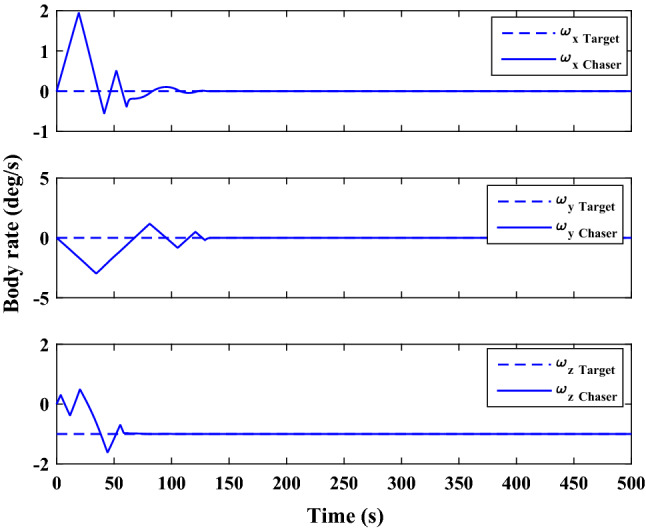
Scenario III: angular velocity tracking.

**Figure 53:**
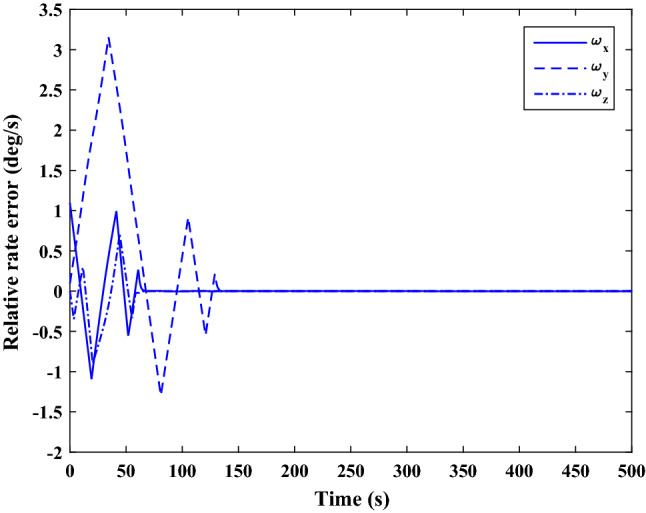
Scenario III: relative angular velocity error.

Figure 52 shows the target and chaser spacecraft angular velocity. The relative angular velocity error is depicted in Fig. 53.

**Figure 54:**
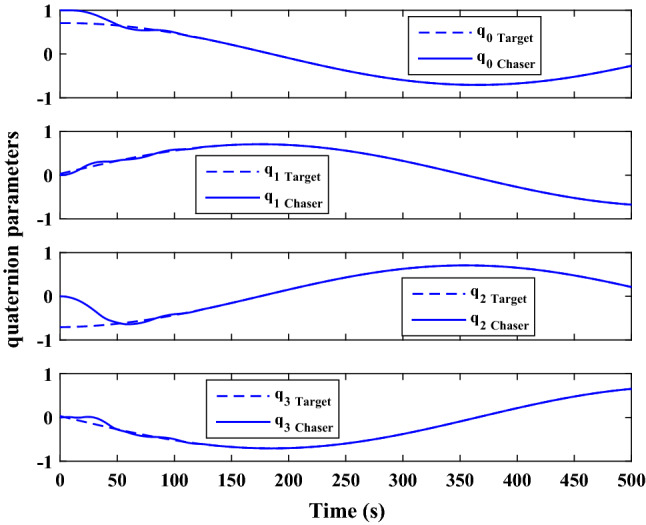
Scenario III: quaternion parameters tracking.

**Figure 55:**
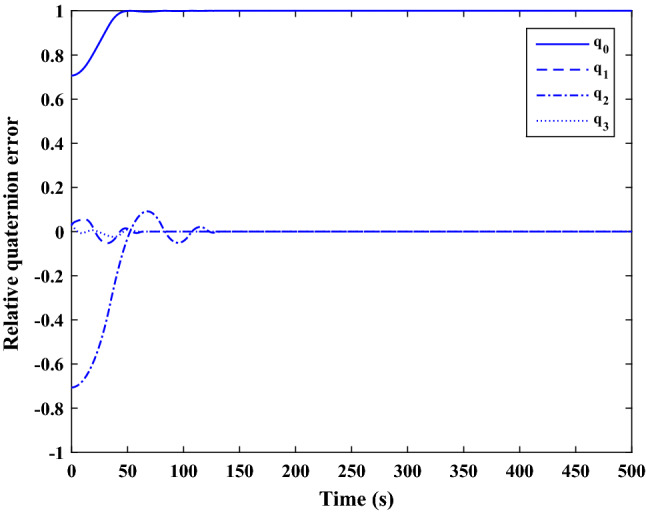
Scenario III: relative quaternion parameters error.

Figure 54 shows the target and chaser spacecraft quaternion parameters. The relative quaternion error is depicted in Fig. 55.

**Figure 56:**
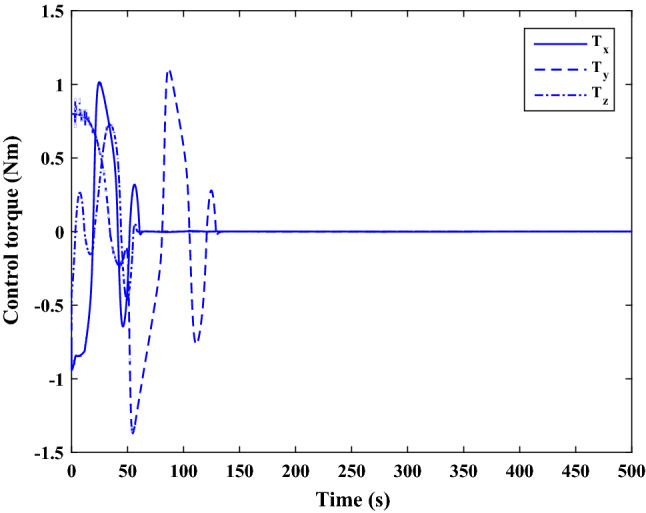
Scenario III: chaser control torque.

**Figure 57:**
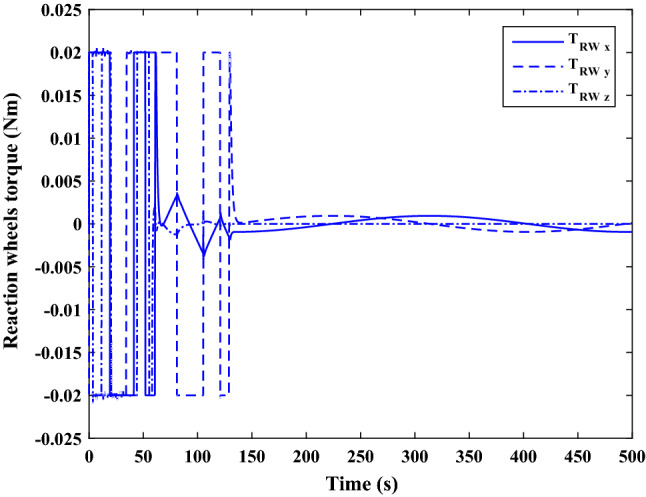
Scenario III: chaser’s RWs torque.

Figure 56 shows the control torque signal. The torque generated by RWs is depicted in Fig. 57.

**Figure 58:**
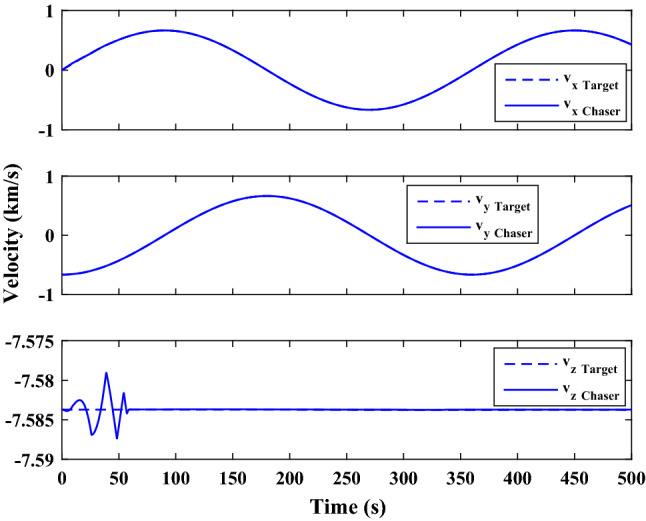
Scenario III: linear velocity tracking (in desired frame).

**Figure 59:**
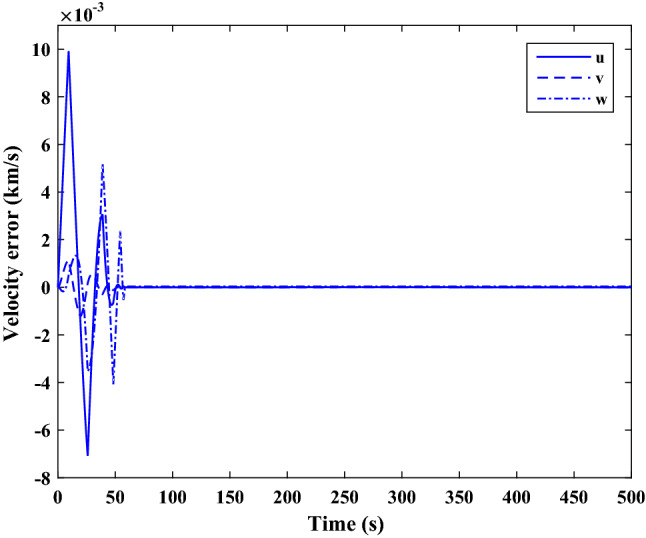
Scenario III: relative linear velocity error (in desired frame).

**Figure 60:**
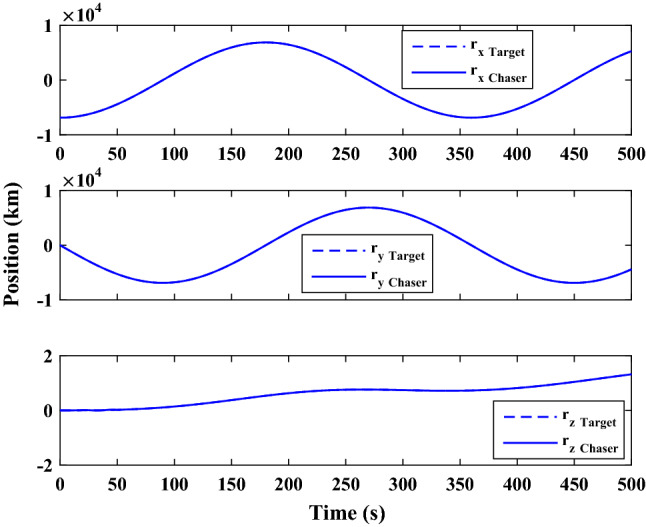
Scenario III: position tracking (in desired frame).

**Figure 61:**
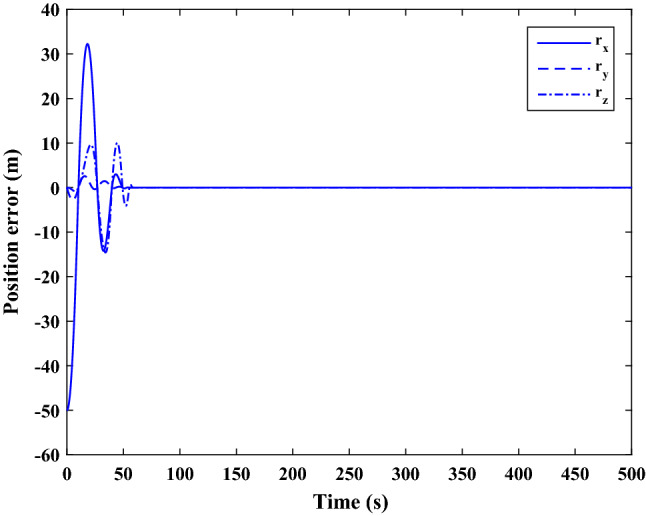
Scenario III: relative position error (in desired frame).

The linear velocity of the target and chaser spacecraft are plotted in Fig. 58. The relative linear velocity error is shown in Fig. 59. It should be noted that Figs. 58, 59, 60 and 61 are plotted in the desired frame.

The position of the target and chaser spacecraft are plotted in Fig. 60. The relative position error is shown in Fig. 61.

**Figure 62:**
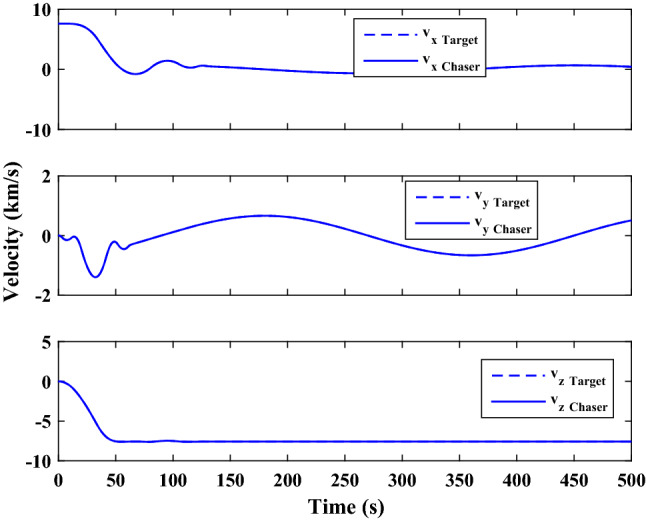
Scenario III: linear velocity tracking (in chaser body frame).

**Figure 63:**
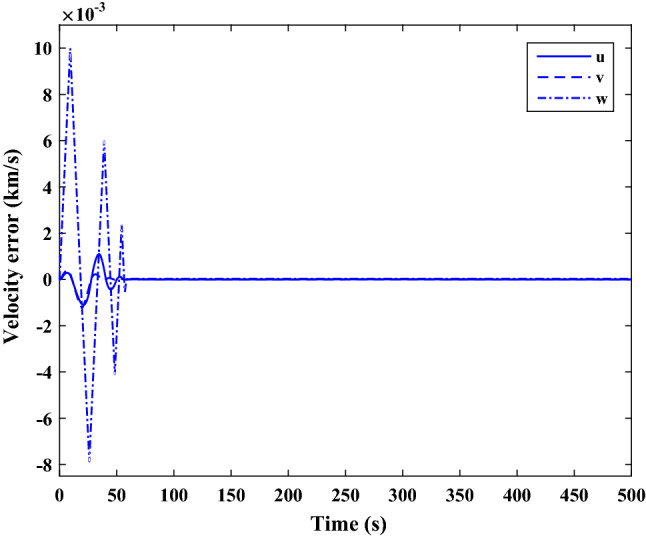
Scenario III: relative linear velocity error (in chaser body frame).

**Figure 64:**
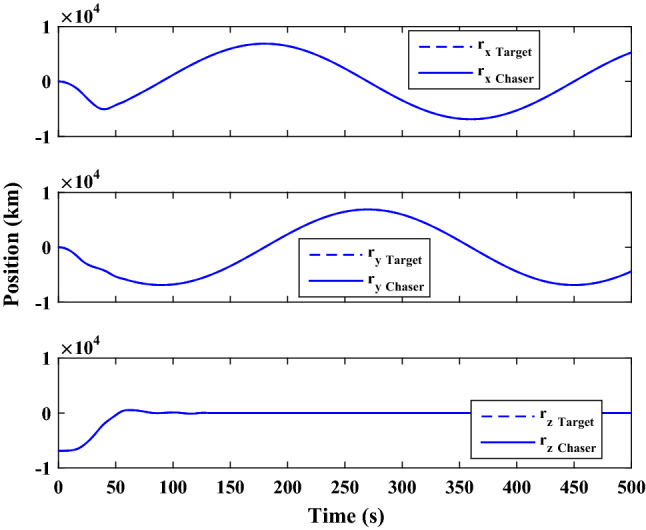
Scenario III: position tracking (in chaser body frame).

**Figure 65:**
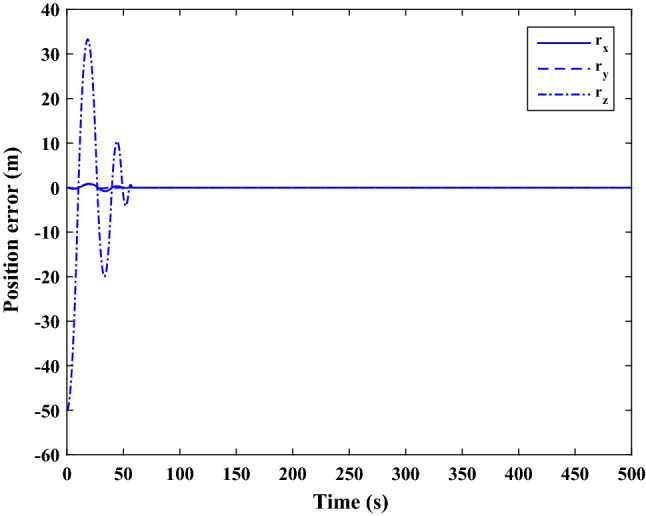
Scenario III: relative position error (in chaser body frame).

The linear velocity of the target and chaser spacecraft are plotted in Fig. 62. The relative linear velocity error is shown in Fig. 63. It should be noted that Figs. 62, 63, 64 and 65 are plotted in the chaser spacecraft body frame.

The position of the target and chaser spacecraft (measured in the chaser body frame) are plotted in Fig. 64, and The relative position error is shown in Fig. 65.

**Figure 66:**
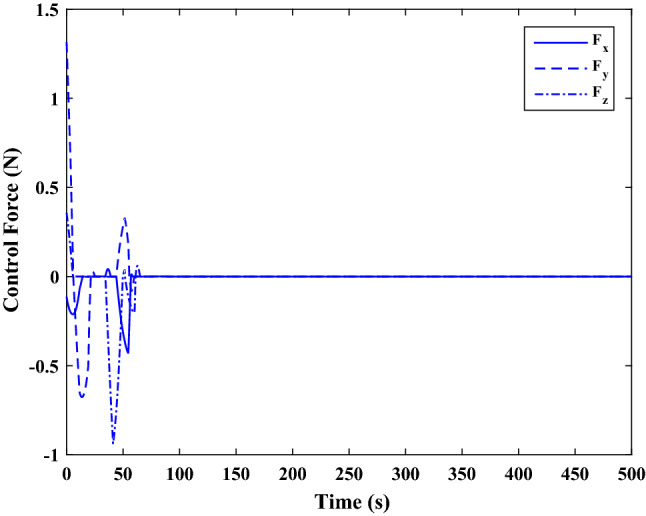
Scenario III: chaser control force.

**Figure 67:**
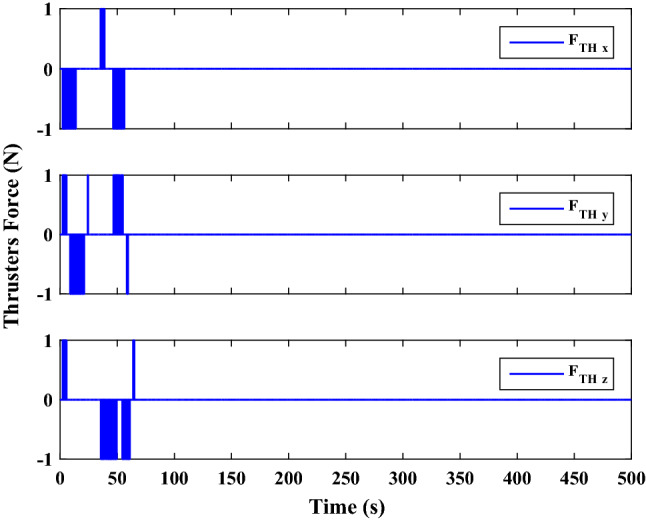
Scenario III: chaser’s thrusters force.

The control force and thruster forces are plotted in Figs. 66 and 67, respectively.

**Figure 68:**
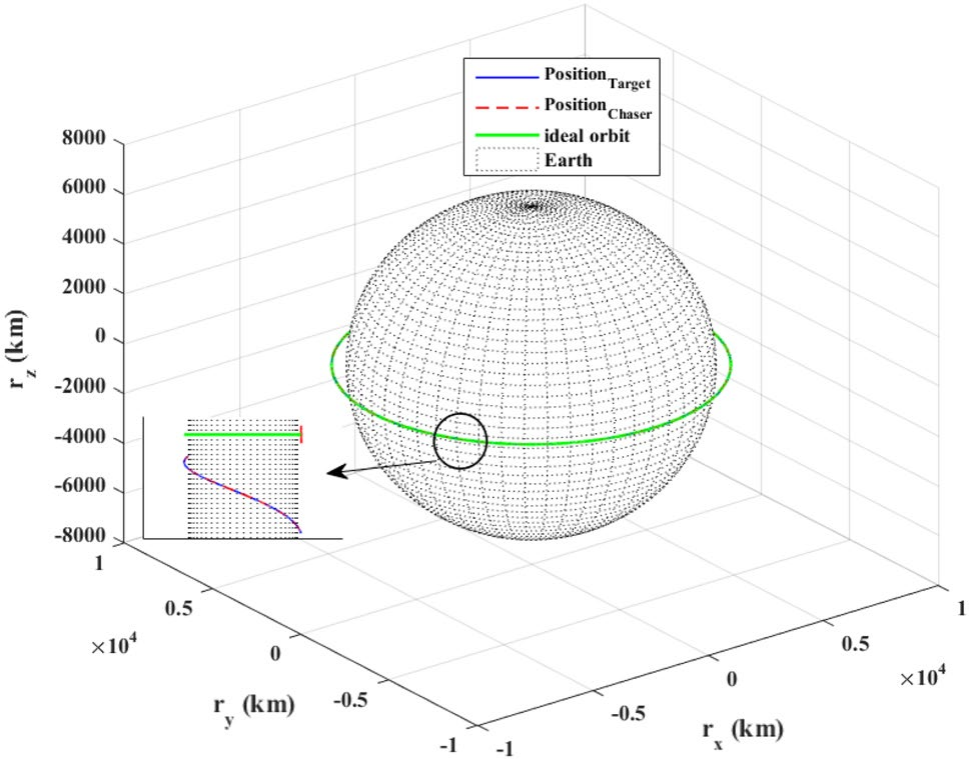
Scenario III: target’s and chaser’s motion path VS target’s ideal orbit (5000 s).

**Figure 69:**
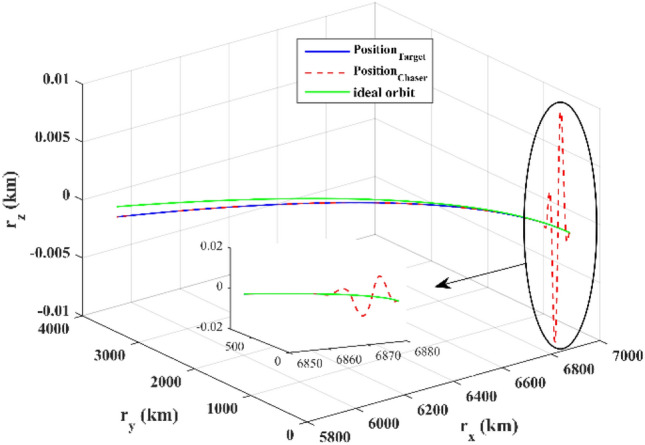
Scenario III: position of target and chaser spacecraft in ECI frame (500 s).

Figure 68 shows the target and chaser path in the presence of the ideal orbit and earth (for 5000 s), and Fig. 69 presents the translational motion path of the chaser and target spacecraft in an ECI frame for 500 s.

According to the simulation results shown in 14, 15, 16, 17, 18, 19, 20, 21, 22, 23, 24, 25, 26, 27, 28, 29, 30, 31, 32, 33, 34, 35, 36, 37, 38, 39, 40, 41, 42, 43, 45, 46, 47, 48, 49, 50, 51, 52, 53, 54, 55, 56, 57, 58, 59, 60, 61, 62, 63, 64, 65, 66, 67, 68 and 69, it can be claimed the designed controllers were successful in their mission to pursue the target.

**Figure 70:**
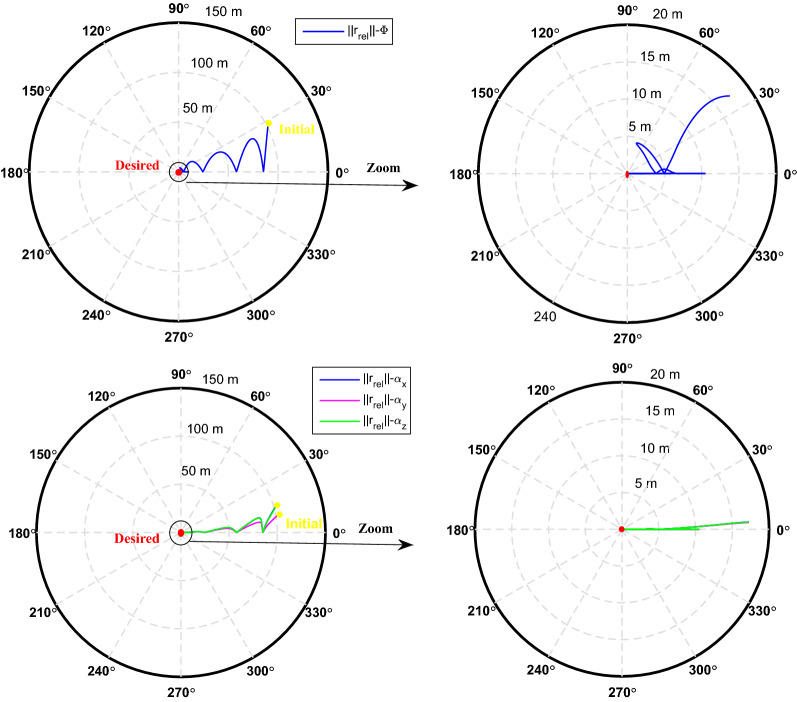
Relative pose trajectory in polar coordinate (Scenario 3).

The polar curve is plotted in Fig. 70 to further show the integrated relative pose trajectory.

## Conclusion

In this paper, two sets of variable structure control laws based on the sliding mode technique have been developed for the coupled translation and orientation (pose) motion control of a chaser spacecraft in the close-range autonomous rendezvous and docking phase. The spacecraft's nonlinear dynamics and kinematics (both translational and attitude motion) served as the foundation for the controller designs. In relative translational motion control, three time-varying sliding surfaces were chosen in a way that achieves more robustness and faster convergence compared to conventional SMC. The main benefit of the proposed time-varying sliding surface is eliminating the reaching phase in a way that keeps the states on the sliding surface even from the very beginning time; thus the system’s robustness is guaranteed from any initial condition. In relative attitude control, the RWs' role along with the thrusters' adverse torque are considered in the equations of motion. Again three nonlinear sliding surfaces are allocated in a way that provides a faster response than a conventional SMC (as a result of their strong tolerance for rising gain).

The robustness and performance of the proposed controllers were evaluated by comprehensive simulation based on three different scenarios, in the presence of (1) inertia, mass, control input, and measurement uncertainties, (2) actuators dynamics, misalignment, and saturation, and (3) external disturbances.

## Data Availability

The authors confirm that all the data supporting the findings of this study are available within the article.
